# Photoredox Cross-Dehydrogenative Coupling of *N*-Aryl Glycines Mediated by Mesoporous Graphitic Carbon Nitride: An Environmentally Friendly Approach to the Synthesis of Non-Proteinogenic α-Amino Acids (NPAAs) Decorated with Indoles

**DOI:** 10.1021/acs.joc.2c00474

**Published:** 2022-05-27

**Authors:** Lorenzo Poletti, Daniele Ragno, Olga Bortolini, Francesco Presini, Fabio Pesciaioli, Stefano Carli, Stefano Caramori, Alessandra Molinari, Alessandro Massi, Graziano Di Carmine

**Affiliations:** † Department of Chemical, Pharmaceutical and Agricultural Sciences, University of Ferrara, Via L. Borsari, 46, 44121 Ferrara, Italy; ‡ Department of Environmental and Prevention Sciences, University of Ferrara, Via L. Borsari, 46, 44121 Ferrara, Italy; § Department of Physical and Chemical Sciences, Università degli Studi dell’Aquila, Via Vetoio, 42, 67100 L’Aquila, Italy

## Abstract

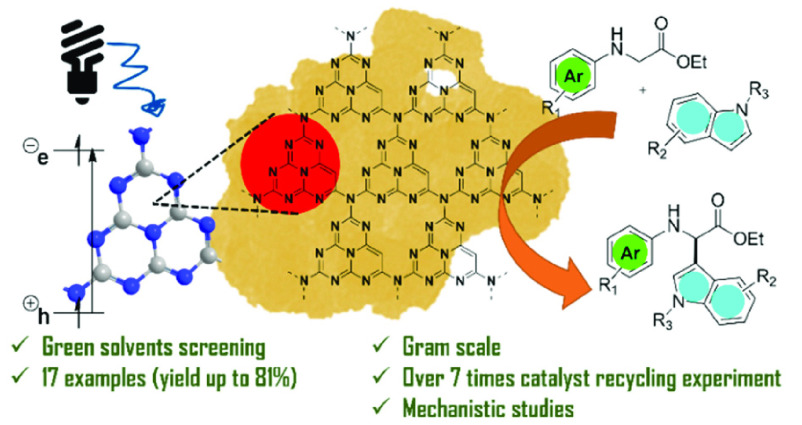

Indole-decorated glycine derivatives are prepared through an environmentally benign cross-dehydrogenative coupling between *N*-aryl glycine analogues and indoles (yield of ≤81%). Merging heterogeneous organocatalysis and photocatalysis, C–H functionalization has been achieved by selective C-2 oxidation of *N*-aryl glycines to afford the electrophilic imine followed by Friedel–Crafts alkylation with indole. The sustainability of the process has been taken into account in the reaction design through the implementation of a metal-free recyclable heterogeneous photocatalyst and a green reaction medium. Scale-up of the benchmark reaction (gram scale, yield of 69%) and recycling experiments (over seven runs without a loss of efficiency) have been performed to prove the robustness of the protocol. Finally, mechanistic studies were conducted employing electron paramagnetic resonance spectroscopy to unveil the roles of the photocatalyst and oxygen in the formation of odd-electron species.

## Introduction

One of the most intriguing inclinations of organic chemistry is to design reactions exploring novel pathways. This attitude not only is an exercise in style but also is driven by the need for robust synthetic platforms and more sustainable and efficient protocols.^1^ Among the chemical manufacturers, the pharmaceutical industry is lagging behind other industries in tackling the green transition because of the high value of their products.^2^ Nevertheless, the climate crisis is so serious that all sectors need to revise their production strategies to protect the planet. In the field of active pharmaceutical ingredients (APIs) and key intermediates for pharmaceuticals, many studies have been devoted to the replacement of toxic reagents and harmful materials with greener and safer chemicals for the development of more sustainable procedures. However, most of the efforts in this direction can be attributed to academia, and only a few industrial implementations have been reported.^3^

**Scheme 1:**
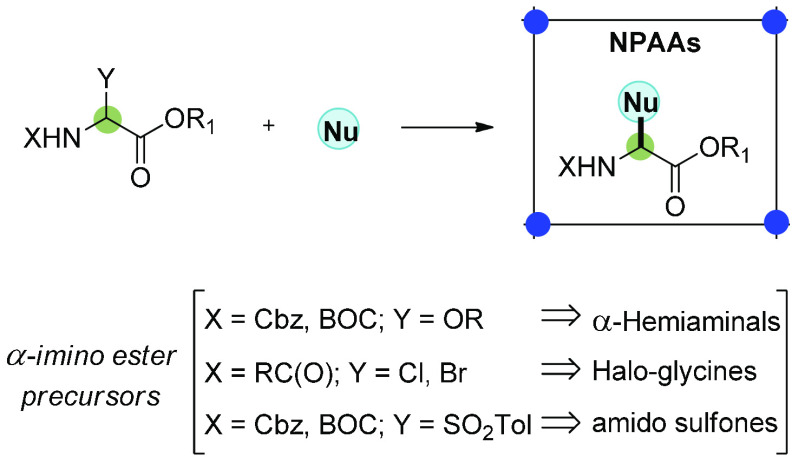
Direct Synthesis of NPAAs by In Situ Formation of α-Imino Esters from Classical Precursors

Both natural and unnatural non-proteinogenic α-amino acids (NPAAs) are of paramount importance for the pharmaceutical industry as components of therapeutic peptides.^4,5^ Additionally, natural NPAAs are often incorporated into complex natural products such as vancomycin, which is widely employed as an antibiotic in the treatment of infections.^6^ Unnatural NPAAs are also employed in conformational studies through Förster resonance energy transfer (FRET) experiments and in the design of new antimicrobial peptides (AMPs), which are promising candidates for overcoming bacterial resistance.^7,8^ A successful strategy for accessing NPAAs is the functionalization of α-imino ester precursors such as hemiaminals, α-haloglycines, and α-amido sulfones by carbon nucleophile addition (Scheme 1).^4,9^

**Scheme 2:**
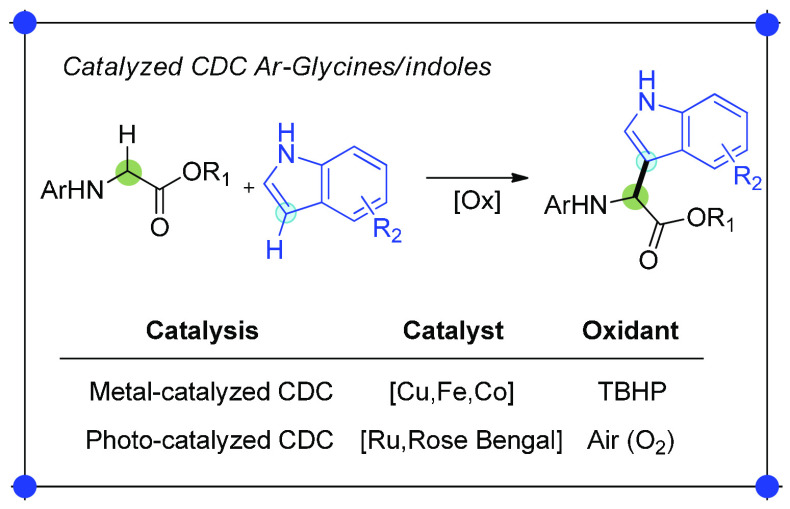
Metal-Catalyzed vs Photocatalyzed CDC of *N*-Aryl Glycine Derivatives and Indoles

The direct modification at the α-position of glycine derivatives by C–H activation is, however, preferable because this approach avoids byproduct formation and additional steps.^10^ A plethora of methods have been reported in the literature, including methylene activation by deprotonation with (super) bases,^11^ radical activation with di-*tert*-butyl peroxide through ultraviolet (UV) photolysis^12^ and cross-dehydrogenative coupling (CDC) reactions.^13−24^ The CDC consists of a first step in which the amino acid derivative is oxidized at the α-position enabling the formation of an iminium/imine species, followed by the interception of this intermediate by a carbon nucleophile to generate the new C–C bond.^25^ Several C-nucleophiles such as nitroalkanes, α-enolizable carbonyl/carboxyl compounds, and enamines proved to be good reaction partners in CDCs, making this approach highly appealing for expanding the library of unnatural NPAAs.^26^ Even though several protocols have been disclosed so far, new ways to perform CDC reactions of glycine derivatives are still attracting the attention of organic chemists with a particular focus on the process efficiency, sustainability, and molecular diversity of the products. The indole scaffold is of great interest because of its presence in many natural products and biologically active compounds, and thus, several CDCs of *N*-aryl glycines with indoles have been reported employing copper, cobalt, and iron catalysts.^17,18,20^ These approaches, however, require stoichiometric amounts of oxidants such as, for instance, di-*tert*-butyl hydroperoxide (TBHP). To overcome this limitation, photocatalysis has been successfully applied using metals and organic dyes with atmospheric oxygen as the terminal oxidant (Scheme 2).^27,28^

In recent years, photocatalysis has emerged as a fundamental pillar of modern catalysis allowing the discovery of novel reactivities on one hand and safer and greener procedures on the other.^29−32^ Since its renaissance in the field of organic chemistry thanks to the pioneering studies by Yoon, MacMillan, and Stephenson, photocatalysis has experienced dramatic growth characterized by the dominant use of transition metal catalysts.^33−35^ Nevertheless, the scarcity and increased cost of iridium and ruthenium, which today are classified as critical raw materials (CRMs), have made the search for new alternatives particularly compelling for the future. Although the use of organic molecular dyes have been attempted in CDCs, the short lifetime of their excited states may represent an important limitation for use in industrial applications. Recently, polymeric graphitic carbon nitrides (g-CNs) have become more important as photocatalysts because their heterogeneous nature enables their easy separation from the crude after reaction and recycling.^36−41^ g-CNs consist of C, N, and a small amount of H, and they can be easily prepared from cheap and widely available materials.^42−56^ The repetitive unit of g-CNs, the tri-*s*-triazine or heptazine core, allows for the formation of planar layers through π-stacking interactions, making the resulting material graphitic. Significantly, the electronic features of g-CNs can be suitably tuned by modification of the morphology and crystallinity as well as by doping with other elements.^42,57−60^

In this work, we have investigated mesoporous graphitic carbon nitride (mpg-CN) as a heterogeneous organocatalyst in the light-driven CDC of *N*-aryl glycines with indoles to access indole-decorated unnatural NPAAs, focusing our attention on sustainability issues and mechanistic insights.

## Results and Discussion

At the beginning of our study, we envisaged graphitic carbon nitride (g-CN), mesoporous graphitic carbon nitride (mpg-CN), and potassium poly(heptazine imides) (K-PHI) as good candidates for the promotion of the model CDC of ethyl 2-(phenylamino)acetate **1a** and indole **2a** (Table 1). As metal-free, thermostable, heterogeneous organocatalyts, CN semiconductors are privileged mediators in solid-state photocatalysis; indeed, their electronic features allow them to act as a reductant and/or an oxidant upon light irradiation, and the smaller band gap (∼2.7 eV corresponding to the ultraviolet–visible region) allows CNs to operate under milder conditions compared to those of TiO_2_. In this study, g-CN and mpg-CN were prepared from cyanamide according to the procedures described by Reisner and Zhu,^46,48^ while K-PHI was synthesized following the protocol reported by the group of Savateev.^61^

**Table 1:**
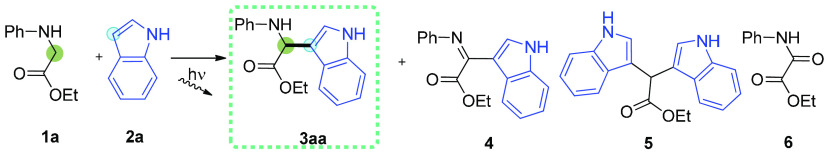
Optimization of **1a/2a** Coupling in Conventional Solvents^a^

entry	solvent	catalyst	time	additive	conversion (%)^b^	**3aa** (%)^b^	**4** (%)^b^	**5** (%)^b^	**6** (%)^b^
1^c^	ACN	g-CN	72	Zn(OAc)_2_	≤5	≤5	–	–	–
2^c^	ACN	g-CN	72	Sc(OTf)_2_	≤5	≤5	–	–	–
3^c^	ACN	K-PHI	72	Zn(OAc)_2_	0	–	–	–	–
4^c^	ACN	mpg-CN	72	Zn(OAc)_2_	12	12	–	–	–
5^d^	ACN	mpg-CN	72	Zn(OAc)_2_	28	23	–	5	–
6	ACN	mpg-CN	72	Zn(OAc)_2_	47	21	11	11	≤5
7^e^	ACN	mpg-CN	72	Zn(OAc)_2_	64	24	30	6	≤5
8	ACN	mpg-CN	72	–	100	54	32	7	7
9	ACN	mpg-CN	16	–	85	58	15	7	5
10^f^	ACN	mpg-CN	16	–	0	–	–	–	–
11^f^	ACN	–	16	–	0	–	–	–	–
12	ACN	–	16	–	8	≤5	–	–	≤5
13^g^	ACN	mpg-CN	16	–	≤5	≤5	–	–	–
14	DMF	mpg-CN	16	–	72	39	30	–	3
15	THF	mpg-CN	16	–	85	37	–	16	32
16	DCM	mpg-CN	16	–	70	35	19	16	–
17	DMSO	mpg-CN	16	–	41	13		28	–
18	toluene	mpg-CN	16	–	87	50	15	22	–

a For the reaction, **1a** (0.1 mmol), **2a** (0.13 mmol), 1 mL of solvent, 20 mol % additive (when present), and 10 mg of catalyst were placed in a 5 mL vial equipped with a magnetic bar and a balloon filled with air; the reaction mixture was stirred under 40 W blue LED light for the time reported.

b Conversion of **1a** and yields were determined by ^1^H NMR using durene as an internal standard.

c Reaction performed with a 10 W fluorescent light bulb.

d Reaction performed with 10 W blue LED light.

e With 10 mol % additive.

f Reaction performed in the dark.

g Reaction performed under argon.

Inspired by the previous work of Rueping et al.,^27^ we initially tested g-CN in the CDC of **1a** and **2a** using acetonitrile as the solvent and 20 mol % zinc acetate as the Lewis acid additive under light irradiation by a 10 W fluorescent bulb lamp (entry 1). According to the studies reported by the groups of Rueping^24^ and Neogi,^62^ zinc acetate is necessary to activate, through chelation, the imine intermediate formed by oxidation of *N*-phenyl glycine derivative **1a**, allowing the coupling with indole to proceed smoothly. Unfortunately, under these conditions, the desired product **3aa** was detected in only trace amounts after a long reaction time. Replacing zinc acetate with scandium triflate left the reaction outcome unchanged (entry 2). K-PHI showed no reactivity under the same conditions (entry 3). A slight but significant improvement was observed employing mpg-CN (conversion of 12%, 11% yield of **3aa**; entry 4). The most important feature of mpg-CN is the much larger surface area (∼200 m^2^ g^–1^) compared to that of the graphitic counterpart (∼5 m^2^ g^–1^) that maximizes the interaction of reactants with the catalyst. Furthermore, because UV diffuse reflectance spectroscopy of mpg-CN showed the onset in the UV region and a maximum in absorbance around 350 nm (see page S3 of the Supporting Information for further details), the reaction vial was irradiated with a 10 W blue LED. Gratifyingly, an increase in both conversion (28%) and the yield of **3aa** (23%) was observed with the monochromatic lamp (entry 5). Furthermore, an increment of the LED power from 10 to 40 W resulted in a higher conversion (47%) but a similar yield of **3aa** (21%; entry 6). Indeed, we found that side products **4–6** appeared when the conversion increased. Reasonably, imine **4** is formed by oxidation of **3aa** in analogy to **1a** activation, while **5** might derive from the photoassisted C–N cleavage of **3aa** followed by addition of a second molecule of indole **2a**. On the contrary, it is likely that α-amido ester **6** is produced by overoxidation of **1a** with aerial oxygen. Interestingly, we observed that the conversion increased when the loading of the additive Zn(OAc)_2_ was reduced to 10 mol % (conversion of 64%, 24% yield of **3aa**; entry 7), albeit the formation of imine **4** became more significant (30%) under these conditions. Following this observation, we performed model coupling without an additive, detecting an important improvement in reaction efficiency (conversion of 100%, 54% yield of **3aa**; entry 8). A reasonable explanation of this result is that the Lewis acid partially deactivates the mpg-CN; at the same time, the amine terminal groups of mpg-CN may act as H-bond donors, increasing the electrophilicity of the intermediate imine and thus compensating for the absence of the additive. A decrease in the reaction time from 72 to 16 h gave a satisfactory conversion (85%) and a better selectivity (58% yield of **3aa**; entry 9). Next, the synergistic action of mpg-CN and light was confirmed by some blank experiments. The reaction did not proceed in the dark with or without the catalyst (entries 10 and 11). A low conversion of 8% with the main formation of byproduct **6** was observed upon irradiation of the reaction mixture in the absence of mpg-CN (entry 12). This result suggests that the excited state of *N*-phenyl glycine ethyl ester **1a** can undergo oxidation by molecular oxygen; however, fast recombination occurs, and formation of the desired product **3aa** is limited. Additionally, we established that the reaction does not take place in the absence of molecular oxygen (entry 13). At this stage, we proceeded with the screening of typical organic solvents (entries 14–18), observing a comparable level of conversion in THF (85%; entry 15) and toluene (87%; entry 18) as in acetonitrile (entry 9) accompanied, however, by lower **3aa** yields (37% and 50%) as a result of poorer selectivity.

The catalytic activity of mpg-CN was also tested in selected green solvents^63,64^ with the aim of improving the sustainability of the disclosed CDC process (Table 2). The rate of **1a/2a** coupling in acetone slightly decreased, affording **3aa** in 55% yield after 16 h; notably, the formation of side products **5** and **6** seemed to be inhibited in this solvent (entry 1). The model reaction did not occur in ethanol (entry 2), while a low conversion and an only 30% yield of **3aa** were observed in water after 72 h (entry 3). The utilization of a 2:1 EtOH/H_2_O mixture accelerated the reaction because of a better dissolution of the starting materials, but **3aa** was formed in only 12% yield (entry 4). Ethyl acetate gave the better outcome in terms of conversion efficiency, reaction rate, and **3aa** yield (conversion of 100%, 64% yield of **3aa**; entry 5), whereas the biomass-derived Me-THF (entry 6) showed a behavior similar to that of THF (Table 1, entry 15). Interestingly, the CDC procedure proved to be compatible with the emerging green solvents (+)-limonene (LIM) and γ-valerolactone (GVL), affording **3aa** in 51% and 45% yields, respectively (entries 7 and 8, respectively), whereas dimethyl isosorbide (DIM) completely inhibited the model coupling (entry 9). Having selected EtOAc as the optimal solvent and considered that the overoxidation side path could be limited, we reversed the reaction stoichiometry using a slight excess of **1a** (0.13 equiv); as expected, the yield of **3aa** slightly increased from 64% (entry 5) to 69% (entry 10). A further improvement was obtained by setting the power of irradiation at 20 W (71% yield of **3aa**; entry 11), whereas further reducing the light power led to a decrease in reactivity (entry 12). Finally, in agreement with the observation by Reisner and co-workers,^46^ we found that an increase in catalyst loading decreased the process efficiency (entry 14), likely because of a lower absorption of photons by the cloudy mpg-CN suspension. Halving the catalyst loading to 5 mg led to a large decrease in reactivity (entry 13).

**Table 2:** Optimization of **1a/2a** Coupling in Sustainable Solvents Promoted by mpg-CN^a^

entry	solvent	time	conversion (%)^b^	**3aa** (%)^b^	**4** (%)^b^	**5** (%)^b^	**6** (%)^b^
1	acetone	16	74	55	19	–	–
2	EtOH	16	0	–	–	–	–
3	H_2_O	72	42	30	–	–	12
4	EtOH/H_2_O (2:1)	48	46	12	32	≤5	–
5	EtOAc	16	100	64	15	11	10
6	Me-THF	16	81	50	9	13	9
7	LIM	48	72	51	8	8	5
8	GVL	16	55	45	–	–	10
9	DMI	48	0	–	–	–	–
10^c^	EtOAc	16	100	69	13	9	9
11^c^,^d^	EtOAc	16	100	71	12	7	10
12^c^,^e^	EtOAc	16	65	54	≤5	≤5	≤5
13^c^,^d^,^f^	EtOAc	16	13	≤5	≤5		8
14^c^,^d^,^g^	EtOAc	16	20	7	≤5		11

a For the reaction, **1a** (0.1 mmol), **2a** (0.13 mmol), 1 mL of solvent, and 10 mg of catalyst were placed in a 5 mL vial equipped with a magnetic bar and a balloon filled with air; the reaction mixture was stirred under 40 W blue LED light for the time reported.

b Conversion of **1a** and yields of **3aa** and **4–6** were determined by ^1^H NMR using durene as an internal standard.

c Reaction performed with **1a** (0.13 mmol) and **2a** (0.1 mmol).

d Reaction performed with 20 W blue LED light.

e Reaction performed with 10 W blue LED light.

f Reaction performed with 5 mg of mpg-CN.

g Reaction performed with 15 mg of mpg-CN.

With the optimal conditions in hand (entry 11, Table 2), we moved to investigate the scope of the reaction. Several *N*-aryl glycine derivatives **1** and indoles **2** were tested by varying the stereoelectronic features of substituents on both reactants (Table 3). A satisfactory level of efficiency was found in the CDCs of indole **2a** and *para*-substituted *N*-aryl glycines **1a–1f**, affording target products **3aa–3fa** in 65–79% yields. Similar outcomes were also detected with *ortho*- and *meta*-substituted *N*-aryl glycines **1i** and **1j**, which gave the corresponding amino esters **3ia** and **3ja** in 58% and 62% yields, respectively. It is noteworthy that these results suggest that the oxidative potential of photoexcited mpg-CN is suitable to activate a wide range of electronically diversified *N*-aryl glycines **1**. Disappointingly, the steric hindrance of the aryl substituent of glycines **1** was proven to strongly affect the selectivity toward the desired products **3**. Indeed, the CDC of indole **2a** with glycines **1g** and **1h** bearing a disubstituted phenyl ring resulted in the full conversion of **1g** and **1h** into the corresponding α-amido ester **6** (major product) and imine **4** (minor product), with no formation of the expected amino esters **3ga** and **3ha**. A reasonable explanation for this outcome is that the photoredox process takes place forming the imine intermediate, which, however, is too hindered to undergo the C–C bond formation with indole through the Friedel–Crafts pathway. With respect to variation of indole **2**, we found that electron-poor indoles were more reactive (**3ae–3ag**, 62–81% yields) than their electron-rich counterparts (**3ab–3ad**, 56–65%), probably because of the higher acidity of the indole H3 involved in the rearomatization step. Additionally, it appeared that the reactivity of N-substituted indoles was deeply affected by the electronic properties of the group of nitrogen (**3ah–3aj**, 10–73%). Moreover, other amine derivatives such as ethyl 2-(butylamino)acetate, ethyl 2-(1,3-dioxoisoindolin-2-yl)acetate, *N*,*N*-dimethylaniline, and 2-phenyl-1,2,3,4-tetrahydroisoquinoline have been unsuccessfully tested (see page S17 of the Supporting Information for structures). On the contrary, less nucleophilic electron-rich arenes such as 2-methylfuran and 2-methoxyphenol did not afford the desired products and only **6** has been observed in the reaction crude (see page S17 of the Supporting Information for structures).

**Table 3:**
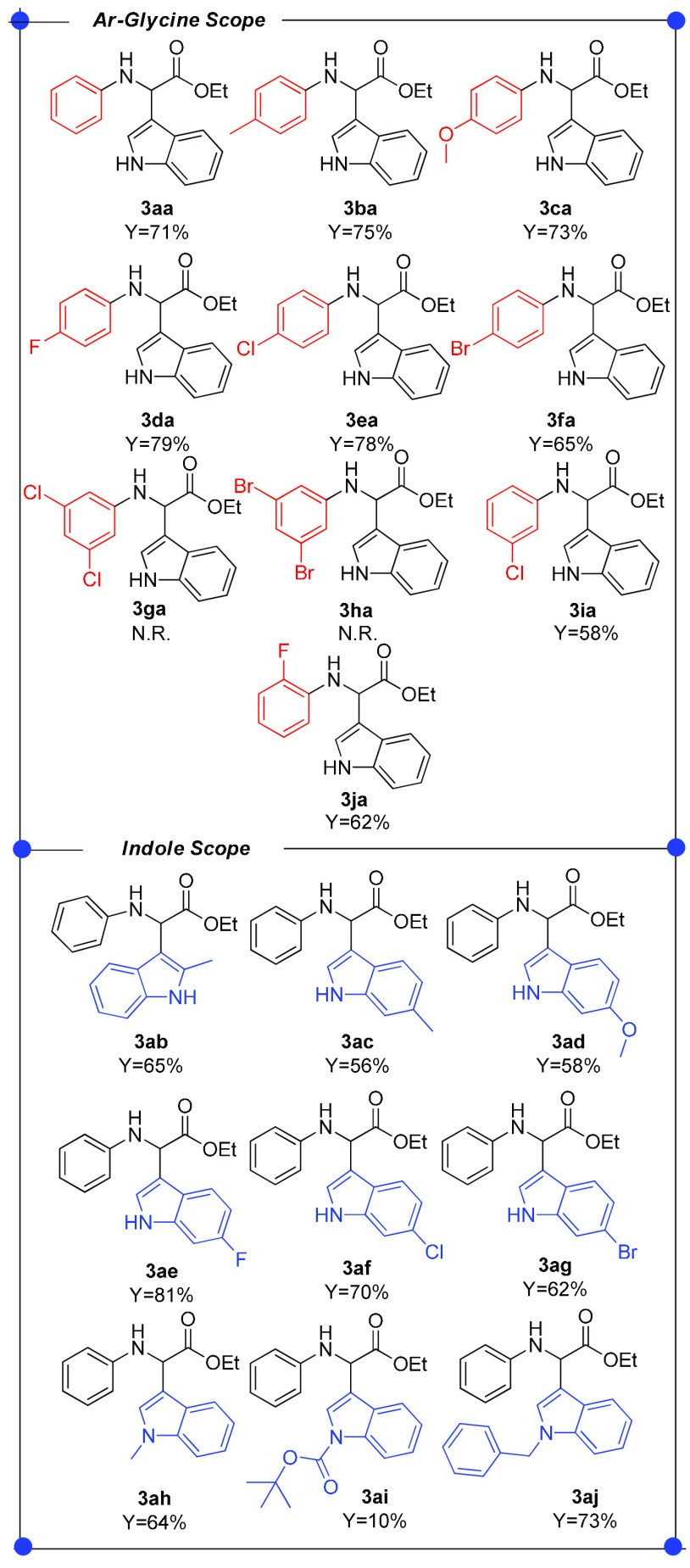
Scope of the Light-Driven CDC of *N*-Aryl Glycine Derivatives **1** with Indoles **2** Promoted by mpg-CN^a^

a For the reaction (General Procedure A in the Experimental Section), **1** (0.13 mmol), **2** (0.1 mmol), 1 mL of EtOAc, and 10 mg of mpg-CN were placed in a 5 mL vial equipped with a magnetic bar and a balloon filled with air; the reaction mixture was stirred under 20 W blue LED light for 16 h. For compounds **3ab–3ad** and **3ah**, an excess of indole **2** has been employed (General Procedure B in the Experimental Section).

**Scheme 3:**
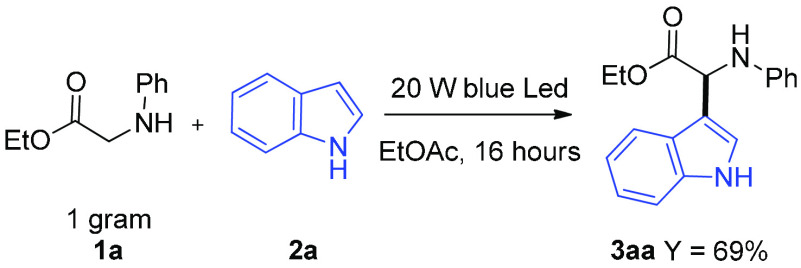
Gram-Scale Experiment

Remarkably, the CDC of **1a** and **2a** could be scaled up to the gram scale (Scheme 3) without affecting the efficiency of the process (69% yield of **3aa**, 3.0 mmol, 872 mg).

**Figure 1:**
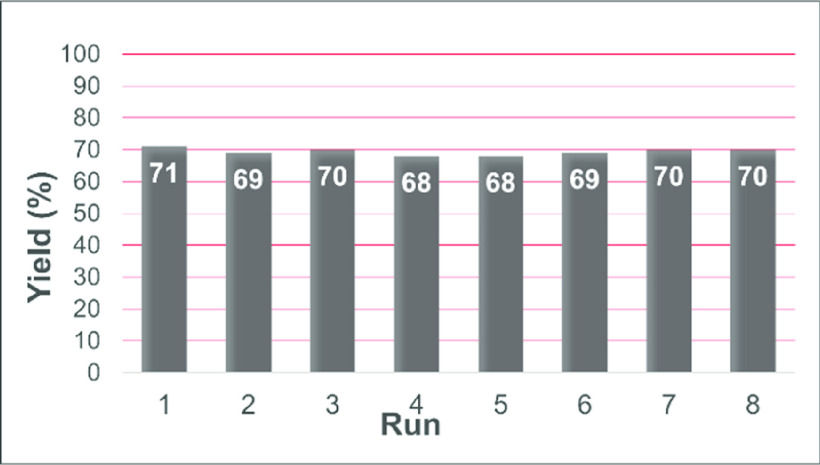
Recycling experiments (**1a/2a** coupling promoted by mpg-CN).

Subsequently, the recyclability of mpg-CN was investigated over seven runs. After each reaction (**1a/2a** coupling), the catalyst was simply collected by filtration, washed with a small portion of ethyl acetate, and dried over vacuum (40 °C) for 4 h. As shown in Figure 1, mpg-CN maintained the same activity, proving its robustness in terms of reuse.

**Figure 2:**
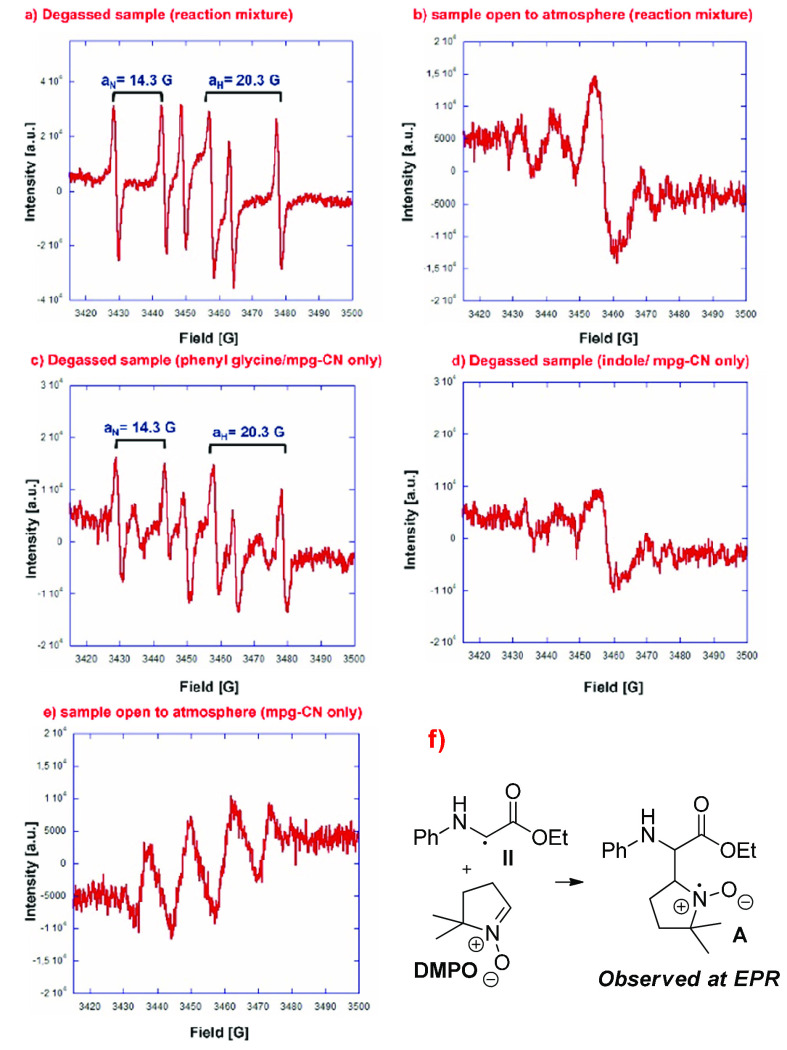
EPR experiments carried out with DMPO as the radical trap (5 × 10^–2^ M in ACN, 1 mL): (a) **1a** (0.13 mmol), **2a** (0.1 mmol), and mpg-CN (10 mg) under N_2_ in a degassed solvent, (b) **1a** (0.13 mmol), **2a** (0.1 mmol), and mpg-CN (10 mg) open to the atmosphere, (c) **1a** (0.13 mmol) and mpg-CN (10 mg) under N_2_ in a degassed solvent, (d) **2a** (0.1 mmol) and mpg-CN (10 mg) under N_2_ in a degassed solvent, and (e) mpg-CN (10 mg) open to the atmosphere.

**Scheme 4:**
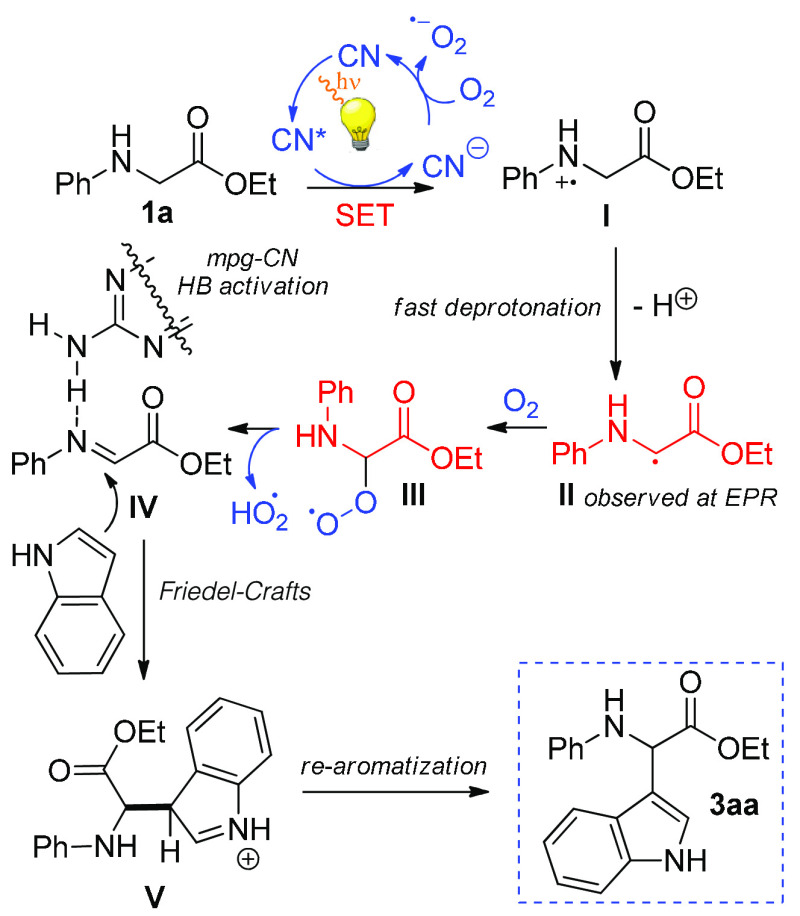
Proposed Mechanism

**Scheme 5:**
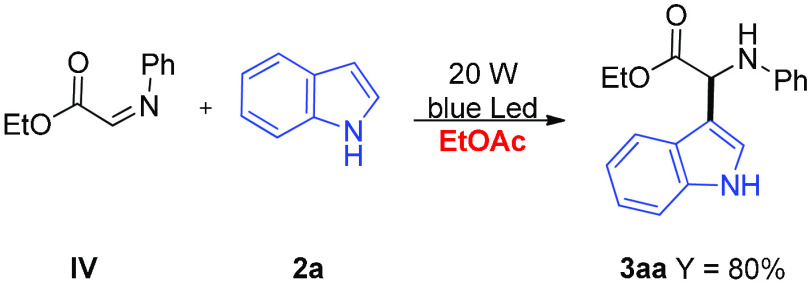
Reaction between Preformed **IV** and Indole **2a**

Finally, a mechanistic investigation was performed to illuminate the role of oxygen and mpg-CN in the CDC path with the aid of EPR and dedicated synthetic experiments. The EPR spectrum of the degassed reaction mixture (**1a**, **2a**, mpg-CN, and acetonitrile) irradiated in the presence of the spin trapper 5,5-dimethyl-1-pyrroline *N*-oxide (DMPO) clearly showed the formation of a radical adduct (Figure 2a). According to the literature,^65^ the hyperfine splitting constants (*A*_N_ and *A*_H_) of the detected radical suggested the reaction of carbon-centered radical **II** with DMPO to form adduct **A** (Figure 2f). One can speculate that trapped radical **II** is generated by single-electron transfer (SET) from the nitrogen lone pair of *N*-aryl glycine **1a** to the excited mpg-CN [CN* (Scheme 4)], followed by fast deprotonation at C2 of resulting radical cation **I**. Although recent works have reported the back-electron transfer (BET) of aminium radicals as a competitive process, it is also known that the kinetics of deprotonation can be favored by an increase in the C–H acidity, which could happen in the case of amino ester **1a**.^66−73^ The EPR signals disappeared in the aerated sample (Figure 2b), reasonably because of the fast conversion of radical **II** into the even-electron iminium **IV** through intermediate **III** (radical hydroperoxide) in the presence of oxygen (Scheme 4). EPR control experiments also revealed that redox process involves only *N*-aryl glycine **1a** and mpg-CN (Figure 2a,c–e), thus confirming that indole **2a** is engaged in the CDC reaction after iminium **IV** formation (Scheme 4); this key intermediate is likely involved in C–C bond formation because we observed **3aa** in high yield employing preformed **IV** under our experimental conditions (Scheme 5). Furthermore, recently the CN has been proven to accumulate negative charge during the redox process, which corroborates the partial activity of the catalyst, not versus product formation, but at least toward the redox step in the absence of a regenerating oxidant.^74^

**Scheme 6:**
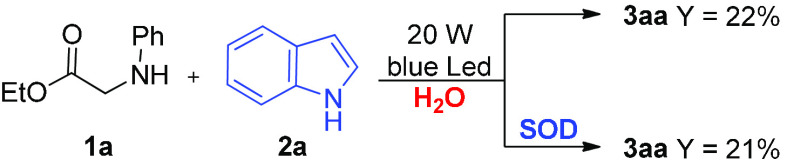
CDC Promoted by mpg-CN in the Presence or Absence of Superoxide Dismutase (SOD)

The role of the superoxide radical anion [O_2_]^•–^, which is generated by oxidation of the reduced mpg-CN (CN^–^), was investigated by performing the model **1a/2a** coupling in water (Table 2, entry 3) with the addition of superoxide dismutase (SOD) as the superoxide scavenger (Scheme 6). Because the conversion of phenyl glycine **1a** into product **3aa** remained unaltered in the presence SOD, it can be hypothesized that [O_2_]^•–^ is not involved in the direct formation of imine **IV** but efficiently regenerates the catalyst and increases the turnover number. However, oxygen remains crucial in imine generation (Table 1, entry 13); thus, we hypothesized, according to the literature,^72^ that imine comes from intermediate **III**, which is formed by interception of intermediate **II** by molecular oxygen. Finally, we carried out qualitative tests to detect the eventual presence of H_2_O_2_, which can be involved in oxygenative pathways. A solution of iron(III) chloride and EDTA has been added to the mixture for the crude reaction performed in water, following the protocol disclosed by Pelit,^75^ giving negative results. However, the test cannot completely exclude the presence of hydrogen peroxide in the mechanism due to possible fast degradation that, if quicker than formation, will make its detection impossible. It is important to emphasize, however, that the overall mechanistic picture depicted in Scheme 4 is the result of our experimental observations and literature data, but alternative pathways should not be fully excluded.

In conclusion, an environmentally benign CDC of aryl glycine derivatives with indoles has been reported. The sustainability of the process is determined by employing a heterogeneous organo-photocatalyst in a green medium. Furthermore, the ability of the catalyst to work with atmospheric oxygen and its excellent recyclability make the reported protocol attractive for industrial purposes. The reaction scope of CDC has also been investigated; the protocol has proven to be robust and versatile covering both electron-poor and electron-rich substrates and showing limits only on sterically hindered substrates. Recycling tests revealed that the catalyst maintains its high efficiency over at least seven runs. The reaction was scaled up to the gram scale. Finally, mechanistic studies have been performed via EPR spectroscopy, showing that the superoxide ion is manly involved in the regeneration of the catalyst to increase the turnover number.

## Experimental Section

**General Experimental Methods.** Commercially available reagents were purchased from commercial sources and used without any subsequent purification. The solvents used for reactions were distilled from appropriate drying agents and stored over 3 Å molecular sieves. ^1^H, ^13^C, and ^19^F NMR spectra were recorded on Varian Mercury Plus 300 and Varian Mercury Plus 400 spectrometers in CDCl_3_ and acetone-*d*_6_ at room temperature. ^13^C{^1^H} NMR spectra were recorded in ^1^H broad-band decoupled mode, and chemical shifts (δ) are reported in parts per million relative to the residual solvent peak. The EPR spin trapping survey was carried out with a Bruker model ER 200 MRD spectrometer equipped with a model TE 201 resonator equipped with a medium-pressure Hg lamp. Reactions were monitored by TLC on silica gel 60 F254. Flash column chromatography was performed on silica gel 60 (230–400 mesh). High-resolution mass spectra (HRMS) were recorded in positive ion mode by an Agilent 6520 HPLC-Chip Q/TF-MS nanospray instrument using a time-of-flight, a quadrupole, or a hexapole unit to produce spectra. The SEM images were recorded using the SEM Zeiss EVO 40 scanning electron microscope. The TEM images were recorded with the TEM Zeiss EM 900 transmission electron microscope. The blue LEDs (10 W, 465 nm) used for the synthesis of the products were purchased from Aftertech s.a.s. Diffuse reflectance absorption spectra were recorded with a JASCO V-570 spectrophotometer with an integrating sphere. Emission spectra were recorded on an Edinburgh Instruments FS920 steady-state spectrofluorimeter configured with a TMS300-X single-excitation monochromator (300 mm focal length, f/4.1 aperture) and a double-emission monochromator consisting of two coupled 300 mm focal length, f/4.1 monochromators to reduce stray light and increase spectral resolution. Samples for cyclic voltammetry were prepared by drop casting a mixture of CNs in ACN (10 mg/mL) on FTO glass. Cyclic voltammograms were recorded in 0.1 N LiClO_4_/ACN and a standard three-electrode cell with an Autolab PGSTAT 302/N potentiostat, at a scan rate of 100 mV s^–1^. A standard calomel electrode (SCE) and a Pt wire were used as the reference and auxiliary electrodes, respectively. FTO or glassy carbon was used as the working electrode in the case of cyclic voltammetry analysis of CNs or phenyl glycine, respectively.

### Procedure for the Preparation of mpg-CN (mesoporous graphitic carbon nitride)

Cyanamide (3.0 g) and Ludox HS-40 (7.5 g) were mixed in a glass vial and stirred at room temperature for 30 min until the cyanamide had completely dissolved. The obtained solution was stirred at 60 °C for 16 h until the water had completely evaporated. The magnetic stir bar was removed, and the solid transferred in a crucible and heated under a nitrogen flow in an oven. The temperature was increased from room temperature to 550 °C (muffle furnace) in 4 h and maintained for additional 4 h. The crucible was then cooled to room temperature under a nitrogen flow. The solid was ground in a mortar and transferred in a polypropylene flask. A solution of (NH_4_)HF_2_ was added, and the suspension was stirred at room temperature for 24 h. The solid was filtered, washed once with water and once with ethanol, and then dried under vacuum (60 °C, 20 mbar) overnight. For characterization and for more details, see the Supporting Information.^46^

### Procedure for the Preparation of g-CN (graphitic carbon nitride)

Cyanamide (3 g) was treated with a solution of NaOH (1 M), dried, and placed in a crucible, which was covered with a lid, transferred to an oven, and heated at 550 °C (muffle furnace) with a heating rate of 2.3 °C min^–1^ under a nitrogen flow. Then, the crucible was slowly cooled to room temperature under a nitrogen flow. The product was finely ground in a marble mortar and collected. Characterization showed its data were in line with the literature.^46^

### Procedure for the Preparation of K-PHI [potassium poly(heptazine imide)]

Lithium chloride (3.71 g), potassium chloride (4.54 g), and 5-aminotetrazole (1.65 g) were mixed and ground in a marble mortar. The reaction mixture was transferred to a porcelain crucible, which was covered with a lid. The crucible was placed in an oven and heated under a nitrogen flow (15 L min^–1^) at atmospheric pressure with the following heating program: heated from room temperature to 550 °C (muffle furnace) for 4 h and this final temperature maintained for an additional 4 h. Then, the crucible was slowly cooled to room temperature under a nitrogen flow. The raw product was then removed from the crucible, washed for 3 h with deionized water (100 mL) to remove any traces of salts, filtered, and dried in a vacuum oven (20 mbar) at 50 °C overnight. Characterization showed its data were in line with the literature.^61^

#### General Procedure A for the Preparation of Phenyl Glycine (**1a–1f**, **1i**, and **1j**)

In a two-neck round-bottom flask under an argon atmosphere, a mixture of aniline (10 mmol, 1 equiv), ethyl chloroacetate (12 mmol, 1.2 equiv), and sodium acetate (10 mmol, 1 equiv) in anhydrous ethanol (10 mL, 1 M) was refluxed (oil bath) for 24 h. Then water was added (15 mL), and the aqueous layer was extracted three times with ethyl acetate. The organic layer was dried with anhydrous Na_2_SO_4_, filtered, and concentrated on the rotary evaporator. The crude product was purified by flash chromatography to give the final product as an amorphous solid.

#### General Procedure B for the Preparation of Phenyl Glycine (**1g** and **1h**)

In a two-neck round-bottom flask under an argon atmosphere, a mixture of aniline (10 mmol), ethyl chloroacetate (12 mmol), and *N*,*N*-diisopropylethylamine (20 mmol) in anhydrous ethanol (10 mL, 1 M) was refluxed (oil bath) for 24 h. Then water was added (15 mL), and the aqueous layer was extracted three times with ethyl acetate. The organic layer was dried with anhydrous Na_2_SO_4_, filtered, and concentrated on the rotary evaporator. The crude product was purified by flash chromatography to give the final product as an amorphous solid.

##### Ethyl Phenylglycinate (**1a**)

By following General Procedure A, **1a** (1.15 g, 6.4 mmol, 64%) was obtained as a yellow amorphous solid after column chromatography on silica gel (8:2 cyclohexane/EtOAc). ^1^H NMR (300 MHz, CDCl_3_): δ 7.20 (t, *J* = 8.2 Hz, 2H), 6.75 (t, *J* = 8.2 Hz, 1H), 6.62 (d, *J* = 8.2 Hz, 2H), 4.19–4.33 (m3H, CH_2_ and NH), 3.90 (s, 2H), 1.30 (t, *J* = 7.3 Hz, 3H). ^13^C{^1^H} NMR (101 MHz, CDCl_3_): δ 171.0, 146.8, 129.3, 118.4, 113.2, 61.3, 46.0, 14.2. HRMS (ESI) *m*/*z*: [M + H]^+^ calcd for C_10_H_14_NO_2_ 180.1019, found 180.1022.

##### Ethyl *p*-Tolylglycinate (**1b**)

By following General Procedure A, **1b** (1.31 g, 6.8 mmol, 68%) was obtained as a yellow amorphous solid after column chromatography on silica gel (8:2 cyclohexane/EtOAc). ^1^H NMR (300 MHz, CDCl_3_): δ 7.00 (d, *J* = 8.4 Hz, 2H, Ar), 6.54 (d, *J* = 8.4 Hz, 2H), 4.79 (s, 1H), 4.23 (q, *J* = 7.1 Hz, 2H), 3.88 (s, 2H), 2.24 (s, 3H), 1.29 (t, *J* = 7.1 Hz, 3H). ^13^C{^1^H} NMR (101 MHz, CDCl_3_): δ 171.0, 144.15, 129.8, 128.1, 113.7, 61.3, 46.5, 20.4, 14.2. HRMS (ESI) *m*/*z*: [M + H]^+^ calcd for C_11_H_16_NO_2_ 194.1176, found 194.1169.

##### Ethyl (4-Methoxyphenyl)glycinate (**1c**)

By following General Procedure A, **1c** (1.48 g, 7.1 mmol, 71%) was obtained as a yellow amorphous solid after column chromatography on silica gel (8:2 cyclohexane/EtOAc). ^1^H NMR (300 MHz, CDCl_3_): δ 6.79 (d, *J* = 8.9 Hz, 2H), 6.59 (d, *J* = 8.9 Hz, 2H), 4.23 (q, *J* = 7.1 Hz, 2H), 4.02 (s, 1H), 3.86 (s, 2H), 3.75 (s, 3H), 1.29 (t, *J* = 7.1 Hz, 3H). ^13^C{^1^H} NMR (101 MHz, CDCl_3_): δ 171.3, 152.6, 141.2, 114.9, 114.3, 61.2, 55.7, 46.8, 14.2. HRMS (ESI) *m*/*z*: [M + H]^+^ calcd for C_11_H_16_NO_3_ 210.1125, found 210.1118.

##### Ethyl (4-Fluorophenyl)glycinate (**1d**)

By following General Procedure A, **1d** (1.24 g, 6.3 mmol, 63%) was obtained as a yellow amorphous solid after column chromatography on silica gel (8.5:1.5 cyclohexane/EtOAc). ^1^H NMR (300 MHz, CDCl_3_): δ 6.90 (t, *J* = 8.8 Hz, 2H), 6.55 (dd, *J* = 8.8, 4.4 Hz, 2H), 4.24 (q, *J* = 7.1 Hz, 2H), 4.17 (s, 1H), 3.86 (d, *J* = 5.3 Hz, 2H), 1.29 (t, *J* = 7.1 Hz, 3H). ^13^C{^1^H} NMR (101 MHz, CDCl_3_): δ 171.0, 157.4, 143.4, 115.9, 115.6, 113.9, 113.8, 61.3, 46.4, 14.2. ^19^F NMR (376 MHz, CDCl_3_): δ −127.2. HRMS (ESI) *m*/*z*: [M + H]^+^ calcd for C_10_H_13_FNO_2_ 198.0925, found 198.0919.

##### Ethyl (4-Chlorophenyl)glycinate (**1e**)

By following General Procedure A, **1e** (1.30 g, 6.1 mmol, 61%) was obtained as a white amorphous solid after column chromatography on silica gel (8:2 cyclohexane/EtOAc). ^1^H NMR (300 MHz, CDCl_3_): δ 7.14 (d, *J* = 8.9 Hz, 2H), 6.53 (d, *J* = 8.9 Hz, 2H), 4.25 (q, *J* = 7.1 Hz, 2H), 3.87 (d, *J* = 5.5 Hz, 2H), 1.30 (t, *J* = 7.1 Hz, 3H). ^13^C{^1^H} NMR (101 MHz, CDCl_3_): δ 170.8, 145.5, 129.1, 122.8, 114.0, 61.4, 45.8, 14.12. HRMS (ESI) *m*/*z*: [M + H]^+^ calcd for C_10_H_13_ClNO_2_ 214.0629, found 214.0635.

##### Ethyl (4-Bromophenyl)glycinate (**1f**)

By following General Procedure A, **1f** (1.53 g, 5.9 mmol, 59%) was obtained as a white amorphous solid after column chromatography on silica gel (8.5:1.5 cyclohexane/EtOAc). ^1^H NMR (300 MHz, CDCl_3_): δ 7.27 (d, *J* = 8.8 Hz, 2H), 6.49 (d, *J* = 8.8 Hz, 2H), 4.35–4.19 (m, 3H, CH_2_ and NH), 3.86 (d, *J* = 5.5 Hz, 2H), 1.30 (t, *J* = 7.1 Hz, 3H). ^13^C{^1^H} NMR (101 MHz, CDCl_3_): δ 170.7, 146.0, 132.0, 114.5, 109.9, 61.4, 45.7, 14.2. HRMS (ESI) *m*/*z*: [M + H]^+^ calcd for C_10_H_13_BrNO_2_ 258.0124, found 258.0131.

##### Ethyl (3,5-Dichlorophenyl)glycinate (**1g**)

By following General Procedure B, **1g** (1.43 g, 5.8 mmol, 58%) was obtained as a yellow amorphous solid after column chromatography on silica gel (8.5:1.5 cyclohexane/EtOAc). ^1^H NMR (300 MHz, CDCl_3_): δ 6.71 (t, *J* = 1.8 Hz, 1H), 6.46 (d, *J* = 1.7 Hz, 2H), 4.48 (s, 1H), 4.26 (q, *J* = 7.1 Hz, 2H), 3.85 (d, *J* = 5.2 Hz, 2H), 1.31 (t, *J* = 7.1 Hz, 3H). ^13^C{^1^H} NMR (101 MHz, CDCl_3_): δ 170.2, 148.5, 135.5, 117.9, 111.1, 61.7, 45.3, 14.2. HRMS (ESI) *m*/*z*: [M + H]^+^ calcd for C_10_H_12_Cl_2_NO_2_ 248.0240, found 248.0229.

##### Ethyl (3,5-Dibromophenyl)glycinate (**1h**)

By following General Procedure B, **1h** (1.85 g, 55 mmol, 55%) was obtained as a white amorphous solid after column chromatography on silica gel (8.5:1.5 cyclohexane/EtOAc). ^1^H NMR (300 MHz, CDCl_3_): δ 7.01 (t, *J* = 1.5 Hz, 1H), 6.66 (d, *J* = 1.5 Hz, 2H), 4.45 (s, 1H), 4.26 (q, *J* = 7.1 Hz, 2H), 3.85 (d, *J* = 4.8 Hz, 2H), 1.31 (t, *J* = 7.1, 6.6 Hz, 3H). ^13^C{^1^H} NMR (101 MHz, CDCl_3_): δ 170.1, 148.9, 123.5, 123.2, 114.4, 61.7, 45.2, 14.2. HRMS (ESI) *m*/*z*: [M + H]^+^ calcd for C_10_H_12_Br_2_NO_2_ 335.9229, found 335.9244.

##### Ethyl (3-Chlorophenyl)glycinate (**1i**)

By following General Procedure A, **1i** (1.20 g, 5.6 mmol, 56%) was obtained as a white amorphous solid after column chromatography on silica gel (8:2 cyclohexane/EtOAc). ^1^H NMR (300 MHz, CDCl_3_): δ 7.09 (t, *J* = 8.0 Hz, 1H), 6.74–6.68 (m, 1H), 6.57 (t, *J* = 2.0 Hz, 1H), 6.51–6.45 (m, 1H), 4.38 (s, 1H), 4.26 (q, *J* = 7.1 Hz, 2H), 3.87 (d, *J* = 5.4 Hz, 2H), 1.31 (t, *J* = 7.1 Hz, 3H). ^13^C{^1^H} NMR (101 MHz, CDCl_3_): δ 170.6, 148.1, 135.0, 130.2, 118.0, 112.6, 111.3, 61.5, 45.5, 14.2. HRMS (ESI) *m*/*z*: [M + H]^+^ calcd for C_10_H_13_ClNO_2_ 214.0629, found 214.0635.

##### Ethyl (2-Fluorophenyl)glycinate (**1j**)

By following General Procedure A, **1j** (1.43 g, 7.3 mmol, 73%) was obtained as a white amorphous solid after column chromatography on silica gel (8:2 cyclohexane/EtOAc). ^1^H NMR (300 MHz, CDCl_3_): δ 6.99 (t, *J* = 8.4 Hz, 2H), 6.72–6.63 (m, 1H), 6.59 (t, *J* = 8.4 Hz, 1H), 4.52 (s, 1H), 4.25 (q, *J* = 7.1 Hz, 2H), 3.93 (d, *J* = 5.6 Hz, 2H), 1.31 (t, *J* = 7.1, 3H). ^13^C{^1^H} NMR (101 MHz, CDCl_3_): δ 170.6, 124.5, 117.6, 117.6, 114.7, 114.5, 112.2, 61.4, 45.5, 14.1. ^19^F NMR (376 MHz, CDCl_3_): δ −127.19 to −127.50 (m). HRMS (ESI) *m*/*z*: [M + H]^+^ calcd for C_10_H_13_FNO_2_ 198.0925, found 198.0918.

#### Procedure for the Preparation of 1-Benzyl-1*H*-indole **2j**

In a two-neck round-bottom flask under an argon atmosphere was prepared a mixture of indole (10 mmol, 1 equiv), benzyl bromide (15 mmol, 1.5 equiv), and sodium hydride (12 mmol, 1.2 equiv) in anhydrous dimethylformamide (10 mL, 1 M) at 0 °C (ice). The mixture was warmed to room temperature and reacted for 24 h. After 24 h, a solution of a saturated NaHCO_3_ solution (15 mL) was added and the aqueous layer was extracted three times with diethyl ether (3 × 10 mL). The organic layer was dried with anhydrous Na_2_SO_4_, filtered, and concentrated on the rotary evaporator. The crude product was purified by flash chromatography to give the final product as an amorphous solid.

##### 1-Benzyl-1*H*-indole (**2j**)

By following the procedure described above, **2j** (1.29 g, 6.2 mmol, 62%) was obtained as a pale red oil after column chromatography on silica gel (9:1 cyclohexane/EtOAc). ^1^H NMR (300 MHz, CDCl_3_): δ 7.66 (d, *J* = 7.1 Hz, 1H), 7.35–7.22 (m, 5H), 7.19 (dd, *J* = 7.1, 1.3 Hz, 1H), 7.16–7.05 (m, 4H), 6.56 (dd, *J* = 3.1, 0.8 Hz, 1H), 5.34 (s, 2H). ^13^C{^1^H} NMR (101 MHz, CDCl_3_): δ 137.5, 136.3, 128.7, 128.2, 127.6, 126.7, 121.7, 121.0, 119.5, 109.7, 101.7, 50.1, 29.7. HRMS (ESI) *m*/*z*: [M + H]^+^ calcd for C_15_H_14_N 208.1121, found 208.1112.

#### Procedure for the Preparation of Ethyl 2-(1,3-Dioxoisoindolin-2-yl)acetate

Phthalamide (7 mmol, 1 equiv), ethylchloroacetate (14 mmol, 2 equiv), and K_2_CO_3_ (14 mmol, 2 equiv) in anhydrous dimethylformamide (6 mL, 1.2 M) were added in a sealed flask and stirred at 40 °C (oil bath) for 6 h. Then, 10 mL of H_2_O was added, and the solution was extracted with dichloromethane (3 × 10 mL). The organic layer was dried with anhydrous Na_2_SO_4_, filtered, and concentrated on the rotary evaporator affording ethyl 2-(1,3-dioxoisoindolin-2-yl)acetate as a white amorphous solid (1.6 g, 6.0 mmol, 85%). ^1^H NMR (400 MHz, CDCl_3_): δ 7.88 (dd, *J* = 5.4, 3.1 Hz, 2H), 7.74 (dd, *J* = 5.4, 3.0 Hz, 2H), 4.43 (s, 2H), 4.22 (q, *J* = 7.1 Hz, 2H), 1.28 (t, *J* = 7.1 Hz, 3H). ^13^C{^1^H} NMR (101 MHz, CDCl_3_): δ 167.5, 167.2, 134.2, 132.0, 123.6, 61.9, 38.9, 14.1. HRMS (ESI) *m*/*z*: [M + H]^+^ calcd for C_12_H_12_NO_4_ 234.0761, found 234.0770. Spectroscopic data are in accord with the literature.^76,77^

#### Procedure for the Preparation of 2-Phenyl-1,2,3,4-tetrahydroisoquinoline

To a Schlenk flask were added copper(I) iodide (0.25 mmol, 0.1 equiv) and potassium phosphate (5 mmol, 2 equiv). The flask was evacuated and backfilled with nitrogen. Then 2-propanol (5 mL, 0.75 M) was added, followed by iodobenzene (2.5 mmol, 1 equiv), ethylene glycol (5 mmol, 2 equiv), and 1,2,3,4-tetrahydroisoquinoline (3.75 mmol, 1.5 equiv). The reaction mixture was heated at 90 °C (oil bath), stirred for 24 h, and then cooled to room temperature. Diethyl ether (10 mL) and water (10 mL) were then added. The aqueous layer was extracted three times with diethyl ether (3 × 10 mL). The combined organic layers were washed with brine, dried with anhydrous Na_2_SO_4_, and concentrated with the rotary evaporator. The residue was purified by flash column chromatography affording 2-phenyl-1,2,3,4-tetrahydroisoquinoline as a pale red solid (100 mg, 0.5 mmol, 20%). ^1^H NMR (400 MHz, CDCl_3_): δ 7.29 (d, *J* = 8.5 Hz, 2H), 7.22–7.08 (m, 4H), 6.99 (d, *J* = 8.5 Hz, 2H), 6.83 (t, *J* = 7.2 Hz, 1H), 4.42 (s, 2H), 3.57 (t, *J* = 5.8 Hz, 2H), 2.99 (t, *J* = 5.8 Hz, 2H). ^13^C{^1^H} NMR (101 MHz, CDCl_3_): δ 150.5, 137.4, 134.8, 129.2, 128.5, 126.5, 126.3, 126.0, 118.7, 115.1, 50.7, 46.5, 29.1. HRMS (ESI) *m*/*z*: [M + H]^+^ calcd for C_15_H_16_N 210.1277, found 210.1267. Spectroscopic data are in accord with the literature.^77^

#### Procedure for the Preparation of Ethyl Butylglycinate

In a round-bottom flask, a solution of *N*-butylamine (10 mmol, 1 equiv) and ethyl glyoxalate (10.4 mmol, 1.04 equiv, 50% in toluene) in ethanol (20 mL, 0.5 M) was stirred at room temperature for 1 h. Then, a solution of NaBH_3_CN (12 mmol, 1.2 equiv) and glacial acetic acid (1.6 mmol, 0.16 equiv) in ethanol (5 mL) was added to the mixture, and the reaction was prolonged for 2 h. The solvent was removed with the rotary evaporator, and the residue obtained was treated with a 10% solution of saturated NaOH with NaCl (10 mL) and extracted with diethyl ether. The organic layer was dried with Na_2_SO_4_, and the solvent was removed with the rotary evaporator to give the final product (ethyl butylglycinate) as a pale yellow oil (1.13 g, 7 mmol, 70%). ^1^H NMR (400 MHz, CDCl_3_): δ 4.14 (q, *J* = 7.1 Hz, 2H), 3.33 (s, 2H), 2.54 (t, *J* = 7.0 Hz, 3H), 1.50 (br s, 1H), 1.44 (quint, *J* = 7.0 Hz, 2H), 1.34 (sext, *J* = 7.0 Hz, 2H), 1.22 (t, *J* = 7.0 Hz, 3H), 0.85 (t, *J* = 7.0 Hz, 3H). ^13^C{^1^H} NMR (101 MHz, CDCl_3_): δ 172.4, 60.5, 50.9, 49.1, 32.0, 20.2, 14.0, 13.8. HRMS (ESI) *m*/*z*: [M + H]^+^ calcd for C_8_H_18_NO_2_ 160.1332, found 160.1340. Spectroscopic data are in accord with the literature.^78^

#### Procedure for the Preparation of Ethyl (*E*)-2-(Phenylimino)acetate

In a two-neck round-bottom flask under an argon atmosphere was prepared a mixture of aniline (10 mmol, 1 equiv), ethyl glyoxalate (10 mmol, 1 equiv, 50% in toluene), and anhydrous Na_2_SO_4_ (17 mmol, 1.7 equiv) in toluene (40 mL, 0.25 M). The mixture was refluxed at 110 °C (oil bath) for 1 h to afford the C-acylimine (**IV**). The crude was then filtered, and the solvent was removed with the rotary evaporator, affording ethyl (*E*)-2-(phenylimino)acetate (1.59 g, 9 mmol, 90%) as a yellow amorphous solid that was directly used without further purification. ^1^H NMR (300 MHz, CDCl_3_): δ 7.92 (s, 1H), 7.44–7.30 (m, 5H), 7.18 (d, *J* = 6.3 Hz, 1H), 4.43 (q, *J* = 7.1 Hz, 2H), 1.42 (t, *J* = 7.1 Hz, 3H). ^13^C{^1^H} NMR (101 MHz, CDCl_3_): δ 153.68 (s), 151.24 (s), 129.51 (s), 129.31 (s), 121.63 (s), 121.39 (s), 62.11 (s), 14.19 (s). HRMS (ESI) *m*/*z*: [M + H]^+^ calcd for C_10_H_12_NO_2_ 170.0863, found 170.0870. Spectroscopic data are in accord with the literature.^79^

#### General Procedure A: Light-Driven CDC between Aryl Glycine and Indoles Mediated by mpg-CN

In a microwave vial in anhydrous ethyl acetate (1 mL, 0.1 M) were placed phenyl glycine (0.13 mmol, 1.3 equiv), indole (0.1 mmol, 1 equiv), and mesoporous graphitic carbon nitride (mpg-CN) (10 mg) in an air atmosphere. The suspension was stirred for 16 h and irradiated with blue LED light (465 nm, 20 W) at a distance of 5 cm. At the end of the process, the reaction mixture was filtered on cotton and silica, which were subsequently washed three times to remove some of the products, and reagents were left to soak in the pores of the photocatalyst. The mixture was concentrated on the rotary evaporator in a Schlenk line to remove the residual solvent. The crude product was purified by flash chromatography to give the final product as an amorphous solid.

#### General Procedure B: Light-Driven CDC between Aryl Glycine and Indoles Mediated by mpg-CN

In a microwave vial in anhydrous ethyl acetate (1 mL, 0.1 M) were placed phenyl glycine (0.1 mmol, 1 equiv), indole (0.13 mmol, 1.3 equiv), and mesoporous graphitic carbon nitride (mpg-CN) (10 mg) in an air atmosphere. The suspension was stirred for 16 h and irradiated with blue LED light (456 nm, 20 W) at a distance of 5 cm. At the end of the process, the reaction mixture was filtered on cotton and silica, which were subsequently washed three times to remove some of products, and reagents were left to soak in the pores of the photocatalyst. The mixture was concentrated on the rotary evaporator in a Schlenk line to remove the residual solvent. The crude product was purified by flash chromatography to give the final product as an amorphous solid.

##### Ethyl 2-(1*H*-Indol-3-yl)-2-(phenylamino)acetate (**3aa**)

By following General Procedure A, **3aa** (21 mg, 0.071 mmol, 71%, gram scale, 5.6 mmol of **1a** employed; 872 mg, 3.0 mmol, 69%) was obtained as a white amorphous solid after column chromatography on silica gel (8:2 cyclohexane/EtOAc). ^1^H NMR (300 MHz, CDCl_3_): δ 8.13 (s, 1H), 7.84 (d, *J* = 8.0 Hz, 1H), 7.39 (d, *J* = 8.0 Hz, 1H), 7.25–7.11 (m, 5H), 6.72 (t, *J* = 7.5 Hz, 1H), 6.64 (d, *J* = 7.5 Hz, 2H), 5.40 (d, *J* = 5.5 Hz, 1H), 4.77 (s, 1H), 4.32–4.06 (m, 2H), 1.22 (t, *J* = 7.1 Hz, 3H). ^13^C{^1^H} NMR (101 MHz, CDCl_3_): δ 172.5, 146.5, 136.5, 129.2, 125.8, 123.0, 122.5, 120.0, 119.6, 118.0, 113.3, 112.7, 111.3, 61.6, 54.2, 14.1. HRMS (ESI) *m*/*z*: [M + H]^+^ calcd for C_18_H_19_N_2_O_2_ 295.1441, found 295.1429.

##### Ethyl 2-(1*H*-Indol-3-yl)-2-(*p*-tolylamino)acetate (**3ba**)

By following General Procedure A, **3ba** (23 mg, 0.075 mmol, 75%) was obtained as a white amorphous solid after column chromatography on silica gel (8:2 cyclohexane/EtOAc). ^1^H NMR (300 MHz, acetone-*d*_6_): δ 10.29 (s, 1H), 7.78 (d, *J* = 7.9 Hz, 1H), 7.41 (dd, *J* = 6.0, 1.8 Hz, 2H), 7.20–6.98 (m, 2H), 6.92 (d, *J* = 8.1 Hz, 2H), 6.73–6.61 (m, 2H), 5.41 (d, *J* = 7.6 Hz, 1H), 5.24 (d, *J* = 7.6 Hz, 1H), 4.25–3.99 (m, 2H), 2.16 (s, 3H), 1.16 (t, *J* = 7.1 Hz, 3H). ^13^C{^1^H} NMR (101 MHz, acetone-*d*_6_): δ 172.5, 145.4, 136.9, 129.4, 126.2, 126.0, 123.9, 121.8, 119.4, 119.2, 113.4, 112.0, 111.6, 60.6, 54.3, 19.6, 13.7. HRMS (ESI) *m*/*z*: [M + H]^+^ calcd for C_19_H_21_N_2_O_2_ 309.1598, found 309.1610.

##### Ethyl 2-(1*H*-Indol-3-yl)-2-[(4-methoxyphenyl)amino]acetate (**3ca**)

By following General Procedure A, **3ca** (24 mg, 0.073 mmol, 73%) was obtained as a white amorphous solid after column chromatography on silica gel (8:2 cyclohexane/EtOAc). ^1^H NMR (300 MHz, CDCl_3_): δ 8.13 (s, 1H), 7.83 (d, *J* = 7.8 Hz, 1H), 7.38 (d, *J* = 7.7 Hz, 1H), 7.28–7.22 (m, 3H), 7.22–7.11 (m, 1H), 6.80–6.68 (m, 2H), 6.66–6.56 (m, 2H), 5.33 (s, 1H), 4.32–4.06 (m, 2H), 3.72 (s, 3H), 1.22 (t, *J* = 7.1 Hz, 3H). ^13^C{^1^H} NMR (101 MHz, CDCl_3_): δ 172.7, 152.5, 140.8, 136.4, 131.6, 125.8, 123.0, 122.5, 120.0, 119.6, 114.8, 112.9, 111.3, 61.5, 55.7, 55.2, 14.1. HRMS (ESI) *m*/*z*: [M + H]^+^ calcd for C_19_H_21_N_2_O_3_ 325.1547, found 325.1558.

##### Ethyl 2-[(4-Fluorophenyl)amino]-2-(1*H*-indol-3-yl)acetate (**3da**)

By following General Procedure A, **3da** (25 mg, 0.079 mmol, 79%) was obtained as a white amorphous solid after column chromatography on silica gel (8:2 cyclohexane/EtOAc). ^1^H NMR (400 MHz, CDCl_3_): δ 8.15 (s, 1H), 7.82 (d, *J* = 8.0 Hz, 1H), 7.39 (d, *J* = 7.2 Hz, 1H), 7.25–7.13 (m, 3H), 6.85 (t, *J* = 8.9 Hz, 2H), 6.60–6.57 (dd, *J* = 8.9, 4.4 Hz, 2H), 5.33 (s, 1H), 4.65 (s, 1H), 4.30–4.05 (m, 2H), 1.22 (t, *J* = 7.1 Hz, 3H). ^13^C{^1^H} NMR (101 MHz, CDCl_3_): δ 172.4, 136.5, 125.8, 123.0, 122.6, 120.1, 119.5, 115.8, 115.5, 114.3, 114.3, 112.6, 111.4, 61.6, 54.8, 14.1. ^19^F NMR (376 MHz, CDCl_3_): δ −127.25 to −127.34 (m). HRMS (ESI) *m*/*z*: [M + H]^+^ calcd for C_18_H_18_FN_2_O_2_ 313.1347, found 313.1335.

##### Ethyl 2-[(4-Chlorophenyl)amino]-2-(1*H*-indol-3-yl)acetate (**3ea**)

By following General Procedure A, **3ea** (26 mg, 0.078 mmol, 78%) was obtained as a white amorphous solid after column chromatography on silica gel (8:2 cyclohexane/EtOAc). ^1^H NMR (400 MHz, acetone-*d*_6_): δ 10.33 (s, 1H), 7.79 (d, *J* = 9.1 Hz, 1H), 7.50–7.36 (m, 2H), 7.17–7.05 (m, 4H), 6.79 (d, *J* = 9.1 Hz, 2H), 5.68 (d, *J* = 7.2 Hz, 1H), 5.45 (d, *J* = 7.2 Hz, 1H), 4.28–4.01 (m, 2H), 1.18 (t, *J* = 7.1 Hz, 3H). ^13^C{^1^H} NMR (101 MHz, acetone-*d*_6_): δ 172.0, 146.5, 128.7, 126.1, 124.0, 123.9, 121.8, 121.1, 119.3, 119.3, 114.5, 111.6, 111.6, 60.8, 54.1, 13.6. HRMS (ESI) *m*/*z*: [M + H]^+^ calcd for C_18_H_18_ClN_2_O_2_ 329.1051, found 329.1042.

##### Ethyl 2-[(4-Bromophenyl)amino]-2-(1*H*-indol-3-yl)acetate (**3fa**)

By following General Procedure A, **3fa** (24 mg, 0.065 mmol, 65%) was obtained as a white amorphous solid after column chromatography on silica gel (8:2 cyclohexane/EtOAc). ^1^H NMR (300 MHz, CDCl_3_): δ 8.14 (s, 1H), 7.81 (d, *J* = 7.8 Hz, 1H), 7.39 (d, *J* = 9.1 Hz, 1H), 7.23–7.15 (m, 5H), 6.50 (d, *J* = 8.9 Hz, 2H), 5.34 (d, *J* = 6.3 Hz, 1H), 4.83 (d, *J* = 5.8 Hz, 1H), 4.32–4.02 (m, 2H), 1.22 (t, *J* = 6.8 Hz, 3H). ^13^C{^1^H} NMR (101 MHz, CDCl_3_): δ 172.1, 145.4, 136.5, 131.9, 125.7, 123.0, 122.6, 120.1, 119.5, 114.9, 112.2, 111.4, 109.7, 61.7, 54.1, 14.1. HRMS (ESI) *m*/*z*: [M + H]^+^ calcd for C_18_H_18_BrN_2_O_2_ 373.0546, found 373.0559.

##### Ethyl 2-[(3-Chlorophenyl)amino]-2-(1*H*-indol-3-yl)acetate (**3ia**)

By following General Procedure A, **3ia** (19 mg, 0.058 mmol, 58%) was obtained as a white amorphous solid after column chromatography on silica gel (8:2 cyclohexane/EtOAc). ^1^H NMR (300 MHz, CDCl_3_): δ 8.16 (s, 1H), 7.81 (d, *J* = 7.9 Hz, 1H), 7.39 (d, *J* = 7.9 Hz, 1H), 7.26–7.13 (m, 3H), 7.04 (t, *J* = 7.9 Hz, 1H), 6.70–6.60 (m, 1H), 6.62 (t, *J* = 2.1 Hz, 1H), 6.51–6.47 (m, 1H), 5.36 (s, 1H), 4.87 (s, 1H), 4.31–4.11 (m, 2H), 1.22 (t, *J* = 7.1 Hz, 3H). ^13^C{^1^H} NMR (101 MHz, CDCl_3_): δ 172.1, 147.6, 136.5, 134.9, 130.2, 125.7, 123.1, 122.7, 120.1, 119.5, 117.9, 113.1, 112.2, 111.5, 111.4, 61.7, 54.0, 14.1. HRMS (ESI) *m*/*z*: [M + H]^+^ calcd for C_18_H_18_ClN_2_O_2_ 329.1051, found 329.1037.

##### Ethyl 2-[(2-Fluorophenyl)amino]-2-(1*H*-indol-3-yl)acetate (**3ja**)

By following General Procedure A, **3ja** (19 mg, 0.062 mmol, 62%) was obtained as a white amorphous solid after column chromatography on silica gel (8:2 cyclohexane/EtOAc). ^1^H NMR (300 MHz, CDCl_3_): δ 8.15 (s, 1H), 7.84 (d, *J* = 7.9 Hz, 1H), 7.39 (d, *J* = 7.9 Hz, 1H), 7.29–7.26 (m, 1H), 7.22–7.15 (m, 2H), 7.02–6.94 (m, 1H), 6.90 (t, *J* = 7.9 Hz, 1H), 6.68–6.58 (m, 2H), 5.41 (d, *J* = 6.4 Hz, 1H), 5.03 (s, 1H), 4.33–4.08 (m, 2H), 1.22 (t, *J* = 7.1 Hz, 3H). ^13^C{^1^H} NMR (101 MHz, CDCl_3_): δ 172.0, 136.5, 125.7, 124.4, 123.0, 122.6, 120.1, 119.5, 117.5, 117.4, 114.7, 114.5, 112.9, 112.4, 111.3, 61.6, 54.0, 14.1. ^19^F NMR (376 MHz, CDCl_3_): δ −135.45 to −135.56 (m). HRMS (ESI) *m*/*z*: [M + H]^+^ calcd for C_18_H_18_FN_2_O_2_ 313.1347, found 313.1354.

##### Ethyl 2-(2-Methyl-1*H*-indol-3-yl)-2-(phenylamino)acetate (**3ab**)

By following General Procedure B, **3ab** (20 mg, 0.065 mmol, 65%) was obtained as a white amorphous solid after column chromatography on silica gel (8:2 cyclohexane/EtOAc). ^1^H NMR (300 MHz, CDCl_3_): δ 7.89 (s, 1H), 7.78 (d, *J* = 8.3 Hz, 1H), 7.19–7.02 (m, 5H), 6.69 (t, *J* = 7.4 Hz, 1H), 6.60 (d, *J* = 7.4 Hz, 2H), 5.28 (s, 1H), 4.78 (s, 1H), 4.32–3.97 (m, 2H), 2.51 (s, 3H), 1.18 (t, *J* = 7.1 Hz, 3H). ^13^C{^1^H} NMR (101 MHz, CDCl_3_): δ 172.4, 146.7, 135.2, 133.2, 129.2, 126.9, 121.5, 120.0, 119.0, 117.9, 113.2, 110.4, 107.8, 61.5, 54.1, 14.2, 12.3. HRMS (ESI) *m*/*z*: [M + H]^+^ calcd for C_19_H_21_N_2_O_2_ 309.1598, found 309.1585.

##### Ethyl 2-(6-Methyl-1*H*-indol-3-yl)-2-(phenylamino)acetate (**3ac**)

By following General Procedure B, **3ac** (17 mg, 0.056 mmol, 56%) was obtained as a white amorphous solid after column chromatography on silica gel (8:2 cyclohexane/EtOAc). ^1^H NMR (300 MHz, CDCl_3_): δ 8.00 (s, 1H), 7.71 (d, *J* = 8.2 Hz, 1H), 7.17 (d, *J* = 2.5 Hz, 2H), 7.13 (d, *J* = 8.2 Hz, 2H), 7.01 (d, *J* = 8.2 Hz, 1H), 6.71 (t, *J* = 7.4 Hz, 1H), 6.63 (d, *J* = 7.4 Hz, 2H), 5.35 (s, 1H), 4.75 (s, 1H), 4.34–4.04 (m, 2H), 2.47 (s, 3H), 1.22 (t, *J* = 7.1 Hz, 3H). ^13^C{^1^H} NMR (75 MHz, CDCl_3_): δ 172.9, 146.9, 137.3, 132.8, 129.5, 124.0, 122.7, 122.2, 119.6, 118.3, 113.7, 112.9, 111.6, 61.9, 54.7, 22.0, 14.5. HRMS (ESI) *m*/*z*: [M + H]^+^ calcd for C_19_H_21_N_2_O_2_ 309.1598, found 309.1609.

##### Ethyl 2-(6-Methoxy-1*H*-indol-3-yl)-2-(phenylamino)acetate (**3ad**)

By following General Procedure B, **3ad** (19 mg, 0.058 mmol, 58%) was obtained as a white amorphous solid after column chromatography on silica gel (8:2 cyclohexane/EtOAc). ^1^H NMR (300 MHz, CDCl_3_): δ 7.98 (s, 1H), 7.70 (d, *J* = 8.5 Hz, 1H), 7.17–7.10 (m, 3H), 6.88–6.79 (m, 2H), 6.71 (t, *J* = 7.4 Hz, 1H), 6.63 (d, *J* = 7.4 Hz, 2H), 5.34 (d, *J* = 6.2 Hz, 1H), 4.75 (d, *J* = 6.2 Hz, 1H), 4.33–4.05 (m, 2H), 3.85 (s, 3H), 1.22 (t, *J* = 7.1 Hz, 3H). ^13^C{^1^H} NMR (101 MHz, CDCl_3_): δ 172.5, 156.8, 146.5, 137.3, 129.2, 121.8, 120.3, 120.1, 118.0, 113.3, 112.7, 110.1, 94.7, 61.5, 55.6, 54.3, 14.1. HRMS (ESI) *m*/*z*: [M + H]^+^ calcd for C_19_H_21_N_2_O_3_ 325.1547, found 325.1539.

##### Ethyl 2-(6-Fluoro-1*H*-indol-3-yl)-2-(phenylamino)acetate (**3ae**)

By following General Procedure A, **3ae** (25 mg, 0.081 mmol, 81%) was obtained as a white amorphous solid after column chromatography on silica gel (8:2 cyclohexane/EtOAc). ^1^H NMR (300 MHz, CDCl_3_): δ 8.11 (s, 1H), 7.75 (dd, *J* = 8.7, 5.3 Hz, 1H), 7.22 (d, *J* = 2.5 Hz, 1H), 7.14 (t, *J* = 7.6 Hz, 2H), 7.06 (dd, *J* = 9.5, 2.5 Hz, 1H), 6.97–6.87 (m, 1H), 6.72 (t, *J* = 7.3 Hz, 1H), 6.63 (d, *J* = 7.6 Hz, 2H), 5.35 (d, *J* = 4.7 Hz, 1H), 4.76 (d, *J* = 4.1 Hz, 1H), 4.32–4.02 (m, 2H), 1.22 (t, *J* = 7.1 Hz, 3H). ^13^C{^1^H} NMR (75 MHz, CDCl_3_): δ 172.6, 146.7, 129.6, 123.6, 120.9, 120.8, 118.5, 113.7, 113.3, 109.4, 109.1, 98.2, 97.8, 62.0, 54.6, 14.5. ^19^F NMR (376 MHz, CDCl_3_): δ −120.37 (td, *J* = 9.5, 5.3 Hz). HRMS (ESI) *m*/*z*: [M + H]^+^ calcd for C_18_H_18_FN_2_O_2_ 313.1347, found 313.1338.

##### Ethyl 2-(6-Chloro-1*H*-indol-3-yl)-2-(phenylamino)acetate (**3af**)

By following General Procedure A, **3af** (23 mg, 0.07 mmol, 70%) was obtained as a white amorphous solid after column chromatography on silica gel (8:2 cyclohexane/EtOAc). ^1^H NMR (300 MHz, CDCl_3_): δ 8.11 (s, 1H), 7.75 (d, *J* = 8.5 Hz, 1H), 7.37 (d, *J* = 1.7 Hz, 1H), 7.24 (d, *J* = 2.6 Hz, 1H), 7.18–7.10 (m, 3H), 6.72 (t, *J* = 7.4 Hz, 1H), 6.62 (d, *J* = 7.4 Hz, 2H), 5.35 (d, *J* = 5.7 Hz, 1H), 4.78 (d, *J* = 5.7 Hz, 1H), 4.32–4.05 (m, 2H), 1.21 (t, *J* = 7.1 Hz, 3H). ^13^C{^1^H} NMR (101 MHz, CDCl_3_): δ 172.3, 146.4, 136.9, 129.3, 124.5, 123.7, 123.1, 120.9, 120.7, 118.3, 113.5, 113.2, 111.3, 61.8, 54.2, 14.2. HRMS (ESI) *m*/*z*: [M + H]^+^ calcd for C_18_H_18_ClN_2_O_2_ 329.1051, found 329.1063.

##### Ethyl 2-(6-Bromo-1*H*-indol-3-yl)-2-(phenylamino)acetate (**3ag**)

By following General Procedure A, **3ag** (23 mg,0.062 mmol, 62%) was obtained as a white amorphous solid after column chromatography on silica gel (8:2 cyclohexane/EtOAc). ^1^H NMR (300 MHz, CDCl_3_): δ 8.12 (s, 1H), 7.70 (d, *J* = 8.5 Hz, 1H), 7.54 (d, *J* = 1.7 Hz, 1H), 7.28 (d, *J* = 1.7 Hz, 1H), 7.23 (d, *J* = 2.6 Hz, 1H), 7.14 (t, *J* = 7.8 Hz, 2H), 6.72 (t, *J* = 7.4 Hz, 1H), 6.61 (d, *J* = 7.4 Hz, 2H), 5.35 (s, 1H), 4.79 (s, 1H), 4.31–4.04 (m, 2H), 1.21 (t, *J* = 7.1 Hz, 3H). ^13^C{^1^H} NMR (101 MHz, CDCl_3_): δ 172.2, 146.4, 137.4, 129.3, 124.8, 123.7, 123.5, 121.0, 118.3, 116.2, 114.3, 113.5, 113.2, 61.8, 54.2, 14.2. HRMS (ESI) *m*/*z*: [M + H]^+^ calcd for C_18_H_18_BrN_2_O_2_ 373.0546, found 373.0560.

##### Ethyl 2-(1-Methyl-1*H*-indol-3-yl)-2-(phenylamino)acetate (**3ah**)

By following General Procedure B, **3ah** (20 mg, 0.064 mmol, 64%) was obtained as a white amorphous solid after column chromatography on silica gel (8:2 cyclohexane/EtOAc). ^1^H NMR (300 MHz, CDCl_3_): δ 7.82 (d, *J* = 7.9 Hz, 1H), 7.31 (t, *J* = 6.8 Hz, 2H), 7.20–7.09 (m, 4H), 6.72 (t, *J* = 7.7 Hz, 1H), 6.64 (d, *J* = 7.7 Hz, 2H), 5.37 (d, *J* = 6.1 Hz 1H), 4.74 (d, *J* = 6.1 Hz, 1H), 4.36–4.03 (m, 2H), 3.75 (s, 3H), 1.22 (t, *J* = 7.1 Hz, 3H). ^13^C{^1^H} NMR (101 MHz, CDCl_3_): δ 172.6, 146.5, 129.2, 127.6, 126.3, 122.0, 119.6, 119.5, 117.9, 113.3, 110.9, 109.5, 61.5, 54.2, 32.9, 14.1. HRMS (ESI) *m*/*z*: [M + H]^+^ calcd for C_19_H_21_N_2_O_2_ 309.1598, found 309.1586.

##### Ethyl 2-(1-Benzyl-1*H*-indol-3-yl)-2-(phenylamino)acetate (**3aj**)

By following General Procedure A, **3aj** (28 mg, 0.073 mmol, 73%) was obtained as a white amorphous solid after column chromatography on silica gel (8:2 cyclohexane/EtOAc). ^1^H NMR (300 MHz, CDCl_3_): δ 7.84 (d, *J* = 6.9 Hz, 1H), 7.27 (d, *J* = 6.9 Hz, 4H), 7.22–7.10 (m, 5H), 7.07 (dd, *J* = 6.9, 2.2 Hz, 2H), 6.72 (t, *J* = 7.4 Hz, 1H), 6.65 (d, *J* = 7.4 Hz, 2H), 5.39 (d, *J* = 6.2 Hz, 1H), 5.27 (s, 2H), 4.74 (d, *J* = 6.2 Hz, 1H), 4.33–4.05 (m, 2H), 1.21 (t, *J* = 7.1 Hz, 3H). ^13^C{^1^H} NMR (101 MHz, CDCl_3_): δ 172.0, 146.0, 136.4, 136.3, 128.6, 128.2, 127.1, 126.6, 126.2, 126.0, 121.7, 119.2, 119.2, 117.5, 112.8, 111.0, 109.4, 60.9, 53.7, 49.6, 13.5. HRMS (ESI) *m*/*z*: [M + H]^+^ calcd for C_25_H_25_N_2_O_2_ 385.1911, found 385.1925.

### Mechanistic Experiments

#### H_2_O_2_ Detection Experiment

For the qualitative determination of hydrogen peroxide in the reaction, we followed the detection procedure proposed by Pelit. FeCl_3_·6H_2_O (0.8 mmol, 0.209 g) was dissolved in 10 mL of ultrapure water along with Na_2_H_2_ EDTA (15.0 mmol, 5.2 g), and the mixture was stirred until complete dissolution of the solid phase. Then, 10 mL of NH_3_ [25% (w/w) solution in water] was added to the solution mentioned above. Then, the benchmark reaction mixture was prepared. In a microwave vial, ultrapure water (1 mL, 0.1 M), phenyl glycine (0.13 mmol, 1.3 equiv), indole (0.1 mmol, 1 equiv), and mesoporous graphitic carbon nitride (mpg-CN) (10 mg) were added and kept in an air atmosphere (balloon). The suspension was stirred for 3 h, and then 200 μL of Fe(III)-EDTA was added. The test gave a negative response (the solution did not turn purple): hydrogen peroxide was not detected.

#### Imine-Intermediate Mechanistic Experiment

Ethyl (*E*)-2-(phenylimino)acetate (0.13 mmol, 1.3 equiv), indole (0.1 mmol, 1 equiv), mesoporous graphitic carbon nitride (mpg-CN) (10 mg), and anhydrous ethyl acetate (1 mL, 0.1 M) were placed in a microwave vial kept in an air atmosphere (balloon). The suspension was stirred for 8 h and irradiated with blue LED light (465 nm, 20 W) at a distance of 5 cm. At the end of the process, the reaction mixture was filtered on silica, which was subsequently washed three times to remove the part of products and reagents left. The mixture was concentrated on the rotary evaporator, and the crude product was purified by flash chromatography (see **3aa** purification in the general procedure part) to give the final product as an amorphous solid (23 mg, 0.08 mmol, 80%).

## Supporting Information Available

Additional screening tests and characterization data for mpg-CN and compounds (PDF)
FAIR data, including the primary NMR FID files, for compounds **1a–1j**, **2j**, **3aa–3ja**, and **4–6** (ZIP)
