# Supplementary material for: Photoredox Cross-Dehydrogenative Coupling of N-Aryl Glycines Mediated by Mesoporous Graphitic Carbon Nitride: An Environmentally Friendly Approach to the Synthesis of Non-Proteinogenic α-Amino Acids (NPAAs) Decorated with Indoles
Source: J Org Chem. 2022 May 27;87(12):7826–37. doi: 10.1021/acs.joc.2c00474 (PMC9207928; doi:10.1021/acs.joc.2c00474)
Supplement: Supplementary file 1 — jo2c00474_si_001.pdf [file jo2c00474_si_001.pdf]

Photoredox Cross Dehydrogenative Coupling of *N*-Aryl-Glycines Mediated by Mesoporous Graphitic-Carbon Nitride: an environmental-friendly approach to the synthesis of non-proteinogenic α-amino acids (NPAAs) decorated with indoles

Lorenzo Poletti,[a] Daniele Ragno,[a] Olga Bortolini,[b] Francesco Presini,[a] Fabio Pesciaioli,[c] Stefano Carli,[b] Stefano Caramori,[a] Alessandra Molinari,[a] Alessandro Massi[a] and Graziano Di Carmine[a,*]

Corresponding authors

Graziano Di Carmine - [a]Department of Chemical, Pharmaceutical and Agricultural Sciences, University of Ferrara, Via L. Borsari, 46, 44121 Ferrara (Italy); Email: graziano.dicarmine@unife.it

Authors:

Lorenzo Poletti: [a]Department of Chemical, Pharmaceutical and Agricultural Sciences, University of Ferrara, Via L. Borsari, 46, 44121 Ferrara (Italy)

Daniele Ragno: [a]Department of Chemical, Pharmaceutical and Agricultural Sciences, University of Ferrara, Via L. Borsari, 46, 44121 Ferrara (Italy)

Olga Bortolini: [b]Department of Environmental and Prevention Sciences, University of Ferrara, Via L. Borsari, 46, 44121 Ferrara (Italy)

Francesco Presini: [a]Department of Chemical, Pharmaceutical and Agricultural Sciences, University of Ferrara, Via L. Borsari, 46, 44121 Ferrara (Italy)

Fabio Pesciaioli: [c]Department of Physical and Chemical Sciences, Università degli Studi dell'Aquila, Via Vetoio, 42, 67100, L'Aquila (Italy)

Stefano Carli: [b]Department of Environmental and Prevention Sciences, University of Ferrara, Via L. Borsari, 46, 44121 Ferrara (Italy)

Stefano Caramori: [a]Department of Chemical, Pharmaceutical and Agricultural Sciences, University of Ferrara, Via L. Borsari, 46, 44121 Ferrara (Italy)

Alessandra Molinari: [a]Department of Chemical, Pharmaceutical and Agricultural Sciences, University of Ferrara, Via L. Borsari, 46, 44121 Ferrara (Italy)

Alessandro Massi: [a]Department of Chemical, Pharmaceutical and Agricultural Sciences, University of Ferrara, Via L. Borsari, 46, 44121 Ferrara (Italy)

Supporting Information

SUMMARY

PHOTO-ELECTROCHEMICAL CHARACTERIZATION mpg-CN S3

ELECTRON MICROSCOPE CHARACTERIZATION S6

TABLES S9

- **Table S1. Preliminary tests** S9
- **Table S2. Variation of light power and tests without additive** S10
- **Table S3. Effects of light, temperature and catalyst** S11
- **Table S4. Solvents screening** S12
- **Table S5. Effects of light power** S13
- **Table S6. Effects of catalytic loading** S14

BY-PRODUCTS IDENTIFICATION S15

- **PEAK LIST OF BY-PRODUCTS** S16
- **UNSUCCESSFUL SCOPE COMPOUNDS** S17
- **NMR OF BY-PRODUCTS** S18

NMR OF STARTING COMPOUNDS S21

NMR OF PRODUCTS S34

References S53

PHOTO-ELECTROCHEMICAL CHARACTERIZATION mpg-CN

Absorption and photoluminescence spectra of mpg-CNs were recorded on powder disperse in quartz cuvette showing the typical absorption and emission peaks centered at ~ 360 nm and ~ 510 nm, respectively, which are linked to the π-π* transitions. From the interception of the absorption and emission spectra, an optical semiconductor bandgap of 2.9 eV was calculated (figure S1). According to the Kubelka–Munk function, the measured diffuse reflectance can be converted to the corresponding absorption spectra

$$F\,(R_\infty) = \frac{K}{S} = \frac{(1-R_\infty)^2}{2R_\infty} \quad \textbf{(1)}$$

where K and S are the absorption and scattering coefficients, respectively, and $R_\infty = \frac{R_{sample}}{R_{standard}}$ is the reflectance of an infinitely thick film.[1]

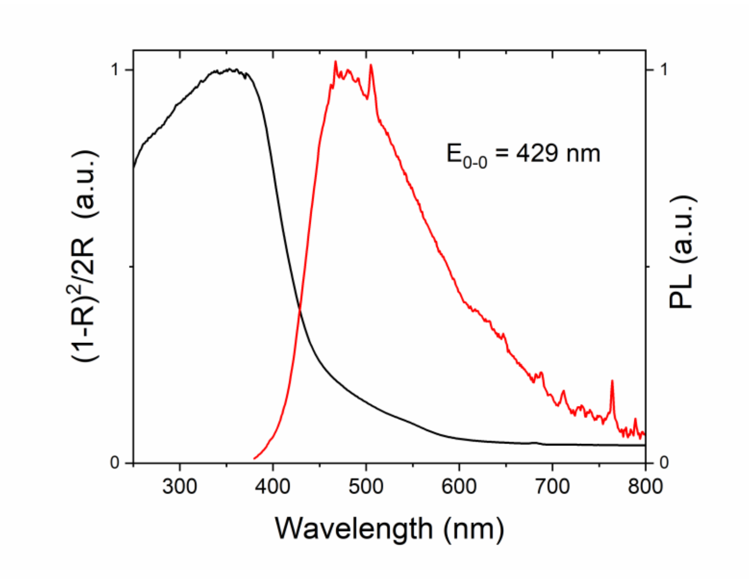

Figure S1. absorption and emission spectra of CNs powder in quartz cuvette.

The same optical bandgap value was extracted according to the Tauc plot, following the equation

$$(\alpha \cdot h\nu)^{1/\gamma} = B(h\nu - E_g) \quad \textbf{(2)}$$

where α is the energy-dependent absorption coefficient, h is the Planck constant, ν is the photon's frequency, E_g is the band gap energy, and B is a constant. The γ factor can be equal to 1/2 or 2 for direct or indirect transition band gaps, respectively.[2]

Thus, replacing α in equation (2) with $F\,(R_\infty)$ of (**1**) one can obtain

$$(F\,(R_\infty) \cdot h\nu)^{1/\gamma} = B(h\nu - E_g) \quad \textbf{(3)}$$

which relates the measured diffuse reflectance to the semiconductor optical bandgap. As reported in **Figure S2**, the linear part of the Tauc plot was fitted according to equation (**3**), which provided a γ

parameter value of 2, thereby indicating an indirect transition band. The optical energy gap of 2.9 eV can be obtained extrapolating this linear part to the x-axis.

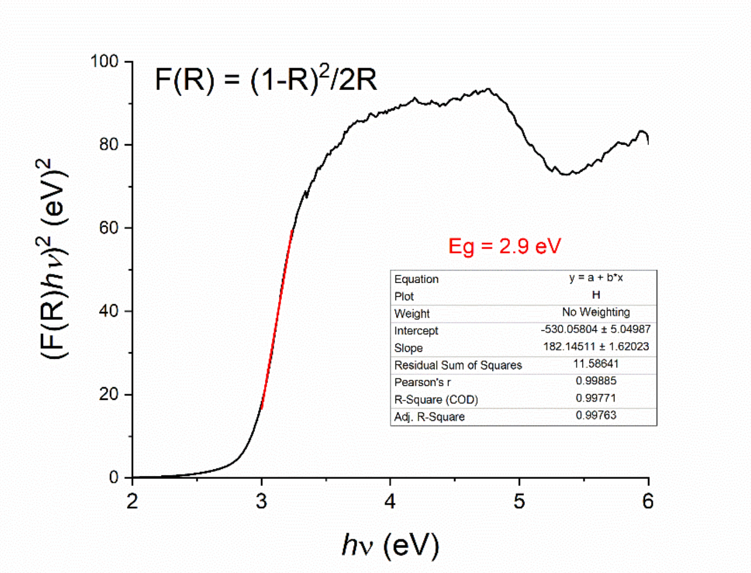

Figure S2. linear part of the Tauc plot

Cyclic voltammetry of mpg-CNs film on FTO glass is reported in **Figure S3**; it shows the absence of any significant anodic feature up to +2.0 V (vs SCE). The semiconductor's conduction band can be estimated at -0.74 V (vs SCE), which corresponds to the onset of the cathodic signal.

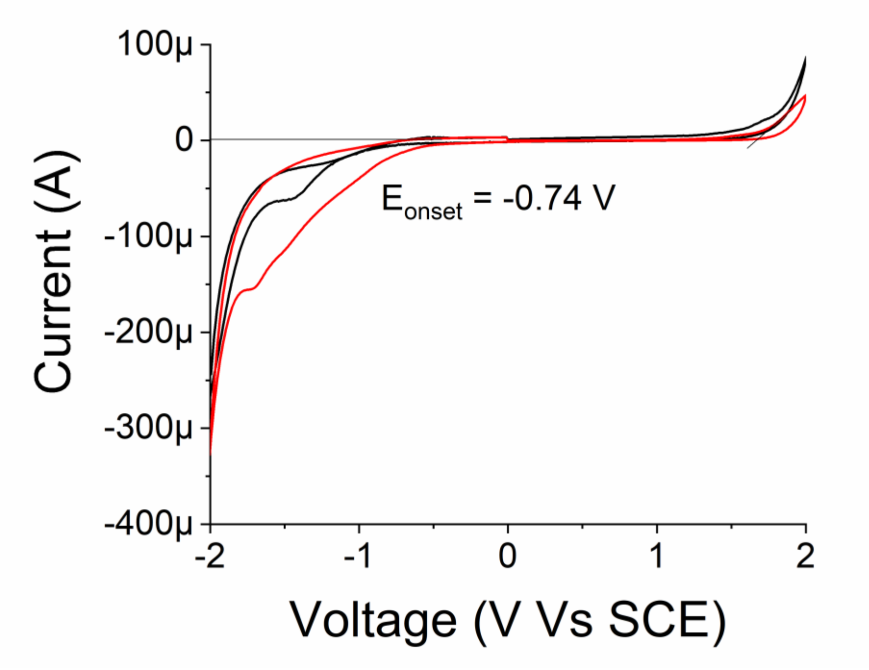

Figure S3. Cyclic voltammetry of CNs on FTO in deoxygenated 0.1 N $LiClO_4$/ACN.

Finally, cyclic voltammetry analysis of **1a** (ethyl 2-(phenylamino)acetate) was mainly dominated by the irreversible oxidation at E_{ox} = 1.2 (vs SCE), which match with the oxidation potential of photo-excited mpg-CN, which was estimated at ≈ 2.1 V (Vs SCE) according to $E_g = E_{VB} – E_{CB}$.

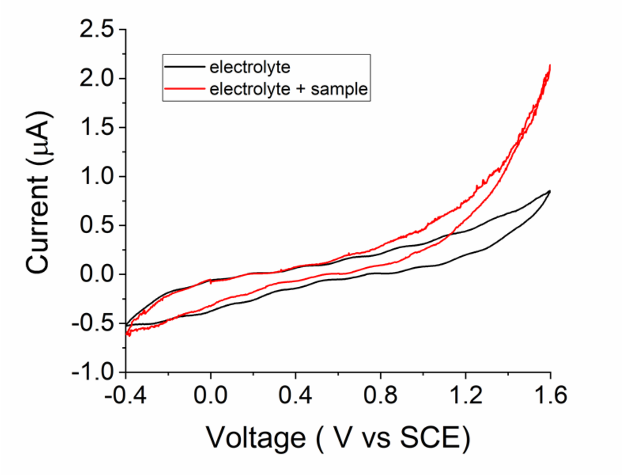

Figure S4. CV analysis of phenyl-glycine in 0.1 N $LiClO_4$/ACN.

ELECTRON MICROSCOPE CHARACTERIZATION

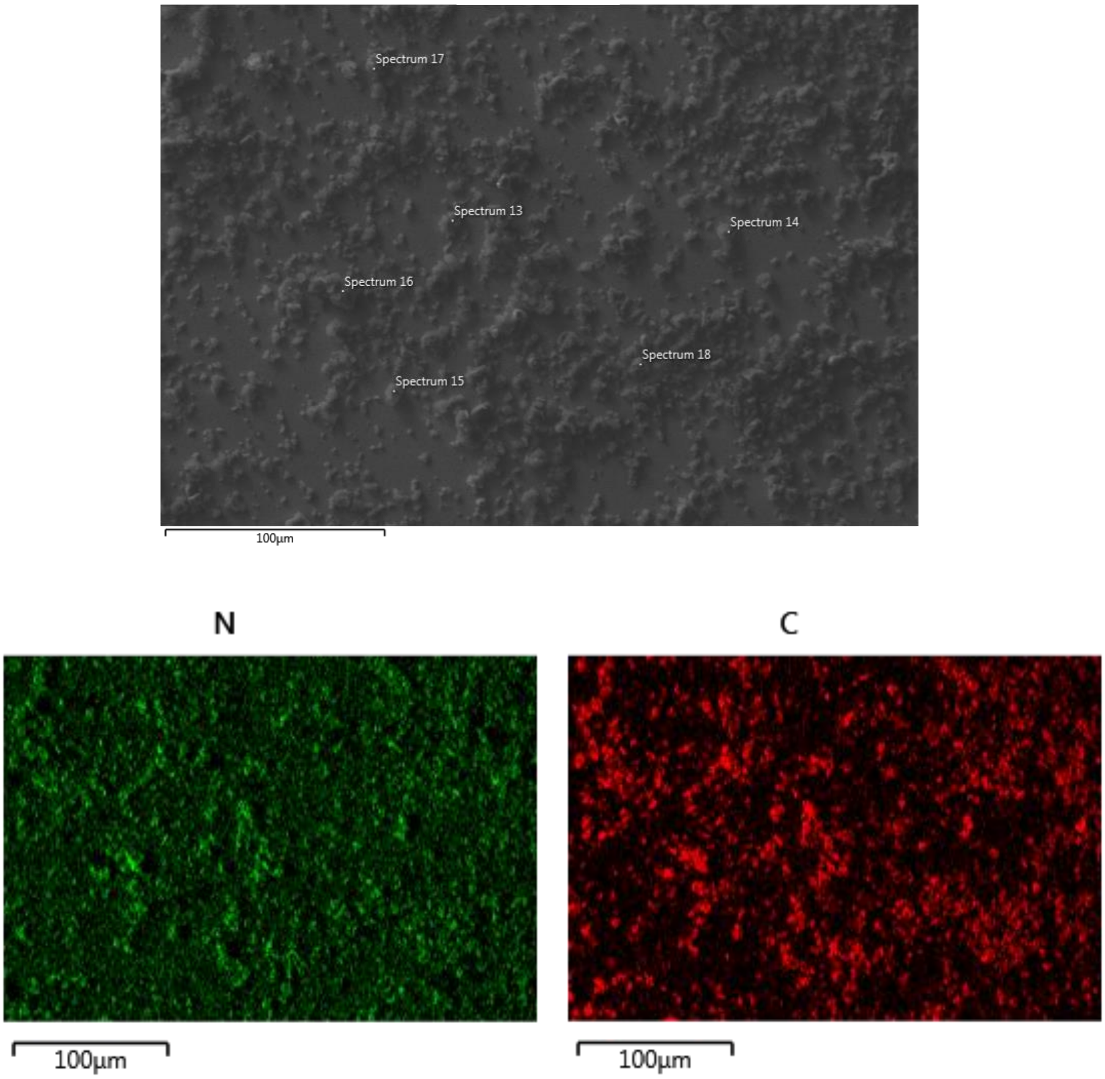

Figure S5: SEM image of mpg-CN and the corresponding elemental mapping for C and N.

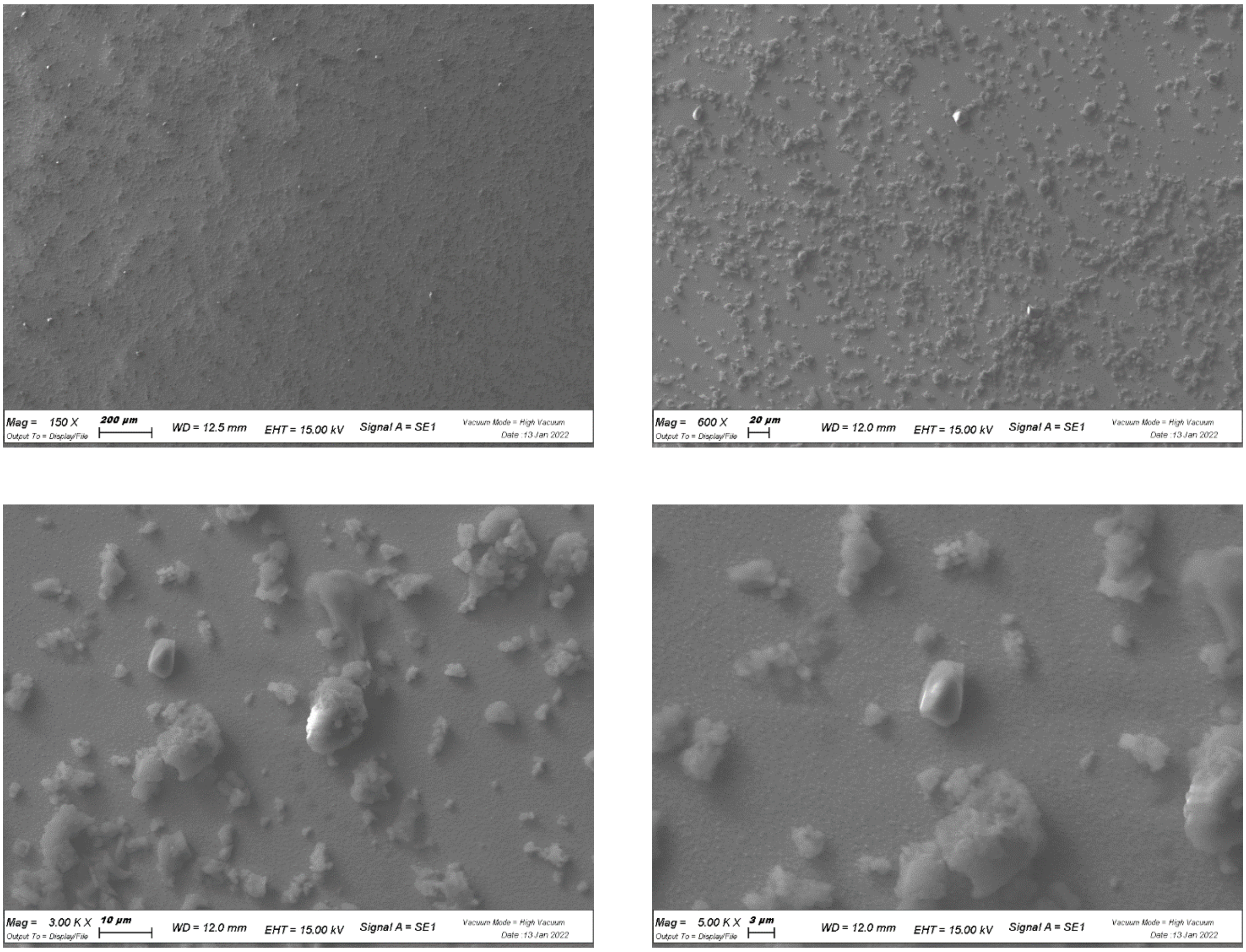

Figure S6: SEM images of mpg-CN with different magnifications.

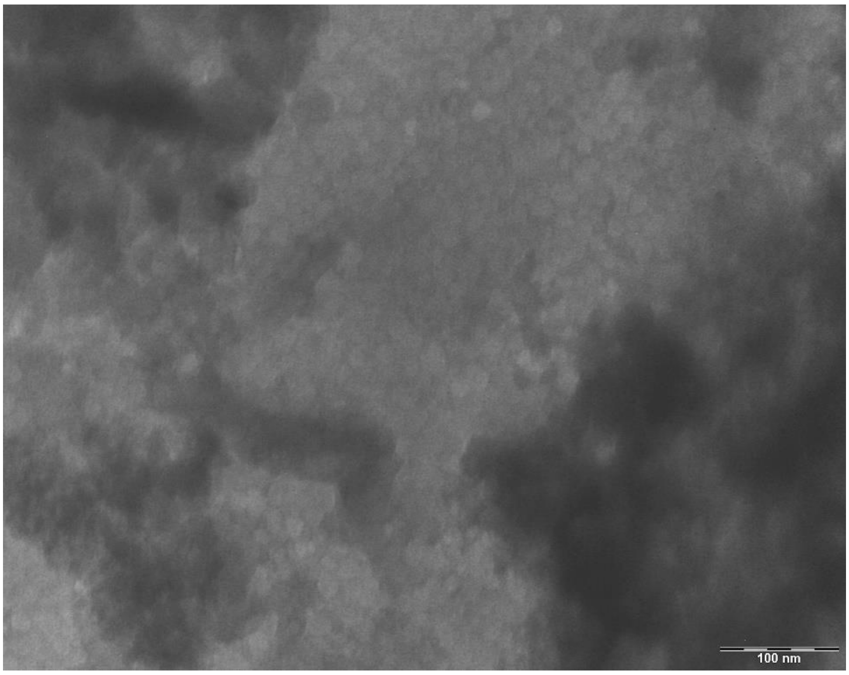

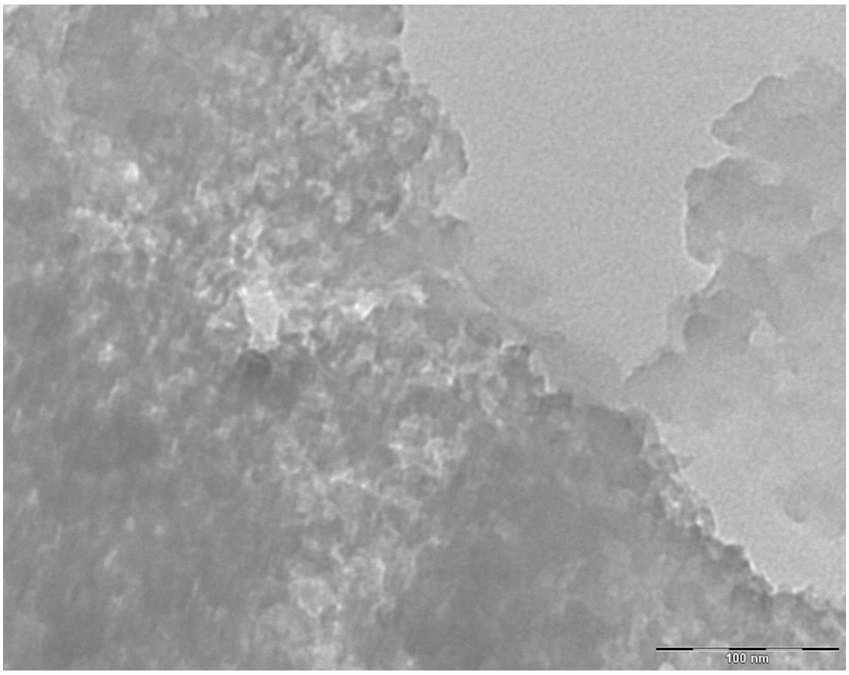

Figure S7: TEM images of mpg-CN.

TABLES

<u>Table S1.</u> Preliminary tests

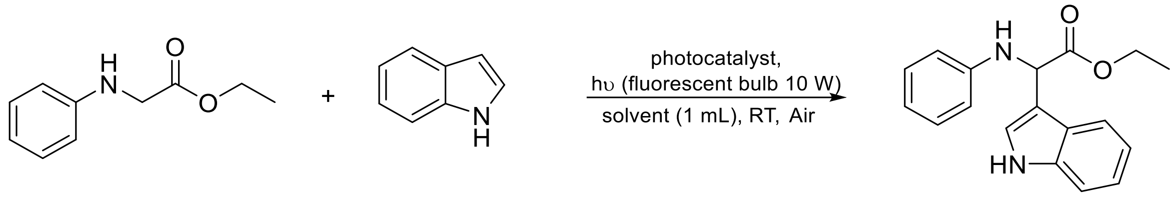

Entry	Catalyst	Solvent	Additive	Time (h)	Temperature (°C)	Yield (%)[e]
**1[a]**	g-CN	ACN	$Zn(OAc)_2$	72	25	≤5
**2[b]**	g-CN	ACN	$Zn(OAc)_2$	72	25	≤5
**3[b]**	g-CN	ACN	$Zn(OAc)_2$	72	50	≤5
**4[b]**	g-CN	THF	$Zn(OAc)_2$	72	25	-
**5[b]**	g-CN	DMF	$Zn(OAc)_2$	72	25	-
**6[c]**	g-CN	ACN	$Sc(CF_3SO_3)_3$	72	25	≤5
**8[d]**	mpg-CN	ACN	$Zn(OAc)_2$	72	25	12

[a]Reaction conditions: **1a** (0.1 mmol, 1 eq), **2a** (0.13 mmol, 1.3 eq), g-CN (10 mg), $Zn(OAc)_2$ (20 mol%,), ACN (1 mL), Air. [b]Reaction conditions: **1a** (0.1 mmol, 1 eq), **2a** (0.13 mmol, 1.3 eq), g-CN (10 mg), $Zn(OAc)_2$ (50 mol%,), solvent (1 mL), Air. [c]Reaction conditions: **1a** (0.1 mmol, 1 eq), **2a** (0.13 mmol, 1.3 eq), g-CN (10 mg), $Sc(CF_3SO_3)_3$ (20 mol%,), solvent (1 mL), Air. [d] Reaction conditions: **1a** (0.1 mmol, 1 eq), **2a** (0.13 mmol, 1.3 eq), mpg-CN (10 mg), $Zn(OAc)_2$ (20 mol%,), ACN (1 mL), Air. [e]NMR yield

Table S2. Variation of light power and tests without additive[a]

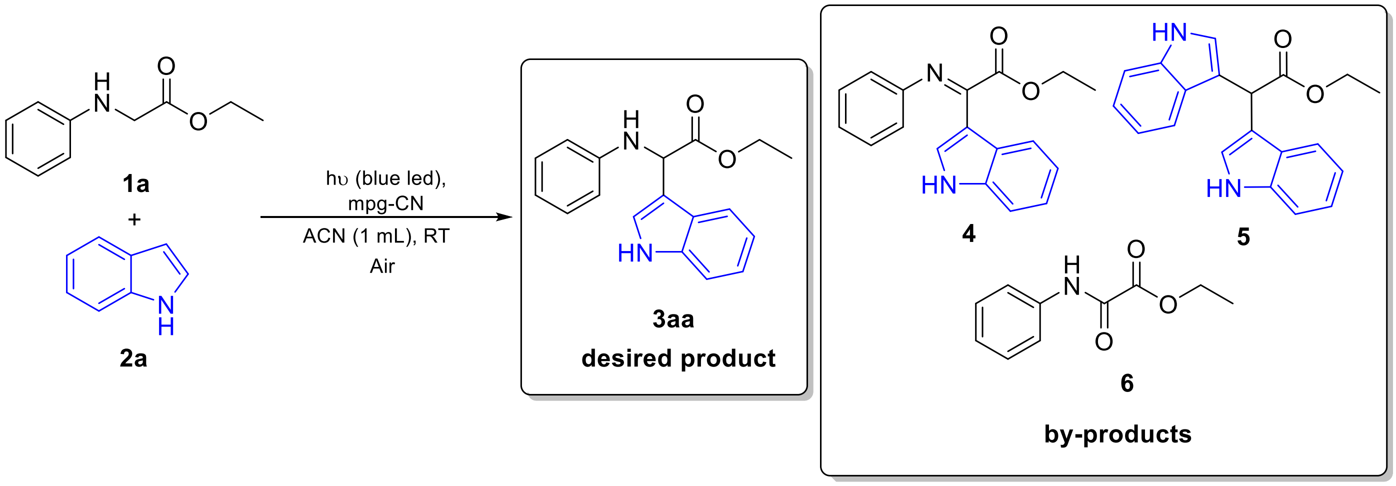

Entry	Additive	Time (h)	hv (W)	Conversion (%)	3aa(%)	4 (%)	5 (%)	6 (%)
**8**[b]	$Zn(OAc)_2$	72	10	28	23	-	5	-
**9**[c]	$Zn(OAc)_2$	72	40	47	21	11	11	≤ 5
**10**[d]	$Zn(OAc)_2$	72	40	64	24	30	6	≤ 5
**11**	-	72	10	42	28	5	9	-
**12**	-	72	20	68	37	21	10	-
**13**	-	72	40	100	54	32	7	7
**14**	-	16	40	85	58	15	7	5

[a]Reaction conditions: **1a** (0.1 mmol, 1 eq), **2a** (0.13 mmol, 1.3 eq), mpg-CN (10 mg), ACN (1 mL), Air. [b]Reaction conditions: **1a** (0.1 mmol, 1 eq), **2a** (0.13 mmol, 1.3 eq), mpg-CN (10 mg), $Zn(OAc)_2$ (20 mol%), ACN (1 mL), Air. [c]Reaction conditions: **1a** (0.1 mmol, 1 eq), **2a** (0.13 mmol, 1.3 eq), mpg-CN (10 mg), $Zn(OAc)_2$ (20 mol%), ACN (1 mL), Air. [d]Reaction conditions: **1a** (0.1 mmol, 1 eq), **2a** (0.13 mmol, 1.3 eq), mpg-CN (10 mg), $Zn(OAc)_2$ (10 mol%), ACN (1 mL), Air.

Table S3. Effects of light, temperature[a] and catalyst

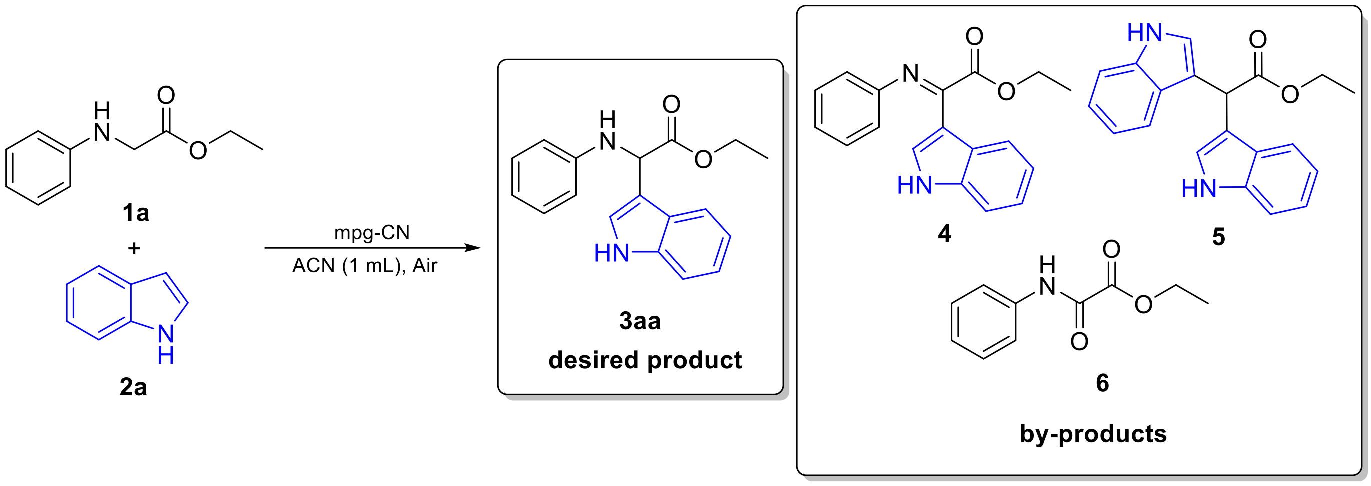

Entry	Catalyst	Time (h)	Temperature (°C)	Conditions	Conversion (%)	3aa (%)	4 (%)	5(%)	6 (%)
**15**[b]	mpg-CN	16	25	dark	-	-	-	-	-
**16**[c]	-	16	25	dark	-	-	-	-	-
**17**[d]	-	16	25	light	8	≤5	-	-	≤5
**18**[e]	mpg-CN	16	30	dark	-	-	-	-	-
**19**[f]	-	16	30	dark	-	-	-	-	-
**20**[g]	mpg-CN	16	25	light/argon	≤5	≤5	-	-	-

[a]vial temperature achieved after overnight irradiation (30 °C). [b]Reaction conditions: **1a** (0.1 mmol, 1 eq), **2a** (0.13 mmol, 1.3 eq), mpg-CN (10 mg), ACN (1 mL), Air. [c]Reaction conditions: **1a** (0.1 mmol, 1 eq), **2a** (0.13 mmol, 1.3 eq), MeCN (1 mL), Air. [d]Reaction conditions: **1a** (0.1 mmol, 1 eq), **2a** (0.13 mmol, 1.3 eq), ACN (1 mL), Air, hν (blue led 40 W). [e]Reaction conditions: **1a** (0.1 mmol, 1 eq), **2a** (0.13 mmol, 1.3 eq), mpg-CN (10 mg), T (30 °C, oil bath), ACN (1 mL), Air. [f]Reaction conditions: **1a** (0.1 mmol, 1 eq), **2a** (0.13 mmol, 1.3 eq), T (30 °C, oil bath), ACN (1 mL), Air. [g]Reaction conditions: **1a** (0.1 mmol, 1 eq), **2a** (0.13 mmol, 1.3 eq), mpg-CN (10 mg), ACN (1 mL), argon atmosphere, hν (blue led 40 W).

Table S4. Solvents screening[a]

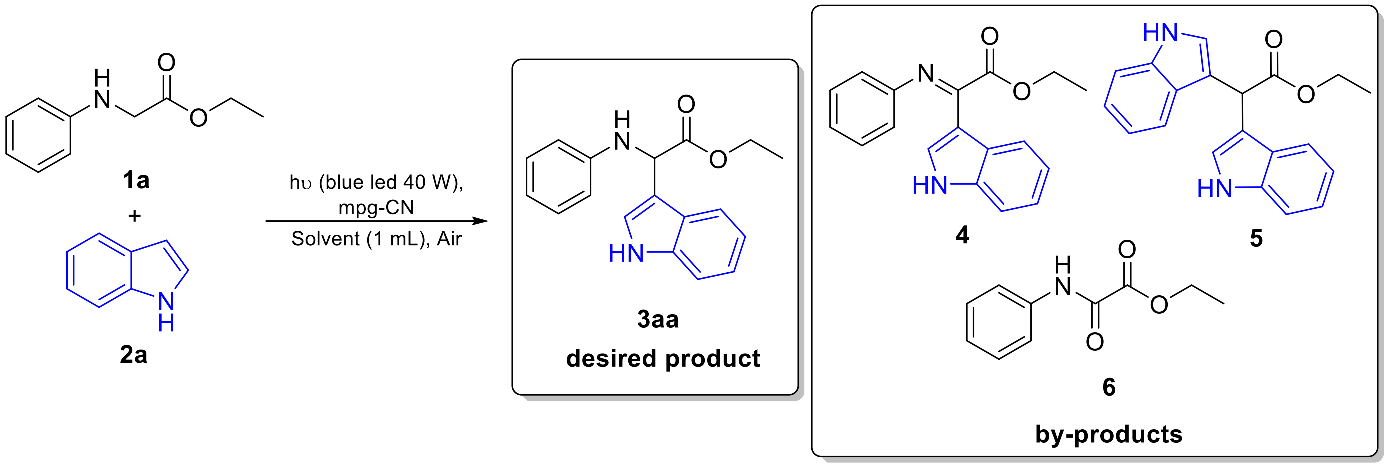

Entry	Solvent	Time (h)	Conversion (%)	3aa (%)	4 (%)	5(%)	6 (%)
**21**	MeCN	16	85	58	15	7	5
**22**	Acetone	16	74	55	19	-	-
**23**	DMF	16	72	39	30	-	≤ 5
**24**	EtOH	16	0	-	-	-	-
**25**	H_2O	72	42	30	-	-	12
**26**	EtOH/H_2O (2:1)	48	46	12	32	≤ 5	-
**27**	THF	16	85	37	-	16	32
**28**	EtOAc	16	100	64	15	11	10
**29**	DCM	16	70	35	19	16	-
**30**	DMSO	16	41	13	-	28	-
**31**	Toluene	16	87	50	15	22	-
**32**	Methyl-THF	16	81	50	9	13	9
**33**	(+)-Limonene	48	72	51	8	8	5
**34**	isosorbide dimethyl ether	48	0	-	-	-	-
**35**	γ-Valerolactone	16	55	45	-	-	10

[a]Reaction conditions: **1a** (0.1 mmol, 1 eq), **2a** (0.13 mmol, 1.3 eq), mpg-CN (10 mg), hν (blue led 40 W), solvent (1 mL), Air.

Table S5. Effects of light power[a]

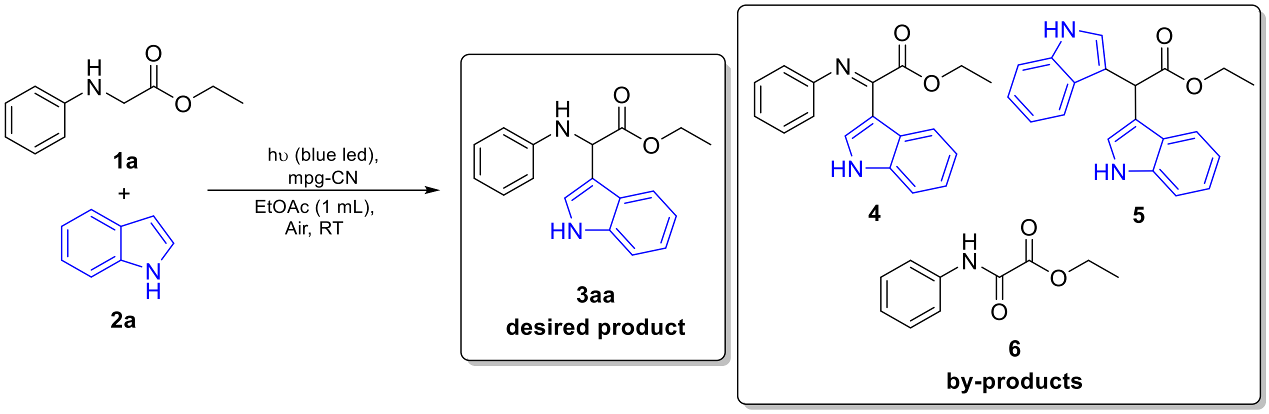

Entry	Time (h)	Power (W)	Conversion (%)	3aa (%)	4 (%)	5 (%)	6 (%)
**36**	16	10	65	54	≤5	≤5	≤5
**37**	16	20	100	71	12	7	10
**38**	16	40	100	71	12	7	10

[a]Reaction conditions: **1a** (0.1 mmol, 1 eq), **2a** (0.13 mmol, 1.3 eq), mpg-CN (10 mg), hv (power indicated in table), EtOAc (1 mL), Air.

Table S6. Effects of catalytic loading[a]

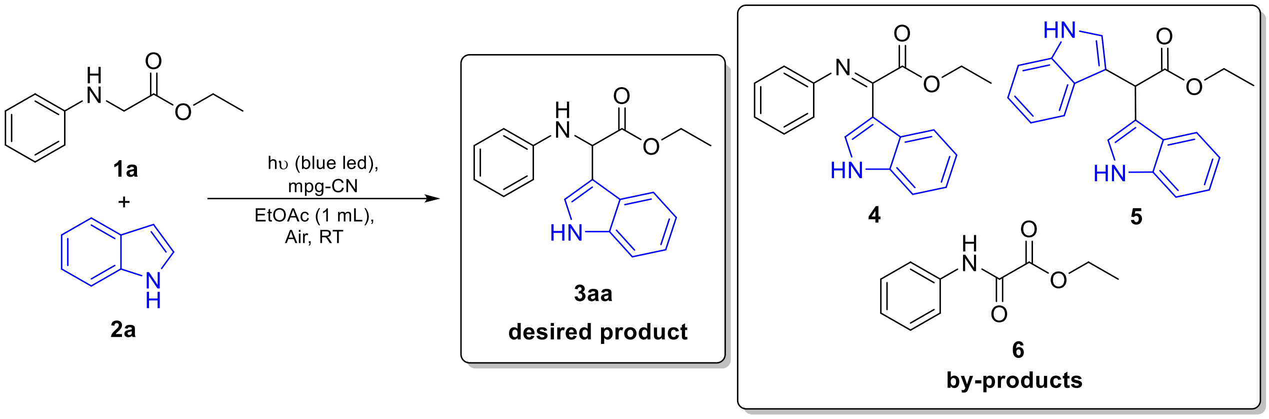

Entry	Time (h)	Catalytic loading (mg)	Conversion (%)	3aa (%)	4 (%)	5 (%)	6 (%)
**39**	16	2.5	9	≤5	-	≤5	6
**40**	16	5	13	≤5	≤5	-	8
**41**	16	10	82	68	≤5	≤5	7
**42**	16	15	20	7	≤5	-	11

[a]Reaction conditions: **1a** (0.1 mmol, 1 eq), **2a** (0.13 mmol, 1.3 eq), mpg-CN (quantity indicated in table), hν (blue led 20 W), EtOAc (1 mL), Air.

BY-PRODUCTS IDENTIFICATION

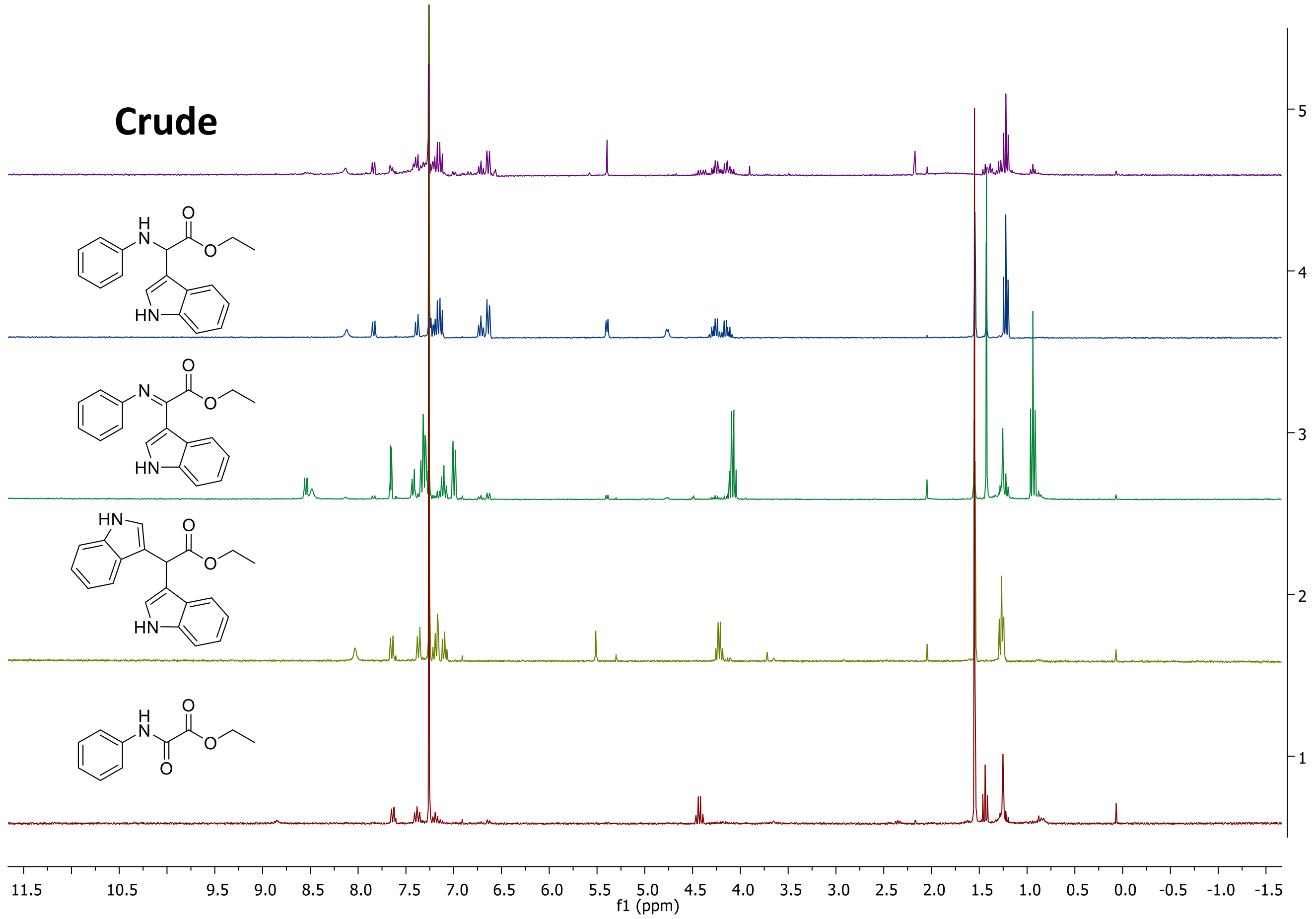

Figure S8: Superimposed spectra of a crude reactions, product and all by-products.

PEAK LIST OF BY-PRODUCTS

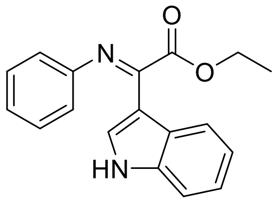

***Ethyl (E)-2-(1H-indol-3-yl)-2-(phenylimino)acetate* (4):** ^{1}H NMR (300 MHz, $CDCl_3$) δ 8.55 (dd, *J* = 6.4, 3.0 Hz, 1H), 8.48 (s, 1H), 7.66 (d, *J* = 3.0 Hz, 1H), 7.46 – 7.39 (m, 1H), 7.35 – 7.27 (m, 4H), 7.15 – 7.07 (m, 1H), 6.99 (d, *J* = 7.3 Hz, 2H), 4.08 (q, *J* = 7.1 Hz, 2H), 0.94 (t, *J* = 7.1 Hz, 3H). $^{13}C\{^1H\}$ NMR (101 MHz, $CDCl_3$) δ 165.1, 155.8, 151.4, 136.8, 130.0, 129.3, 128.7, 125.3, 124.1, 124.0, 123.1, 122.2, 120.1, 113.7, 111.3, 61.2, 13.7. HRMS (ESI) *m/z* $[M + H]^+$ calcd for $C_{18}H_{17}N_2O_2$ 293.1285, found 293.1278.

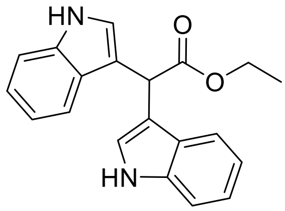

***Ethyl 2,2-di(1H-indol-3-yl)acetate* (5):** ^{1}H NMR (300 MHz, $CDCl_3$) δ 8.03 (s, 2H), 7.65 (d, *J* = 7.9 Hz, 2H), 7.37 (d, *J* = 7.9 Hz, 2H), 7.23 – 7.13 (m, 4H), 7.13 – 7.05 (m, 2H), 5.51 (s, 1H), 4.22 (q, *J* = 7.1 Hz, 2H), 1.27 (s, *J* = 7.1 Hz, 3H). $^{13}C\{^1H\}$ NMR (101 MHz, $CDCl_3$) δ 173.2, 136.3, 126.7, 123.2, 122.1, 119.5(d, *J* = 19.9 Hz), 113.87 (s), 111.12 (s), 61.02 (s), 40.66 (s), 14.24 (s), 1.00 (s). HRMS (ESI) *m/z* $[M + H]^+$ calcd for $C_{20}H_{19}N_2O_2$ 319.1441, found 319.1452.

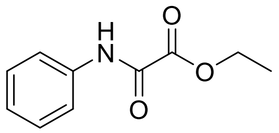

***Ethyl 2-oxo-2-(phenylamino)acetate* (6):** ^{1}H NMR (300 MHz, $CDCl_3$) δ 8.85 (s, 4H), 7.64 (d, *J* = 8.5 Hz, 10H), 7.38 (t, *J* = 7.9 Hz, 10H), 7.19 (t, *J* = 7.4 Hz, 6H), 4.43 (q, *J* = 7.1 Hz, 9H), 1.44 (t, *J* = 7.1 Hz, 15H). $^{13}C\{^1H\}$ NMR (101 MHz, $CDCl_3$) δ 167.5, 129.3, 125.6, 119.8, 63.8, 31.0, 29.8, 14.1. HRMS (ESI) *m/z* $[M + H]^+$ calcd for $C_{10}H_{12}NO_3$ 194.0812, found 194.0818.

UNSUCCESSFUL SCOPE COMPOUNDS

Electrophiles

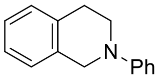

2-phenyl-1,2,3,4-tetrahydroisoquinoline

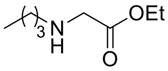

ethyl 2-(butylamino)acetate

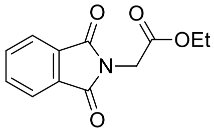

ethyl 2-(1,3-dioxoisoindolin-2-yl)acetate

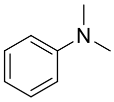

N,*N*-dimethylaniline

Nucleophiles

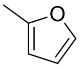

2-methylfuran

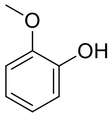

2-methoxyphenol

Reaction conditions: **Electrophile/1a** (0.13 mmol), **Nucleophile/2a** (0.1 mmol), 1 mL of EtOAc and 10 mg of mpg-CN were placed in a 5 mL vial equipped with a magnetic bar and a balloon filled with air; the reaction was stirred under 20 W blue led light for 16 hours.

NMR OF BY-PRODUCTS

^{1}H-NMR (300 MHz, $CDCl_3$), ^{13}C{^{1}H}-NMR (101 MHz, $CDCl_3$) of *Ethyl (E)-2-(1H-indol-3-yl)-2-(phenylimino)acetate* (4)

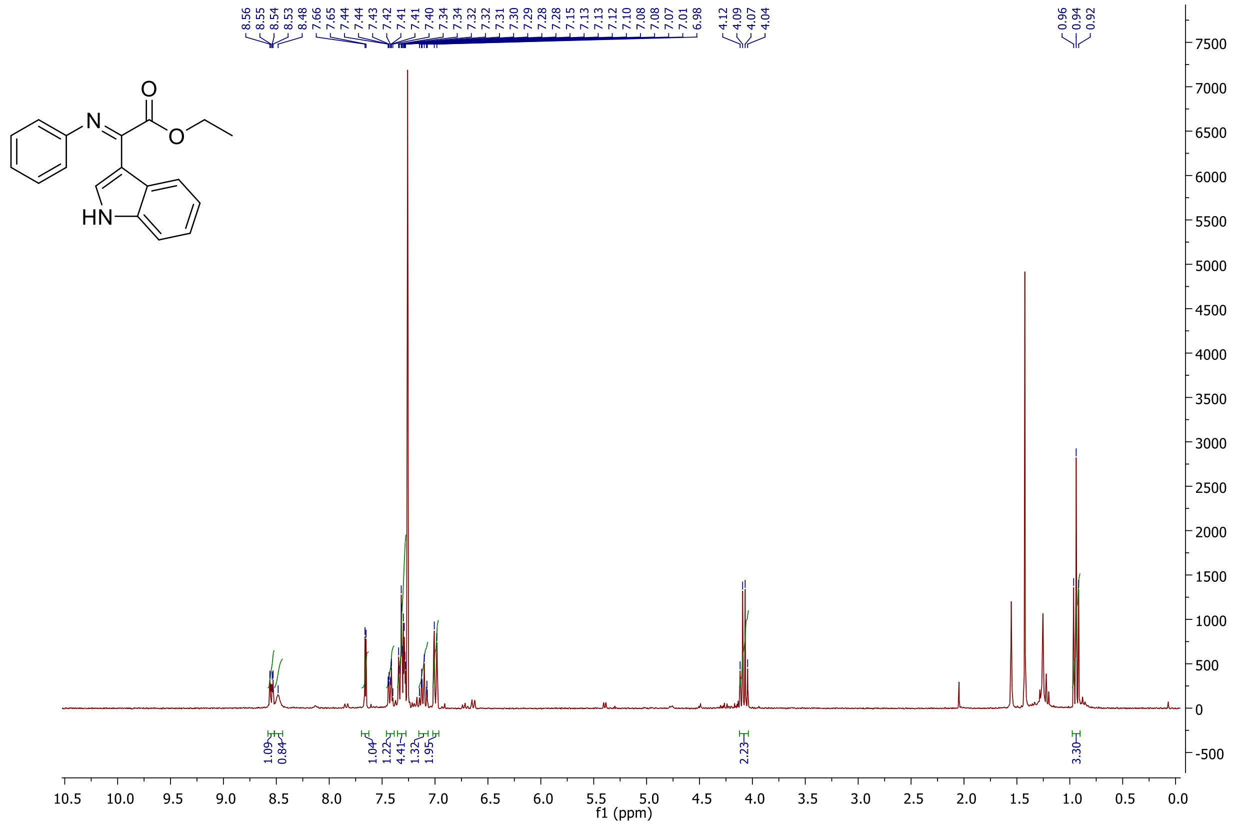

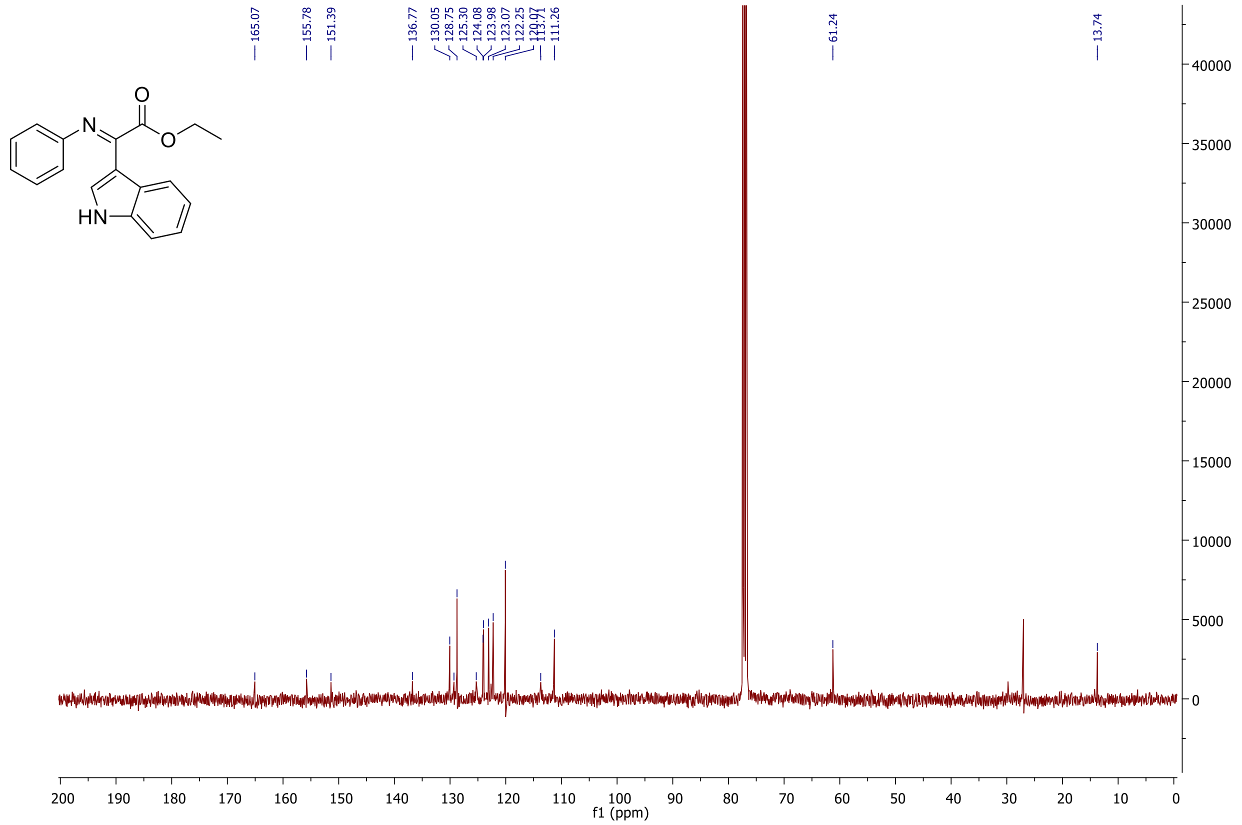

^{1}H-NMR (300 MHz, $CDCl_3$), ^{13}C{^{1}H}-NMR (101 MHz, $CDCl_3$) of *Ethyl 2,2-di(1H-indol-3-yl)acetate* (5)

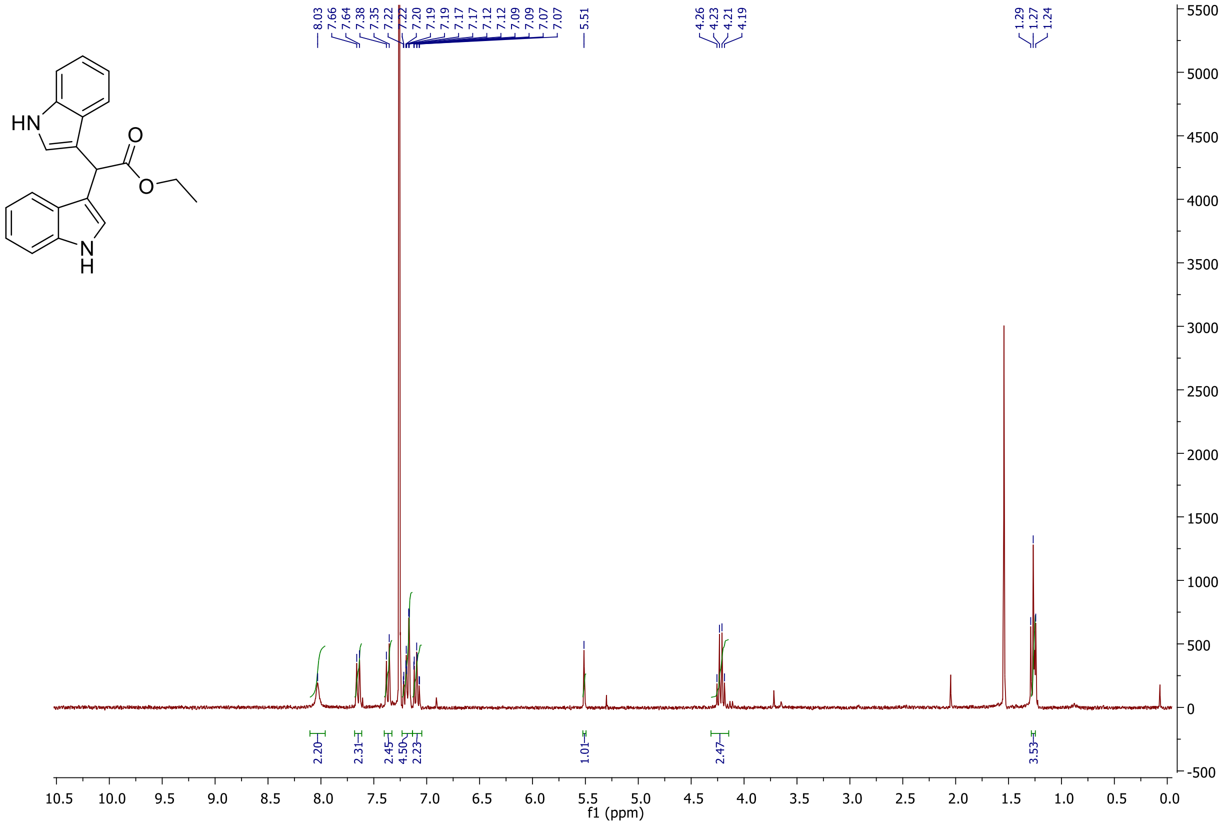

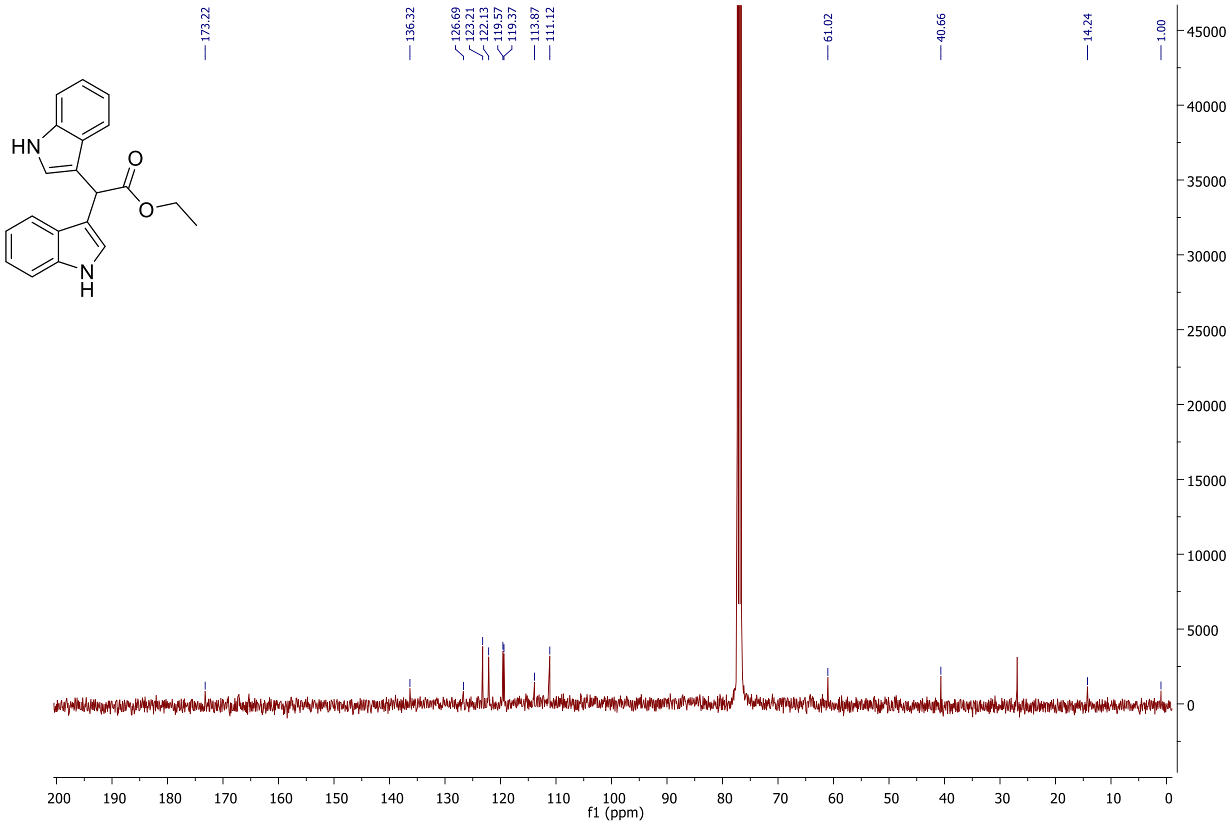

^{1}H-NMR (300 MHz, $CDCl_3$), $^{13}C\{^{1}H\}$-NMR (101 MHz, $CDCl_3$) of *Ethyl 2-oxo-2-(phenylamino)acetate* (6)

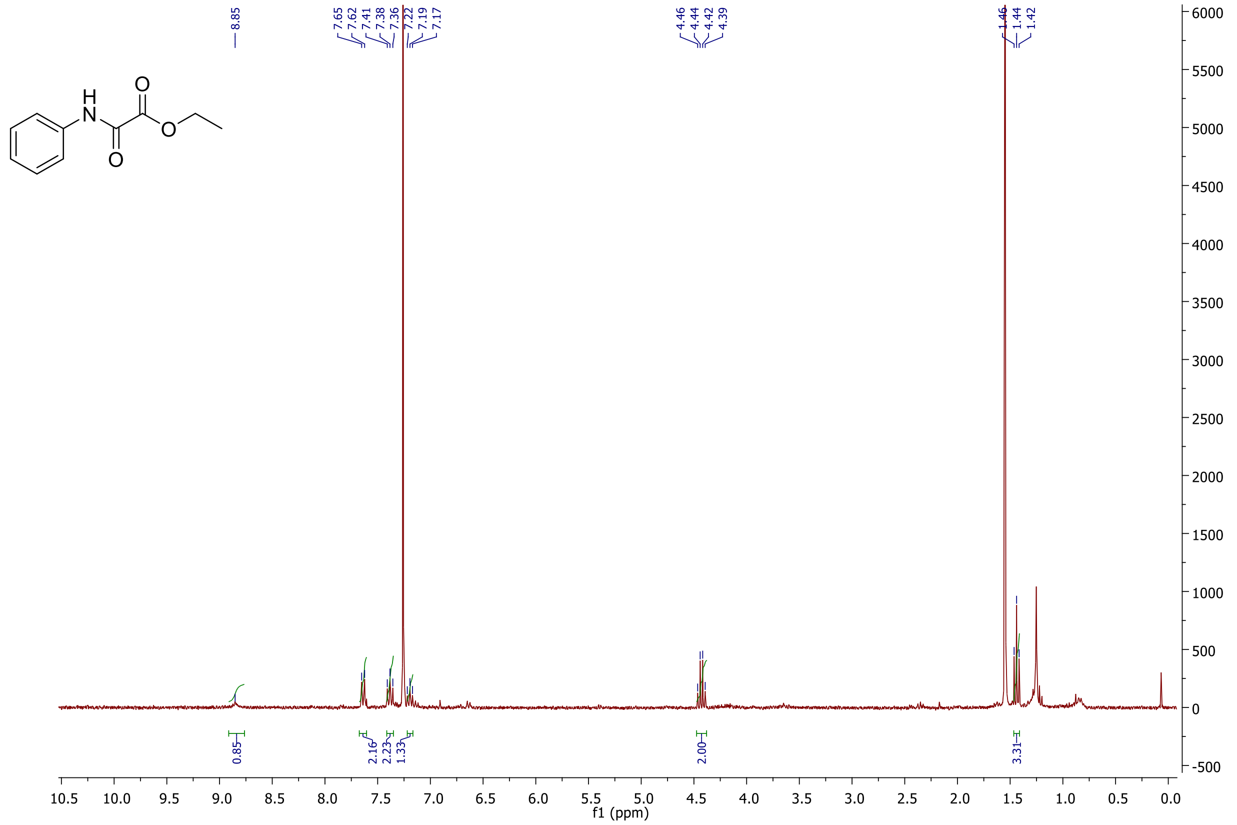

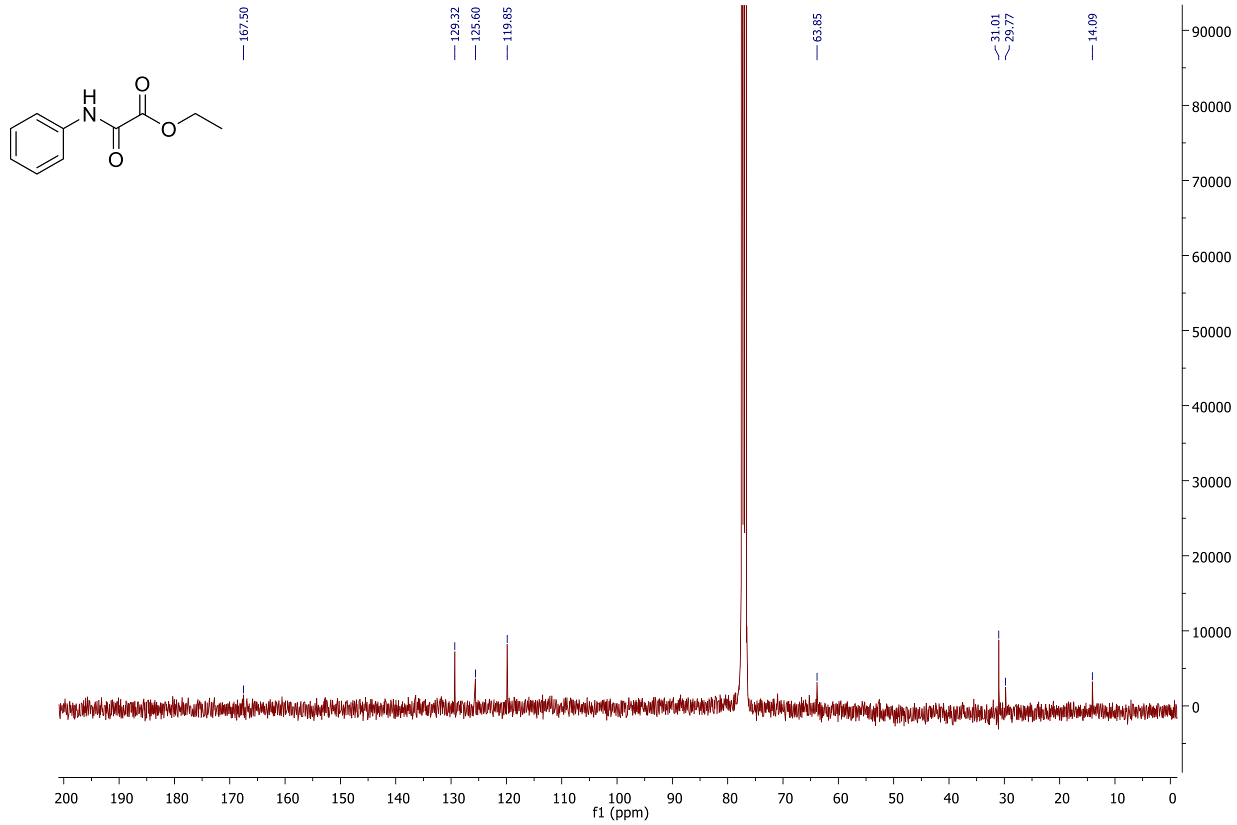

NMR OF STARTING COMPOUNDS

^{1}H-NMR (300 MHz, $CDCl_3$), ^{13}C{^{1}H}-NMR (101 MHz, $CDCl_3$) of *N-Phenylglycine ethyl ester (1a)*

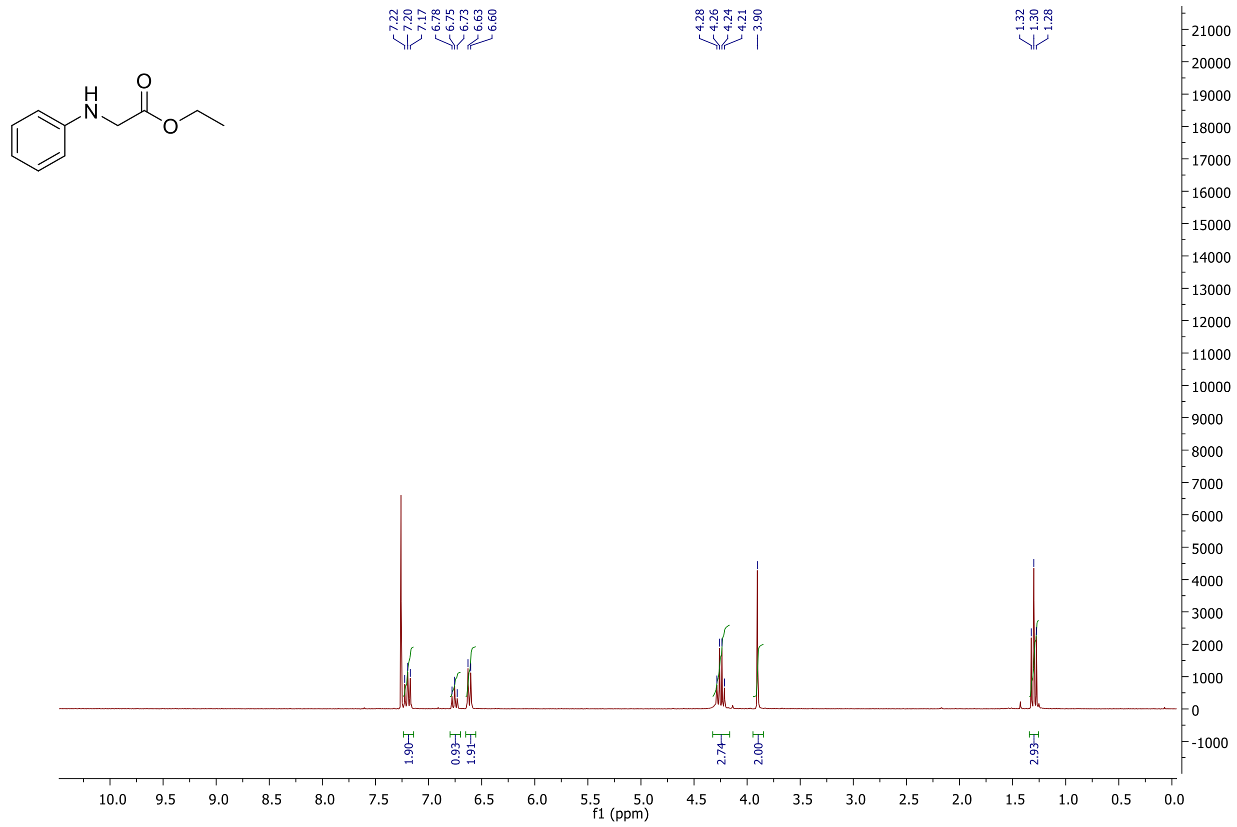

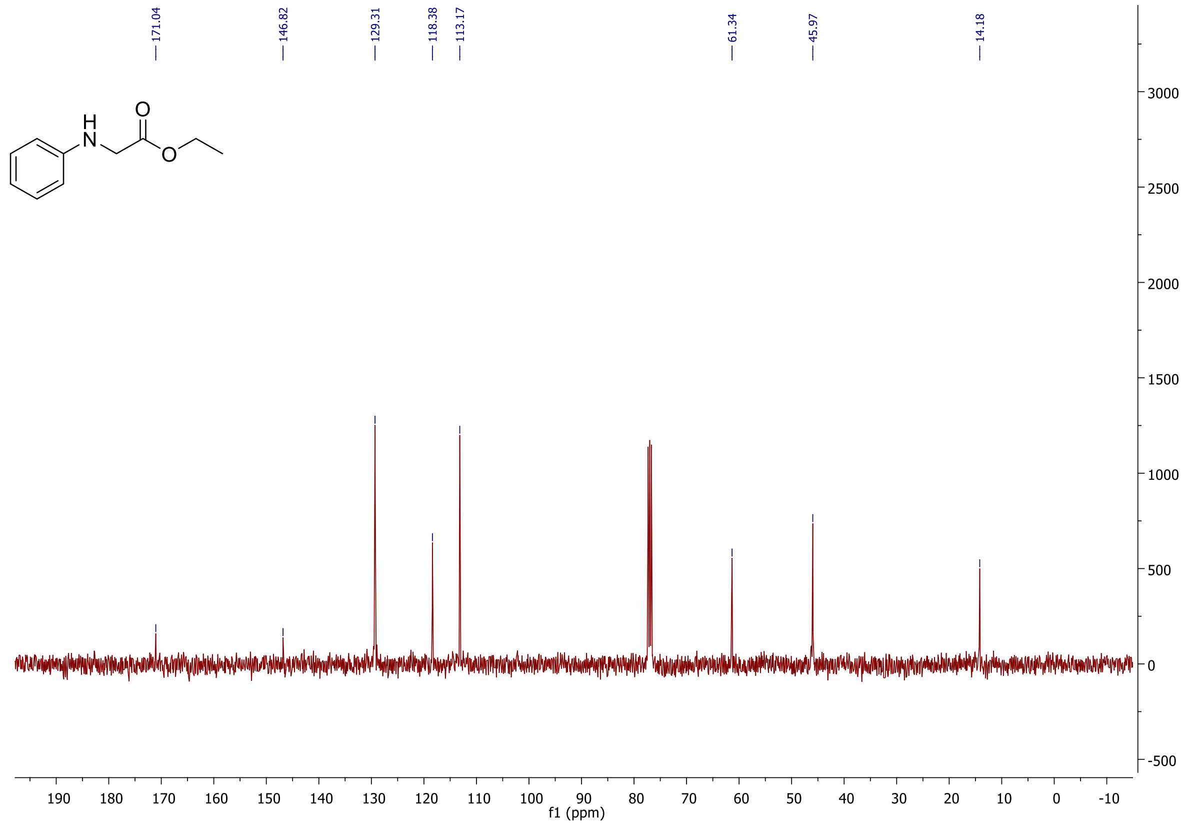

^{1}H-NMR (300 MHz, $CDCl_3$), ^{13}C{^{1}H}-NMR (101 MHz, $CDCl_3$) of *N-(4-Methylphenyl)glycine ethyl ester* (1b)

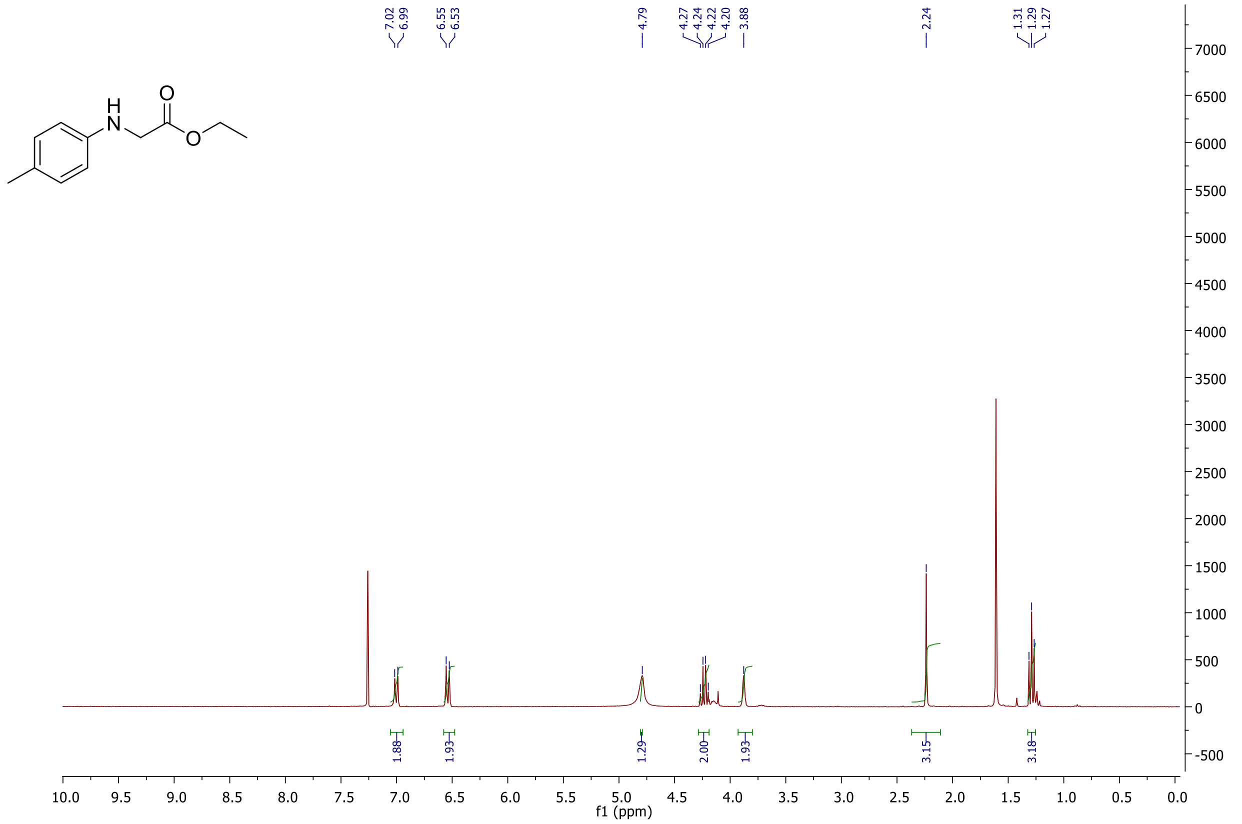

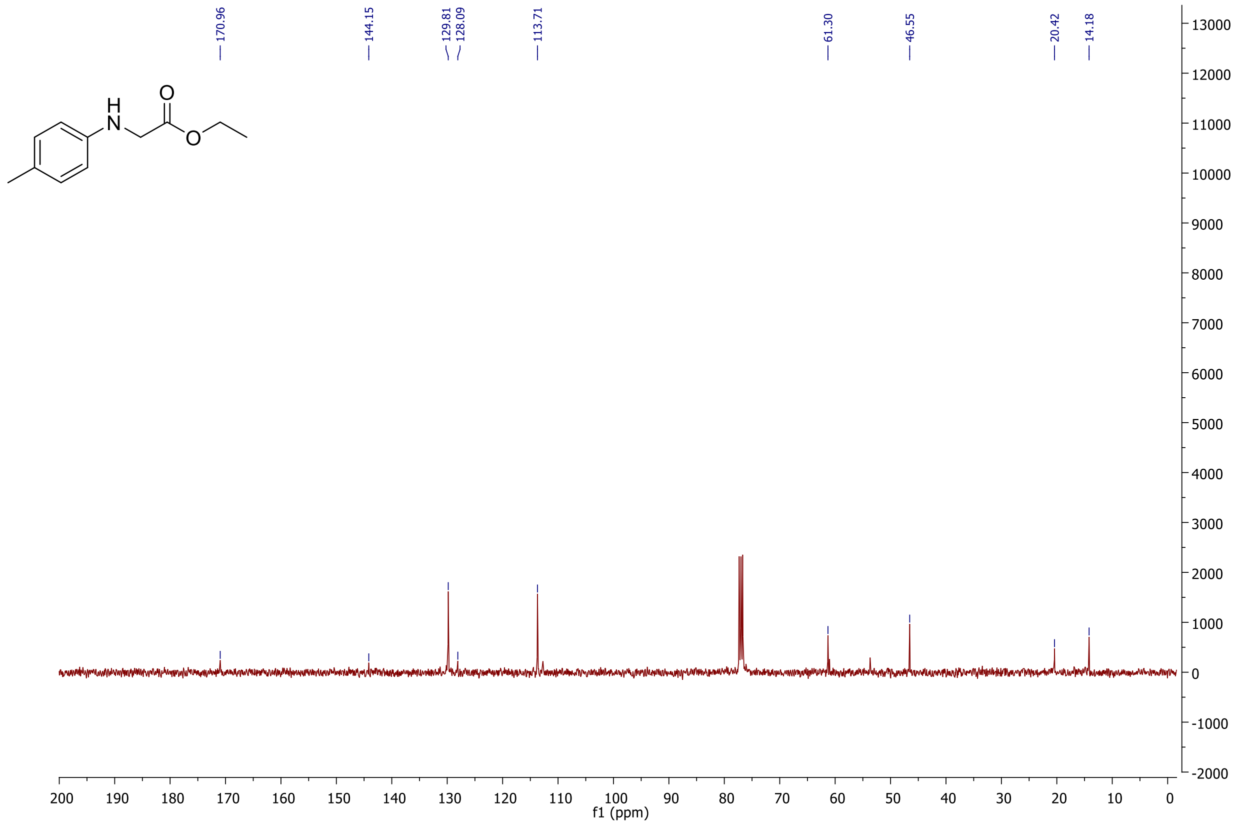

^{1}H-NMR (300 MHz, $CDCl_3$), ^{13}C{^{1}H}-NMR (101 MHz, $CDCl_3$) of *N-(4-Methoxyphenyl)glycine ethyl ester* (1c)

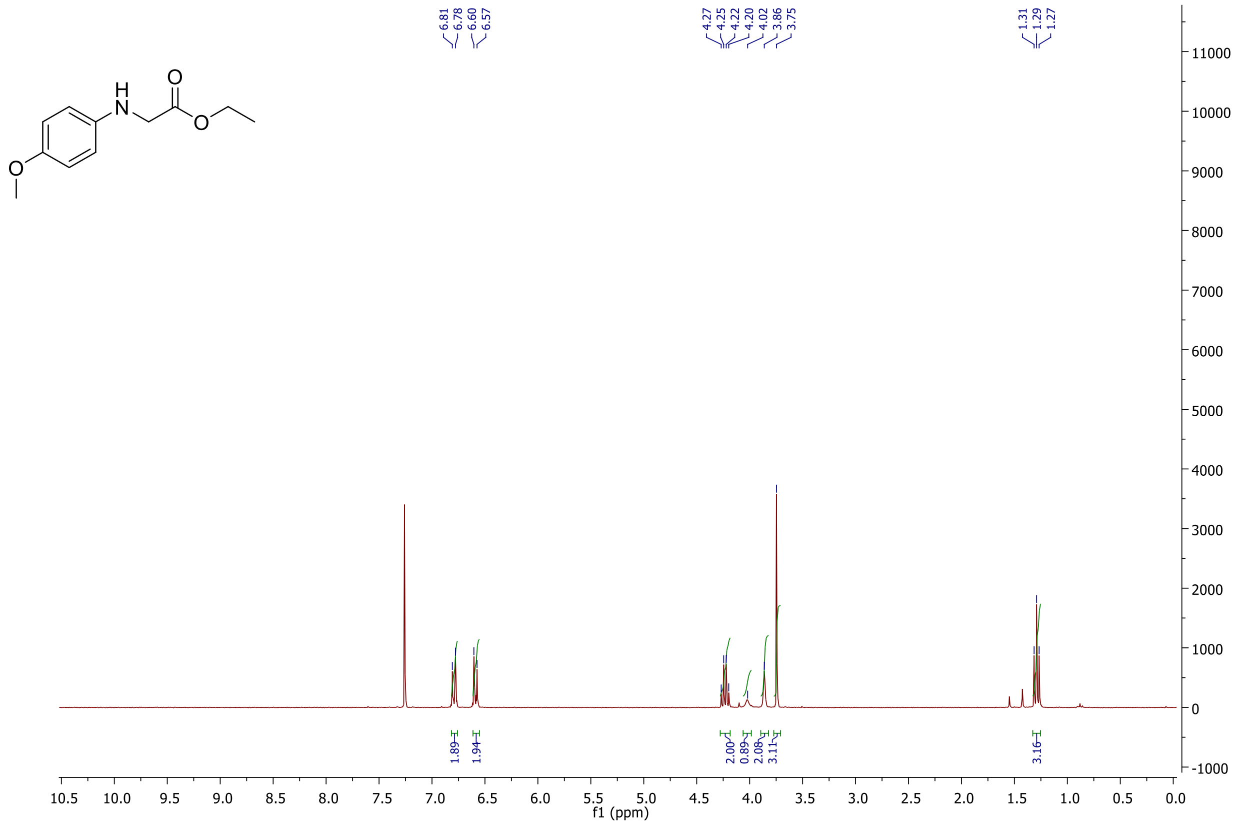

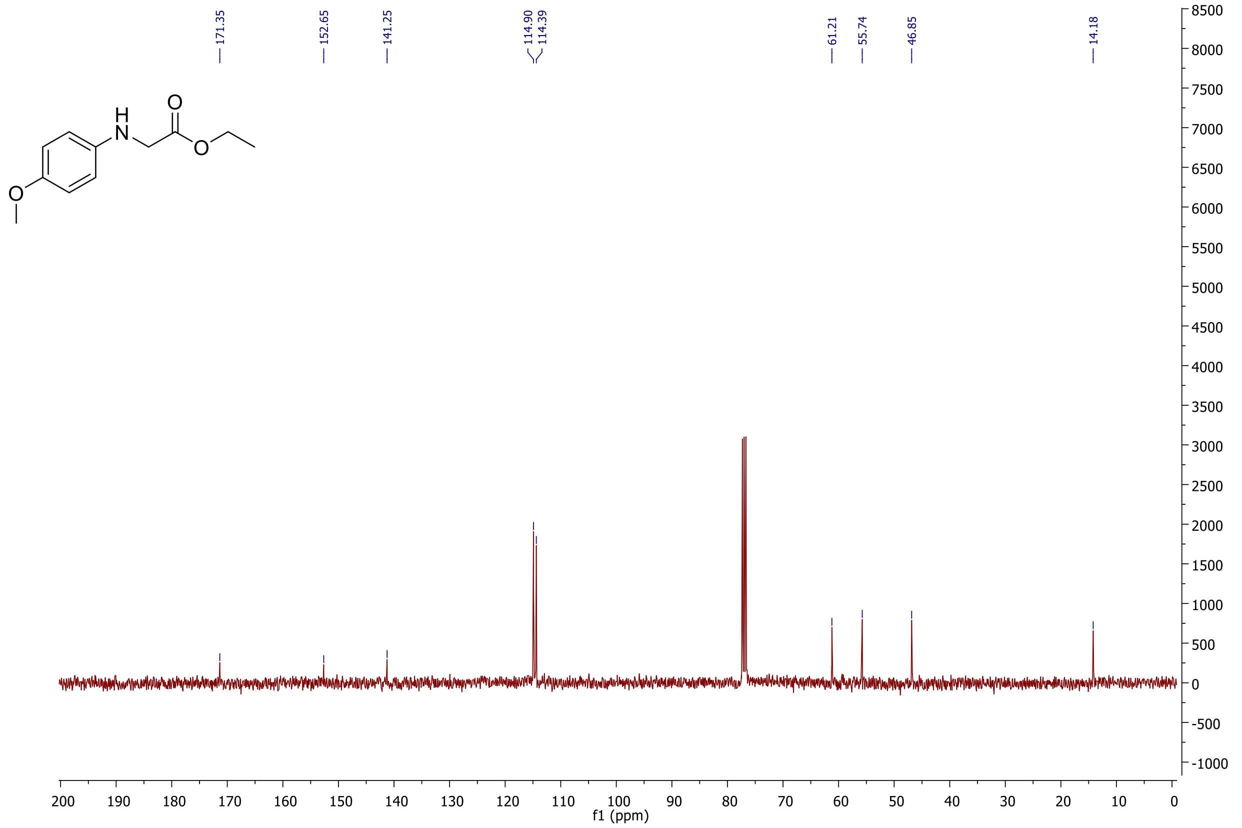

^{1}H-NMR (300 MHz, $CDCl_3$), ^{13}C{^{1}H}-NMR (101 MHz, $CDCl_3$) of *N-(4-Fluorophenyl)glycine ethyl ester* (1d)

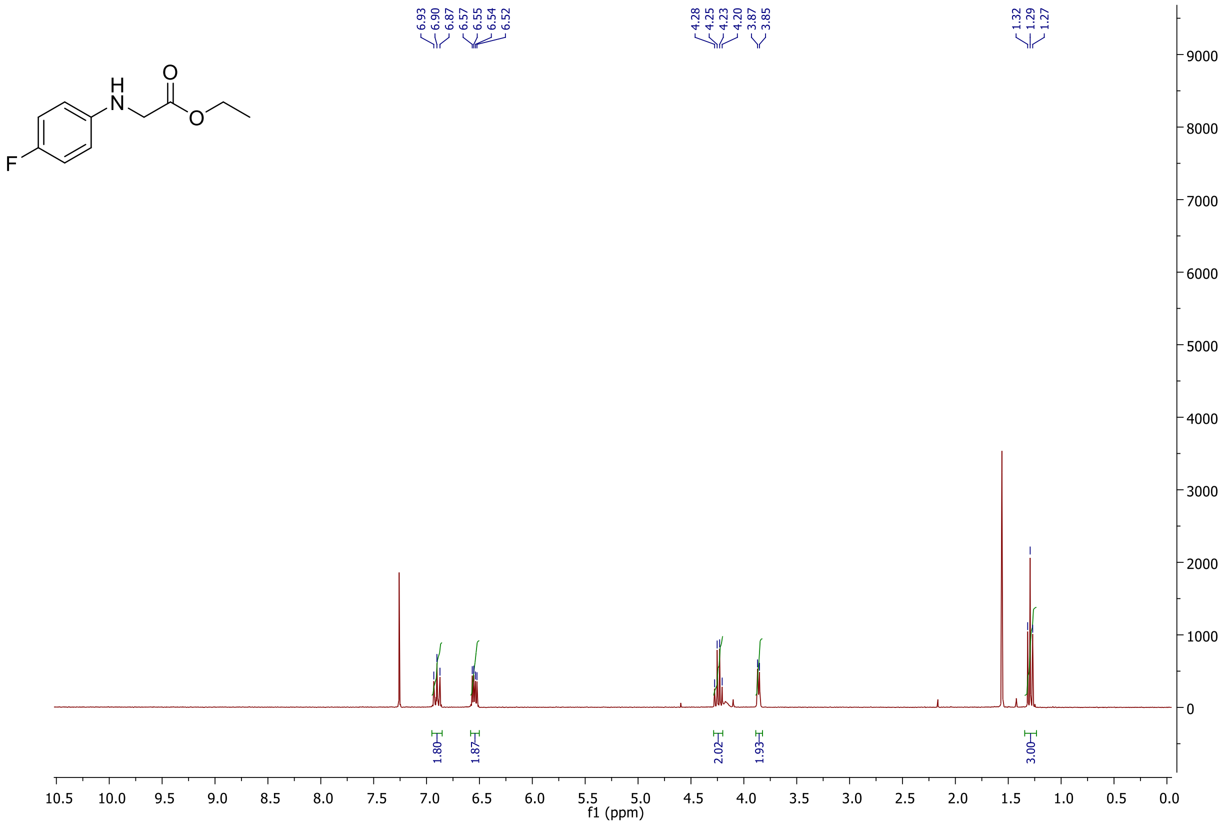

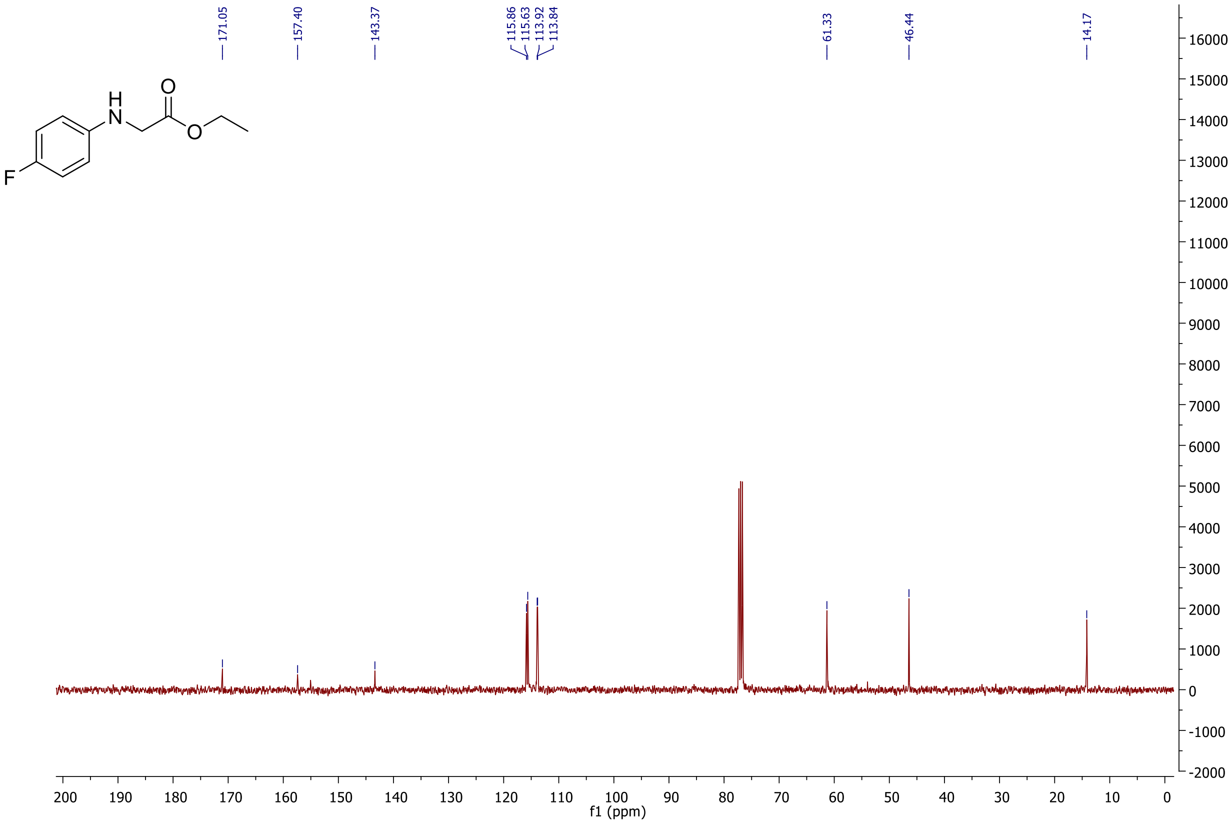

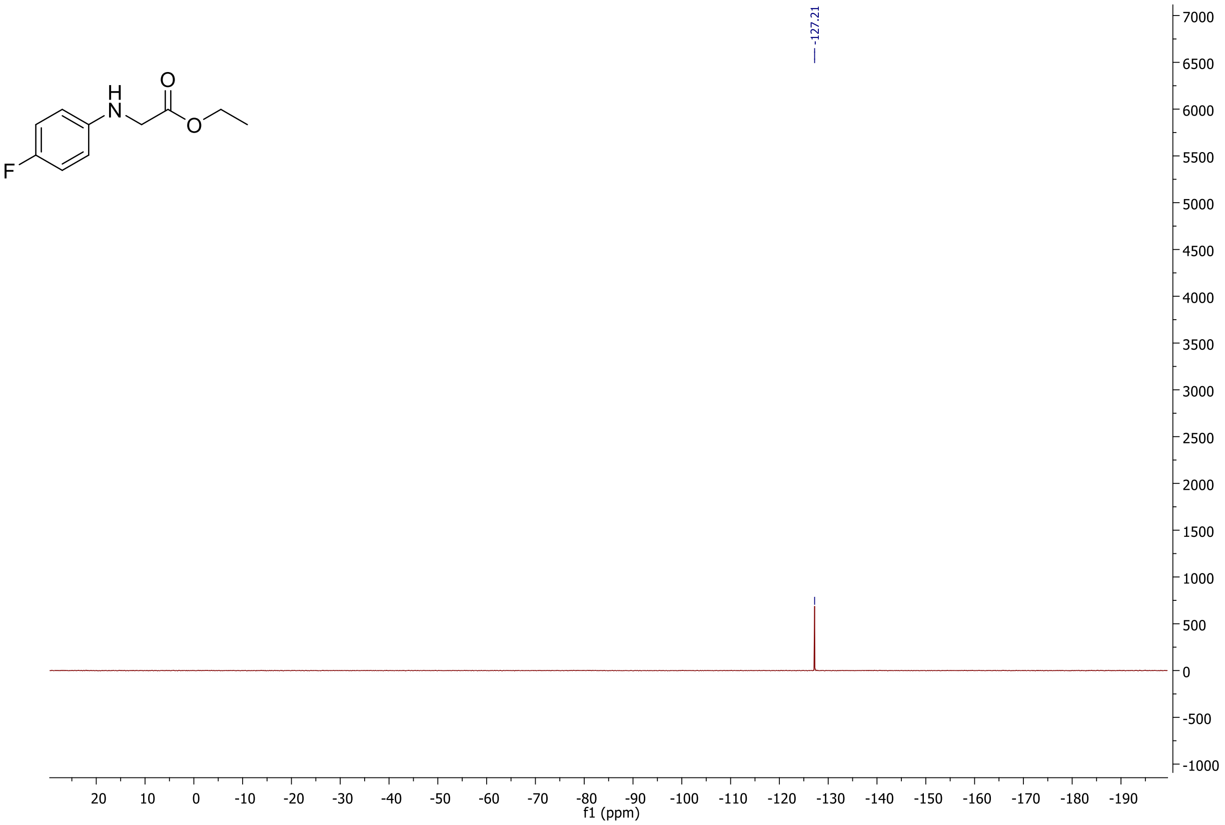
F
H
N
O
O
-127.21
f1 (ppm)

^{1}H-NMR (300 MHz, $CDCl_3$), ^{13}C{^{1}H}-NMR (101 MHz, $CDCl_3$) of *N-(4-Chlorophenyl)glycine ethyl ester* (1e)

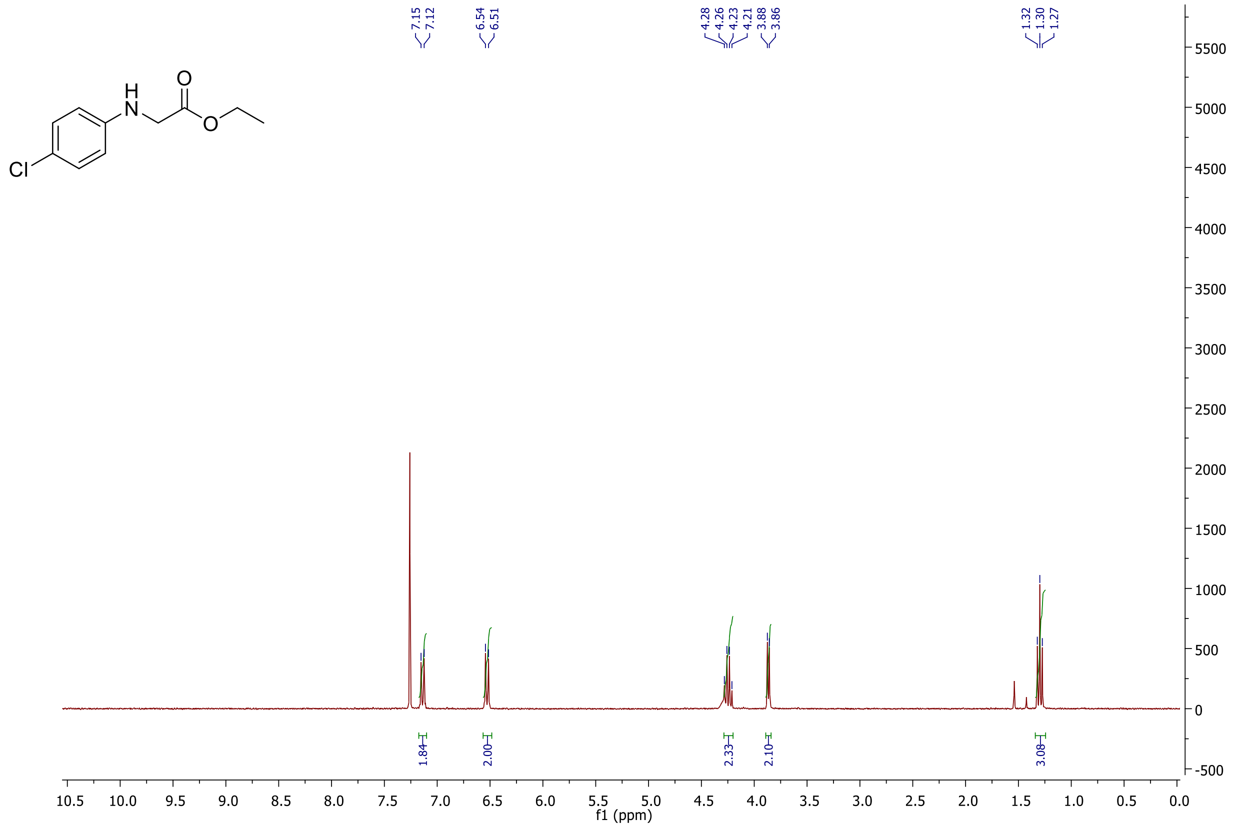

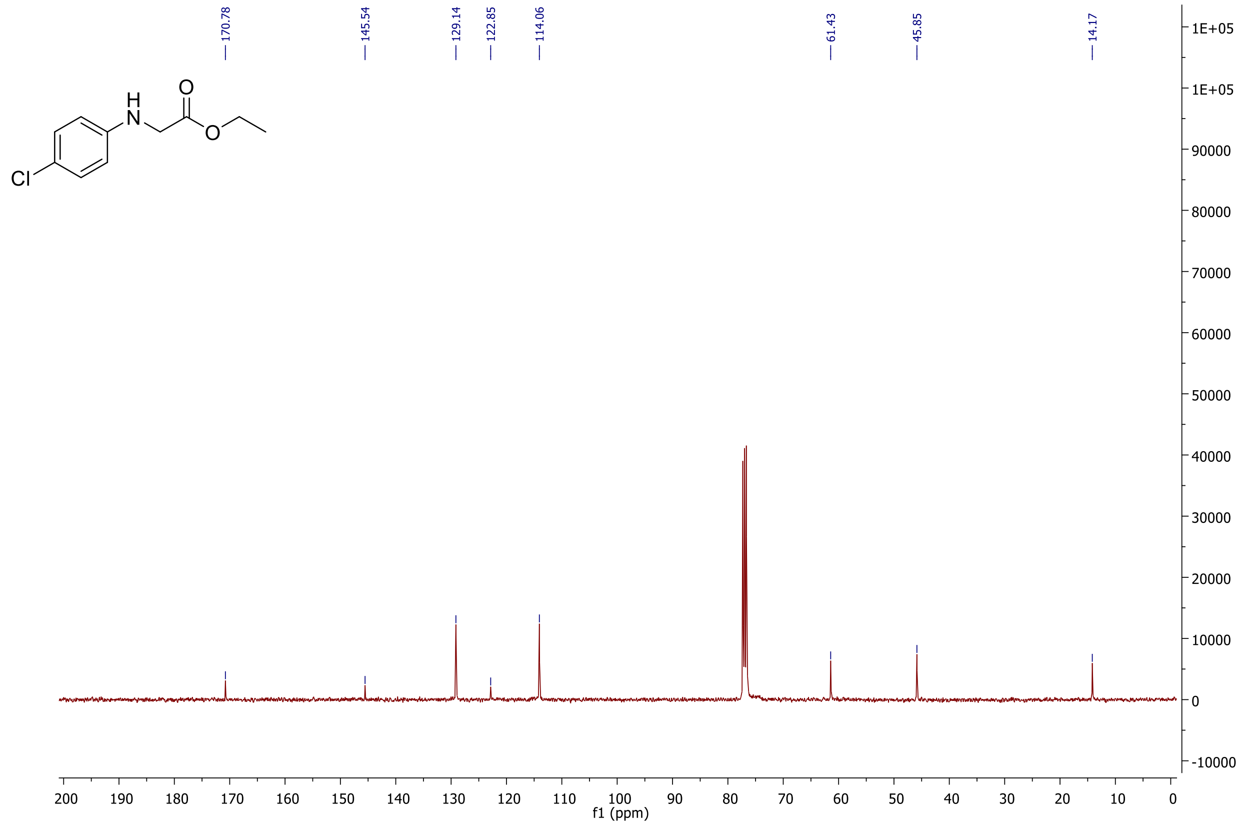

^{1}H-NMR (300 MHz, $CDCl_3$), $^{13}C\{^1H\}$-NMR (101 MHz, $CDCl_3$) of *N-(4-Bromophenyl)glycine ethyl ester* (1f)

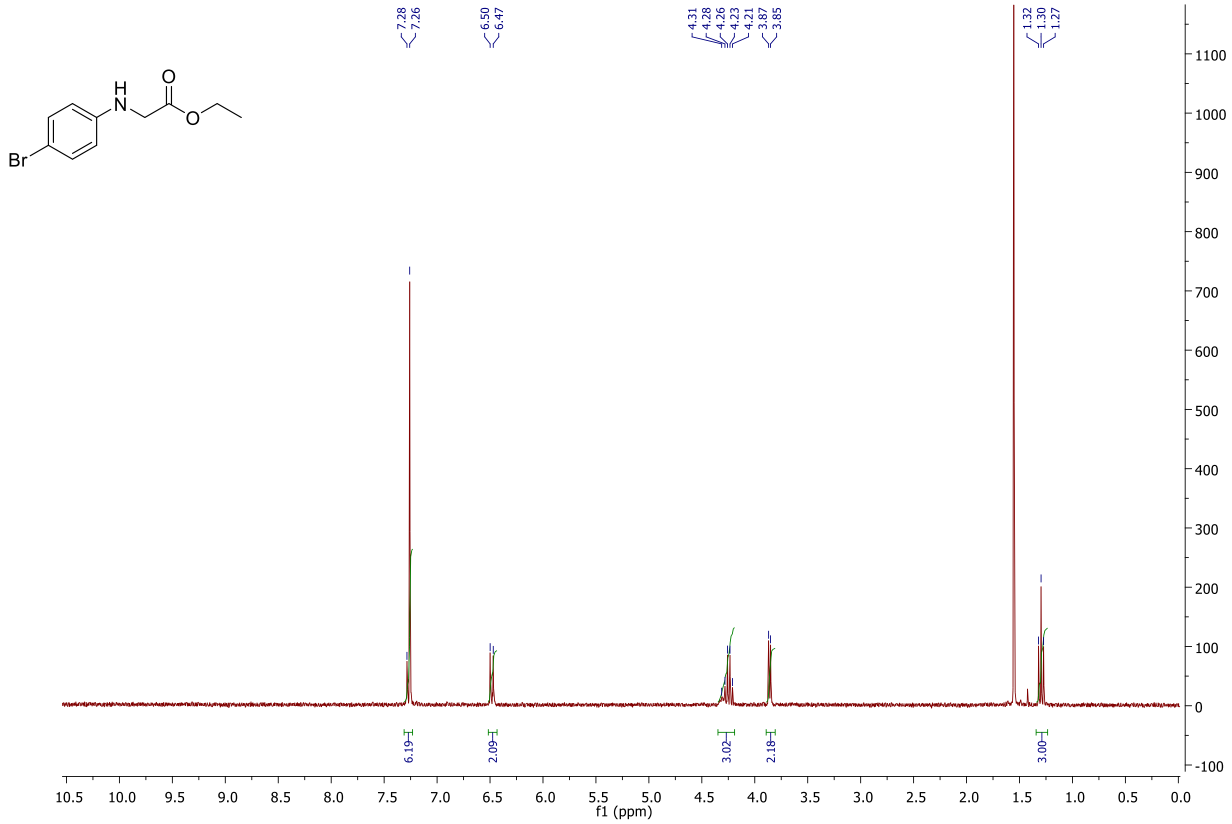

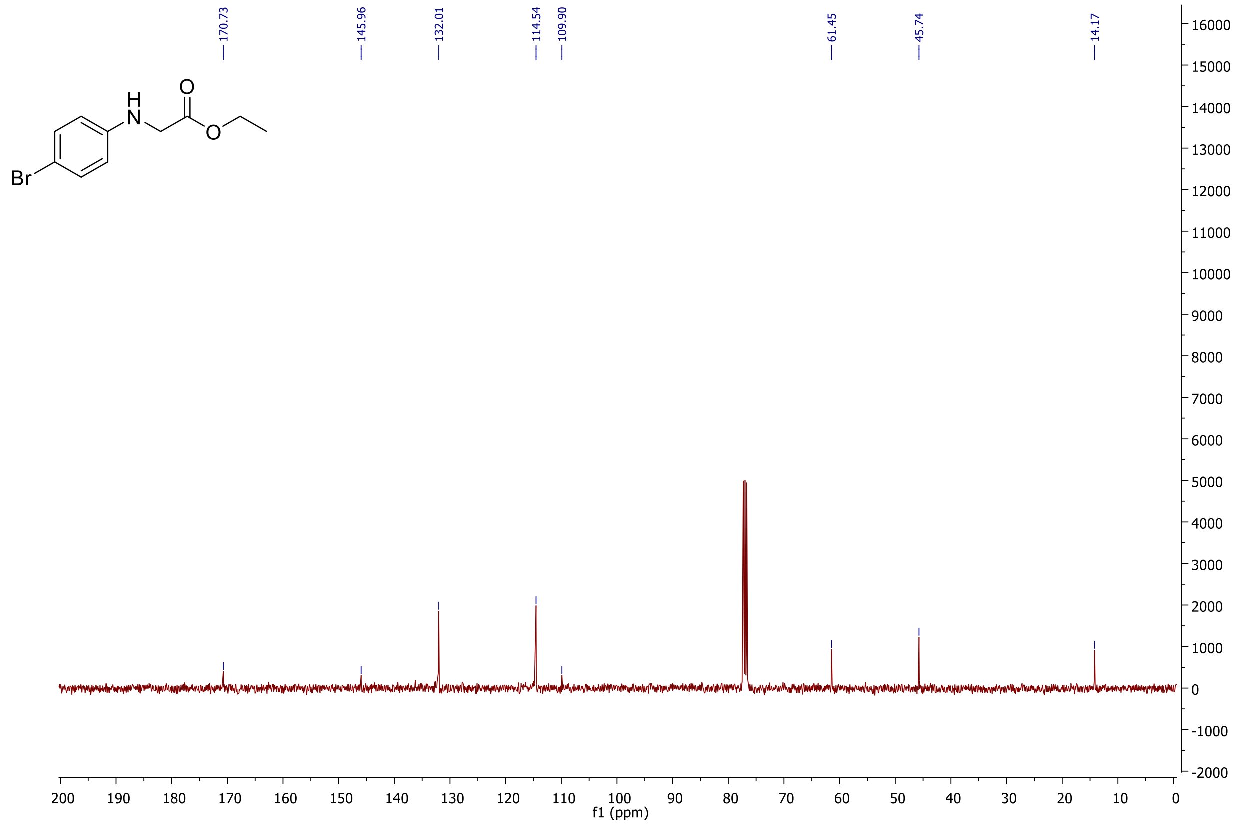

^{1}H-NMR (300 MHz, $CDCl_3$), ^{13}C{^{1}H}-NMR (101 MHz, $CDCl_3$) of *N-(4-Dichlorophenyl)glycine ethyl ester* (1g)

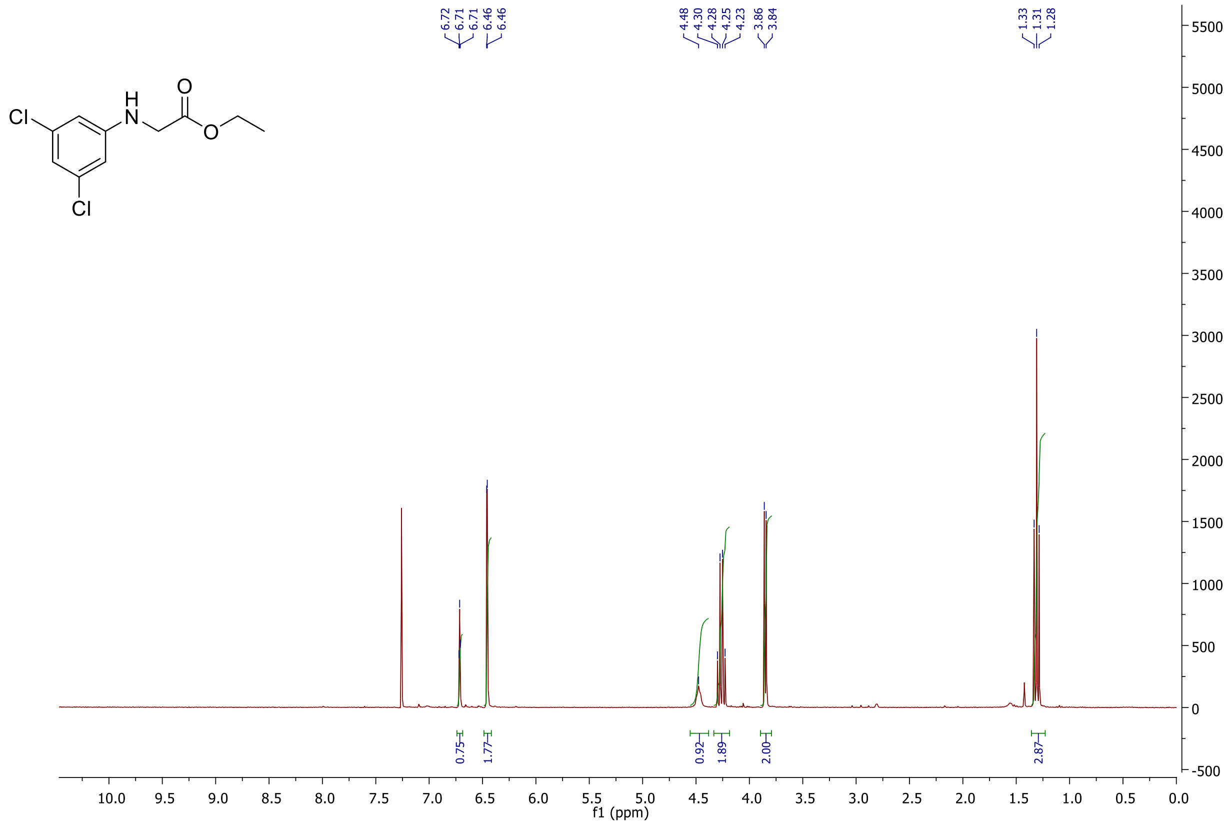

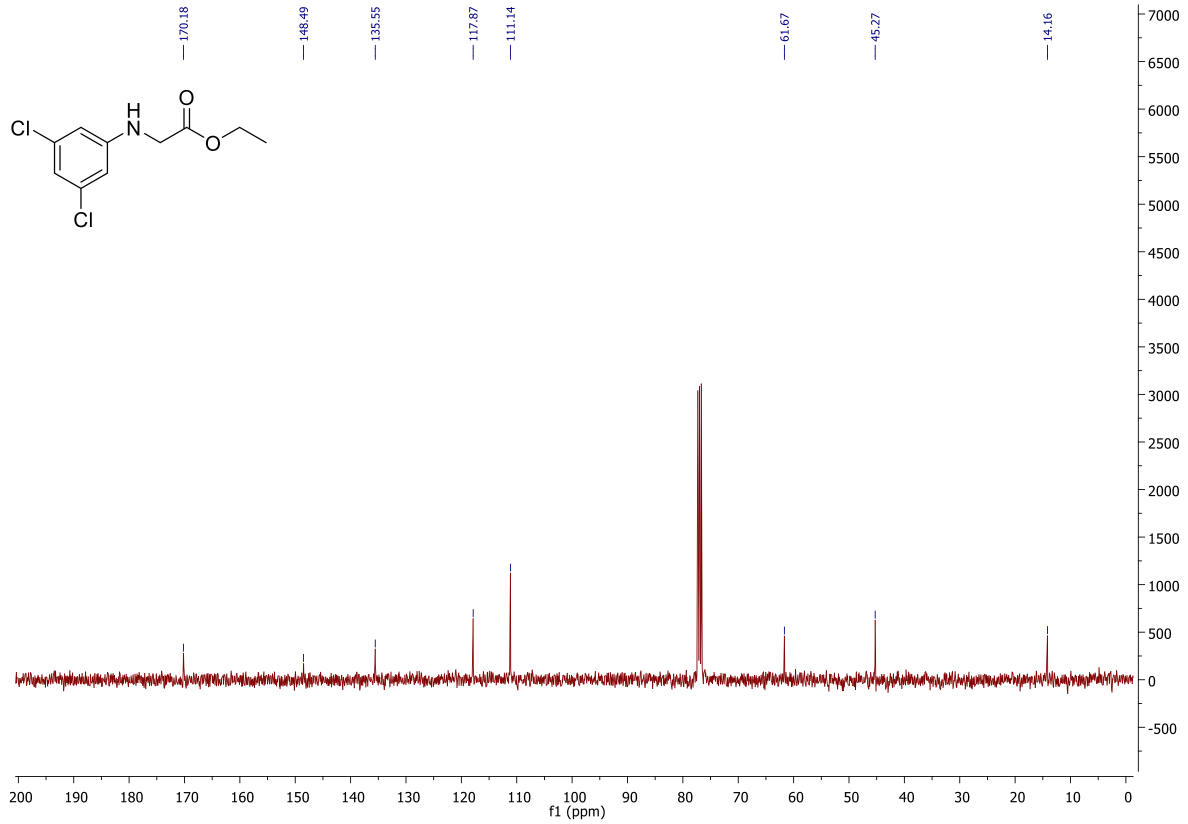

^{1}H-NMR (300 MHz, $CDCl_3$), $^{13}C\{^{1}H\}$-NMR (101 MHz, $CDCl_3$) of *N-(4-Dibromophenyl)glycine ethyl ester* (1h)

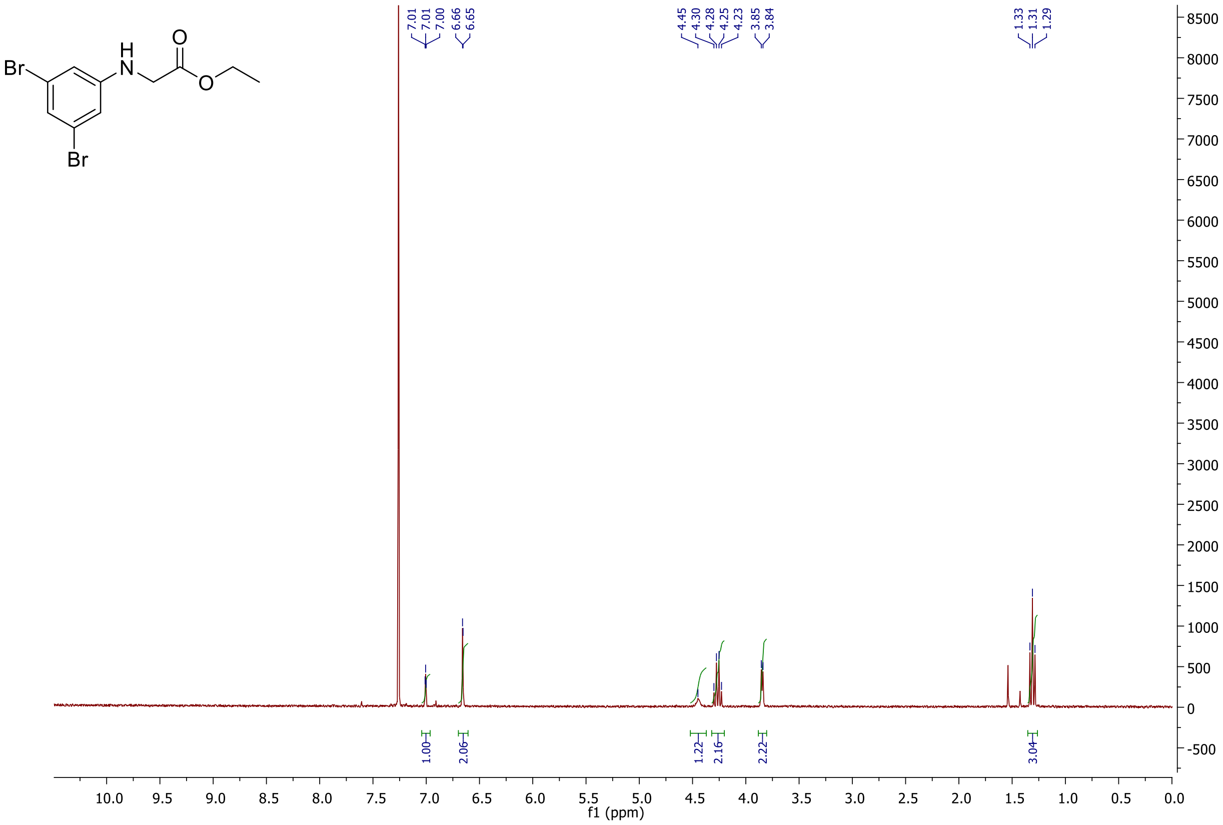

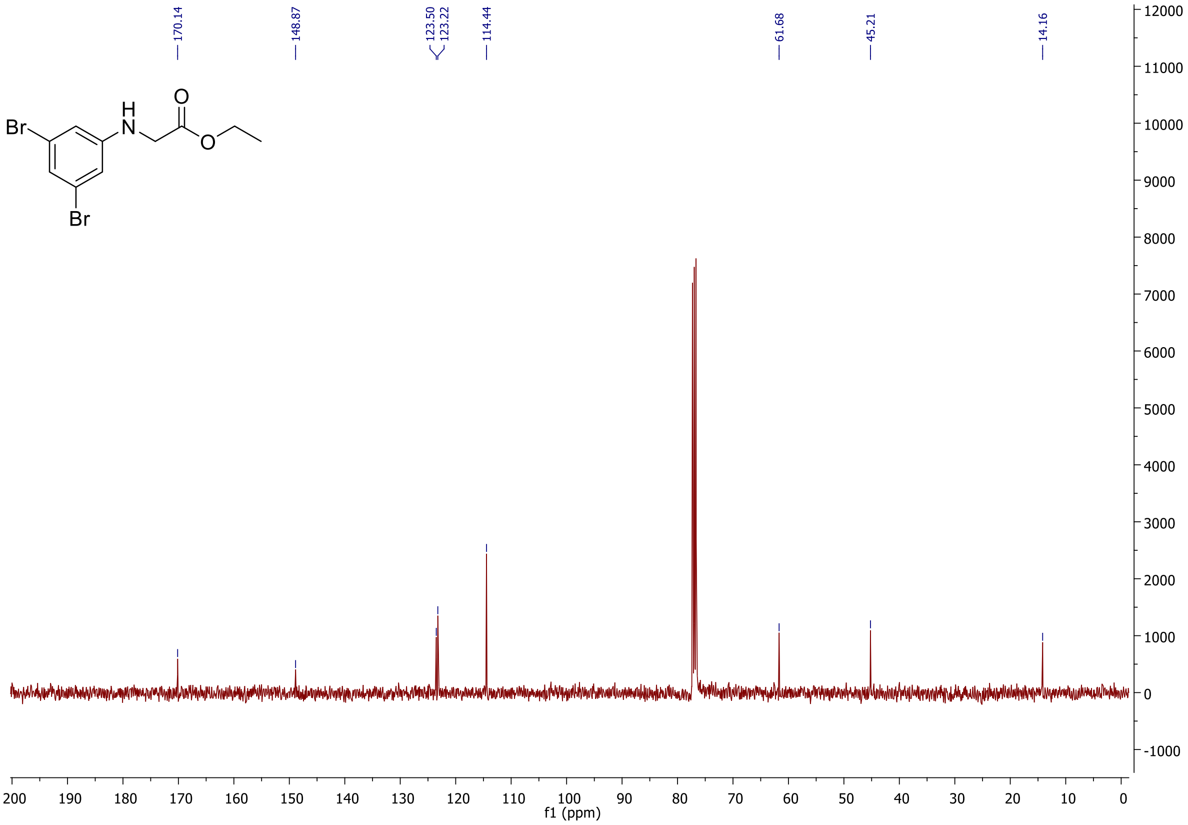

^{1}H-NMR (300 MHz, $CDCl_3$), $^{13}C\{^1H\}$-NMR (101 MHz, $CDCl_3$) of *N-(3-Chlorophenyl)glycine ethyl ester* (1i)

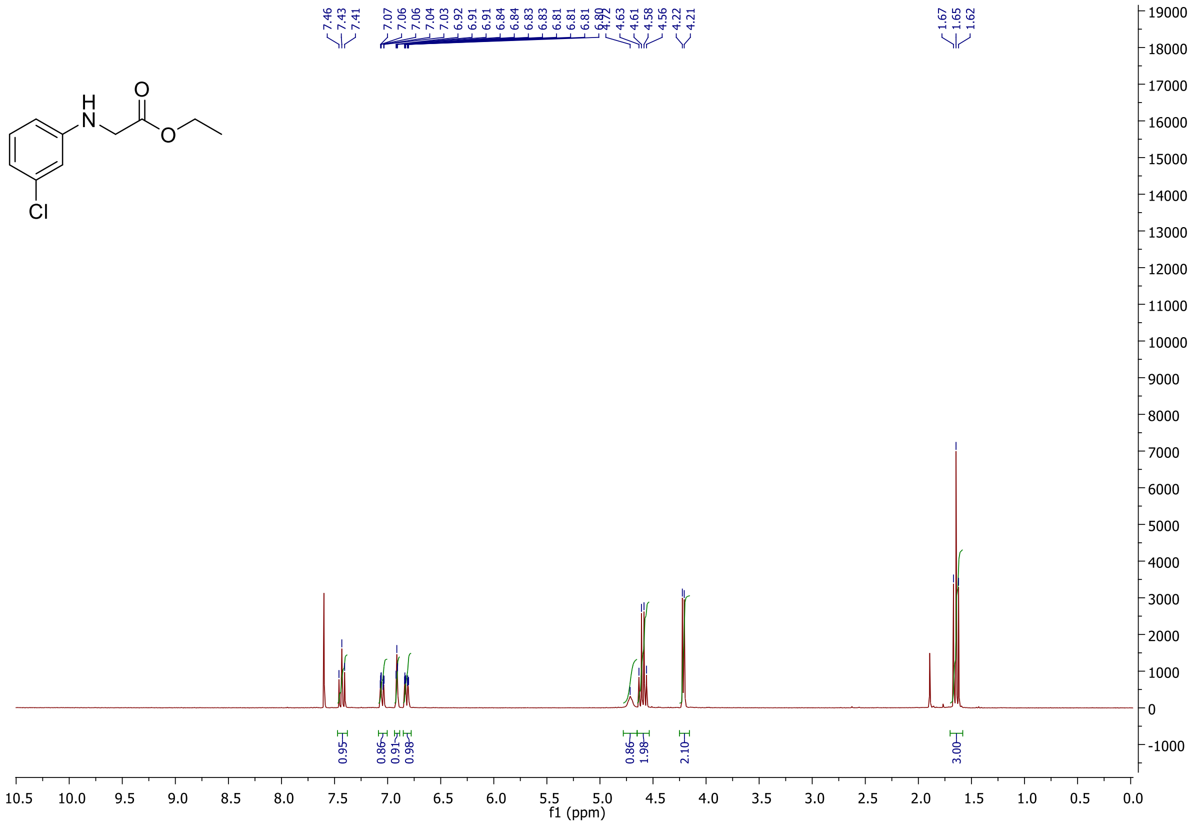

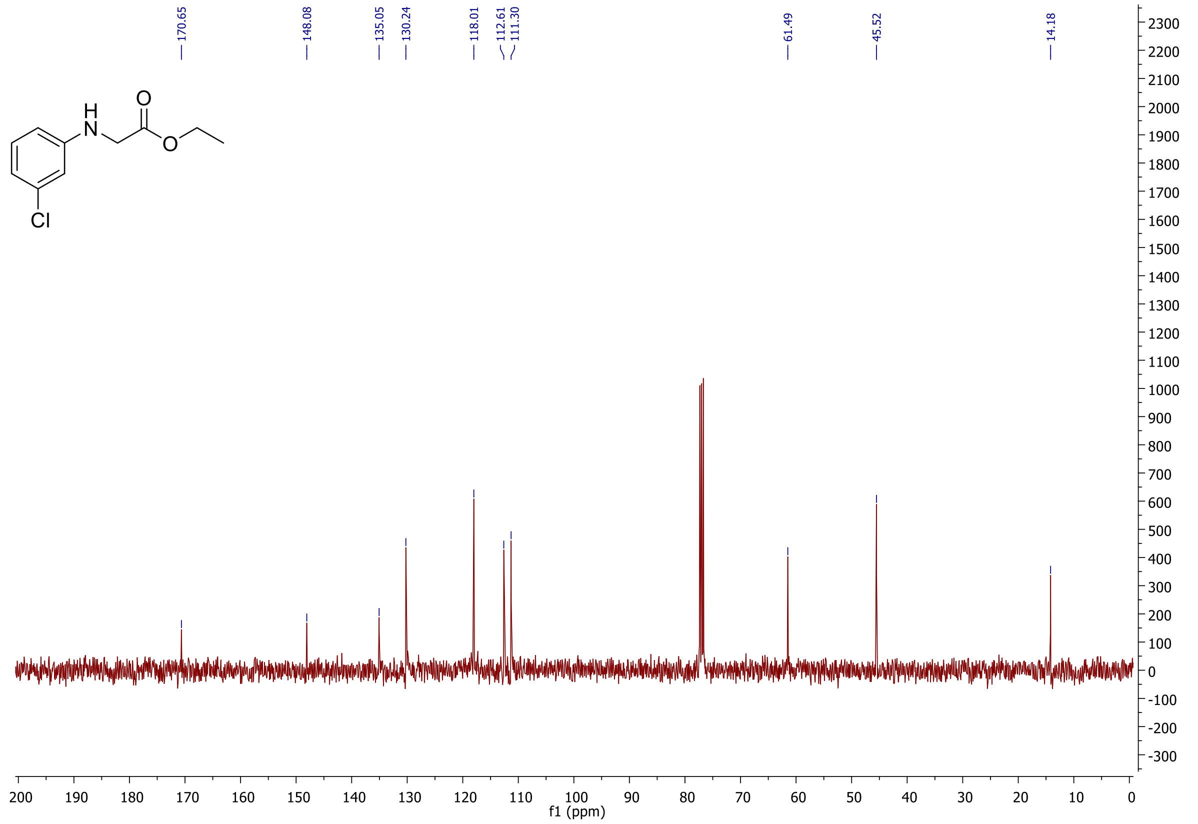

^{1}H-NMR (300 MHz, CDCl$_3$), ^{13}C{^{1}H}-NMR (101 MHz, CDCl$_3$) of *N-(2-Fluorophenyl)glycine ethyl ester* (1j)

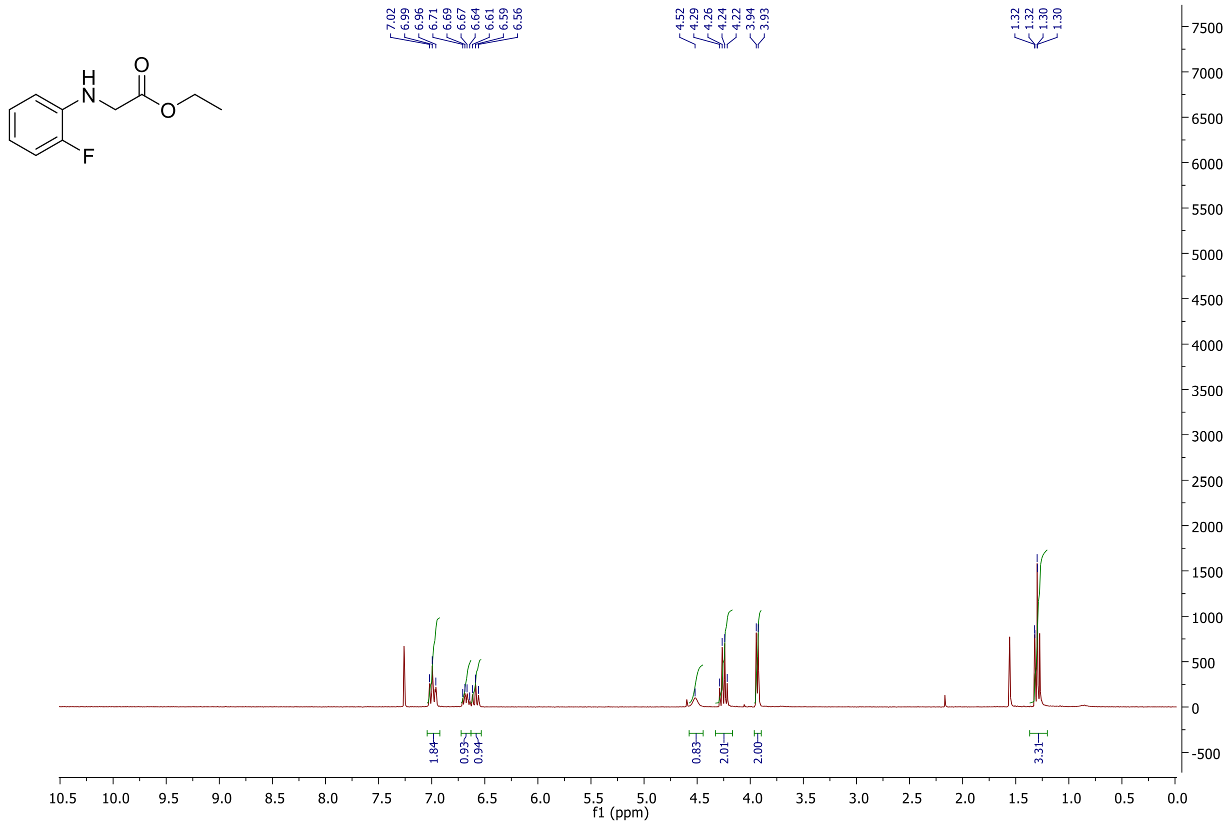

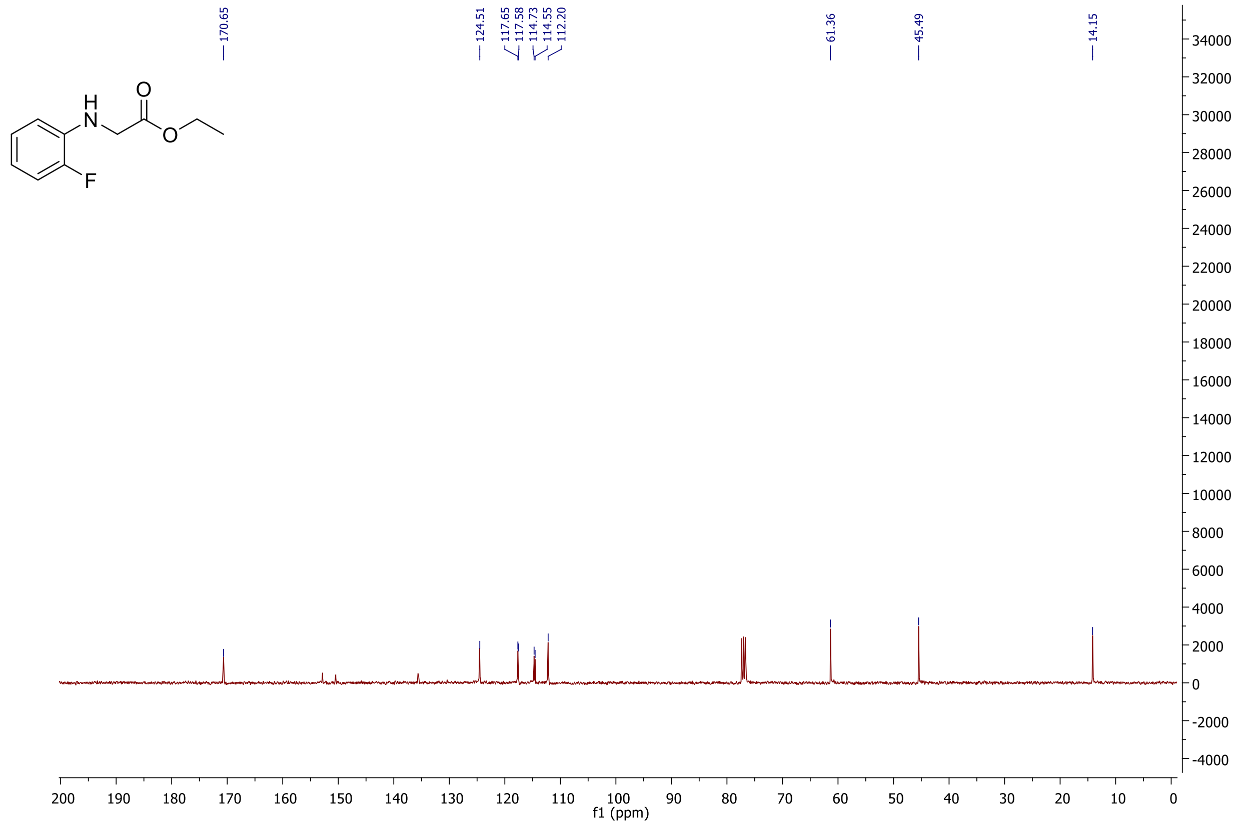

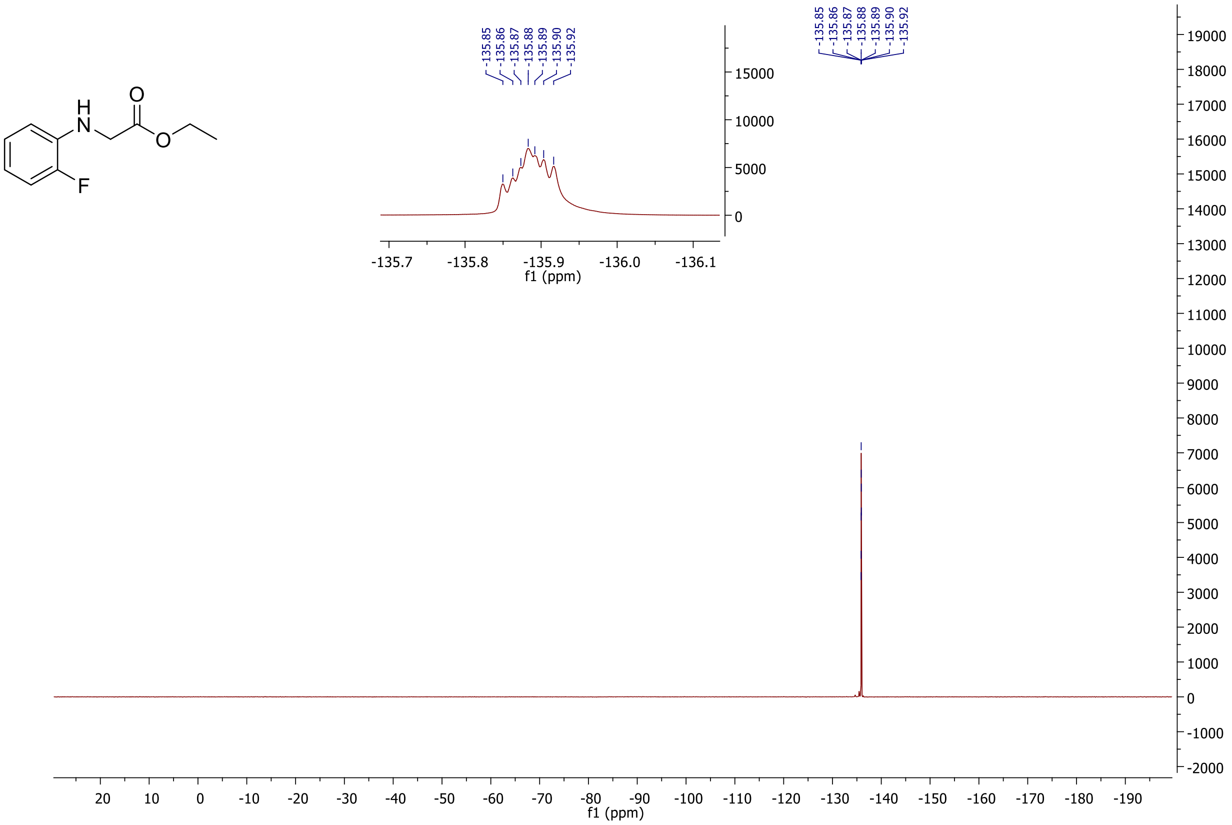
H
N
O
O
F
-135.85
-135.86
-135.87
-135.88
-135.89
-135.90
-135.92
-135.7
-135.8
-135.9
-136.0
-136.1
f1 (ppm)
15000
10000
5000
0
19000
18000
17000
16000
15000
14000
13000
12000
11000
10000
9000
8000
7000
6000
5000
4000
3000
2000
1000
0
-1000
-2000
20
10
0
-10
-20
-30
-40
-50
-60
-70
-80
-90
-100
-110
-120
-130
-140
-150
-160
-170
-180
-190
f1 (ppm)

^{1}H-NMR (300 MHz, $CDCl_3$), ^{13}C{^{1}H}-NMR (101 MHz, $CDCl_3$) of *1-benzyl-1H-indole* (2j)

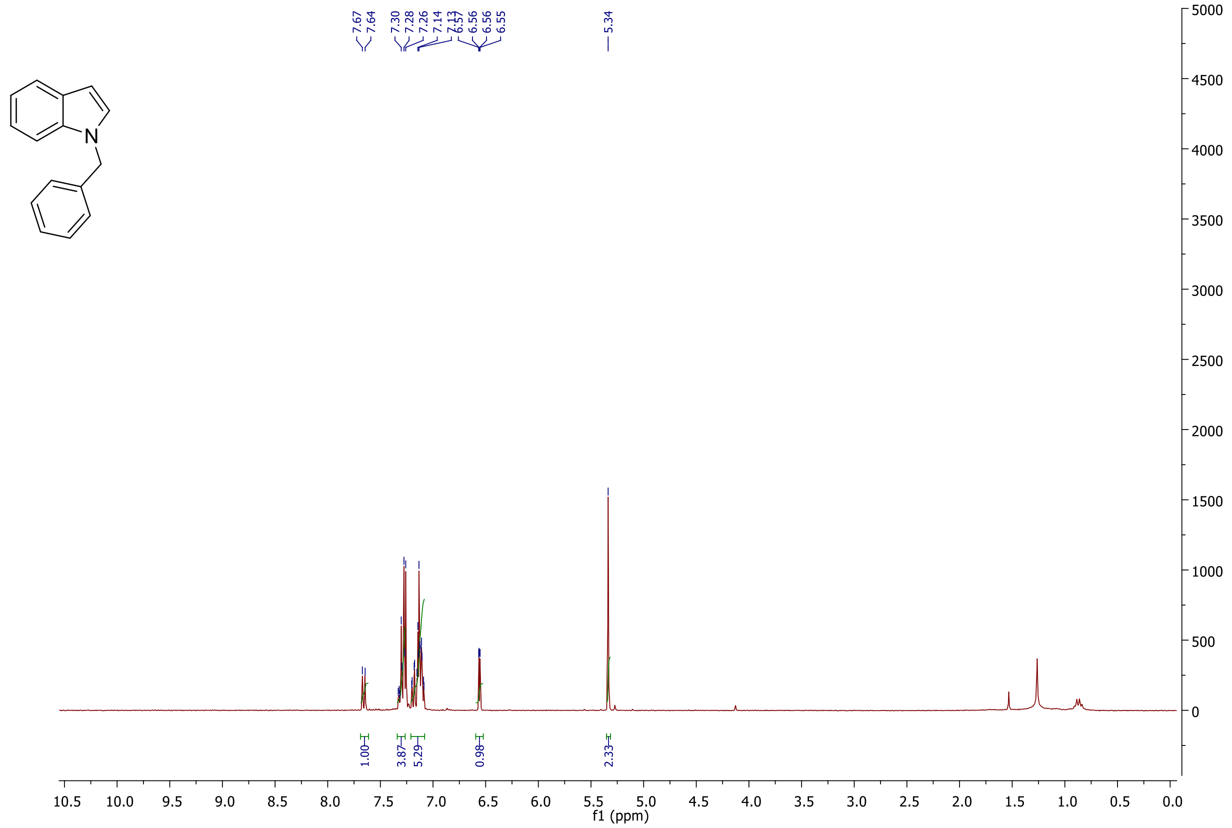

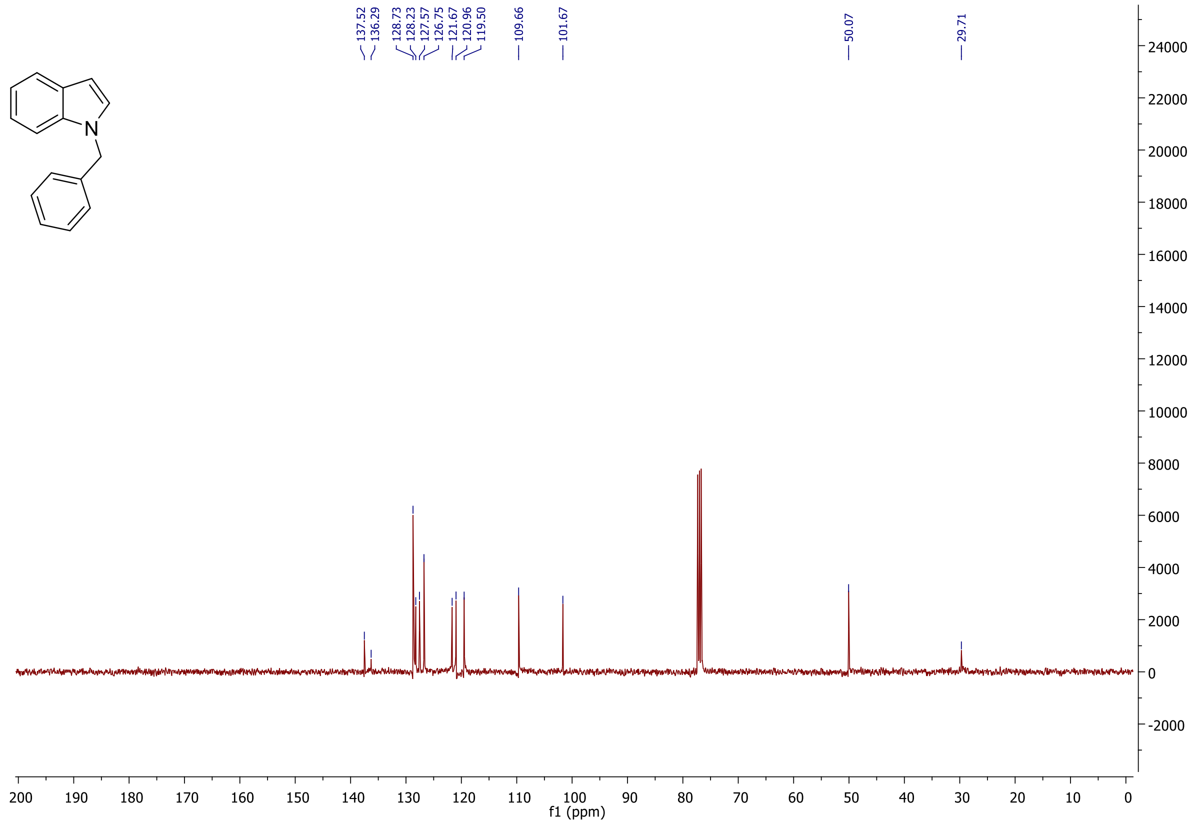

NMR OF PRODUCTS

^{1}H-NMR (300 MHz, $CDCl_3$), ^{13}C{^{1}H}-NMR (101 MHz, $CDCl_3$) of *Ethyl 2-(1H-indol-3-yl)-2-(phenylamino)acetate* (3aa)

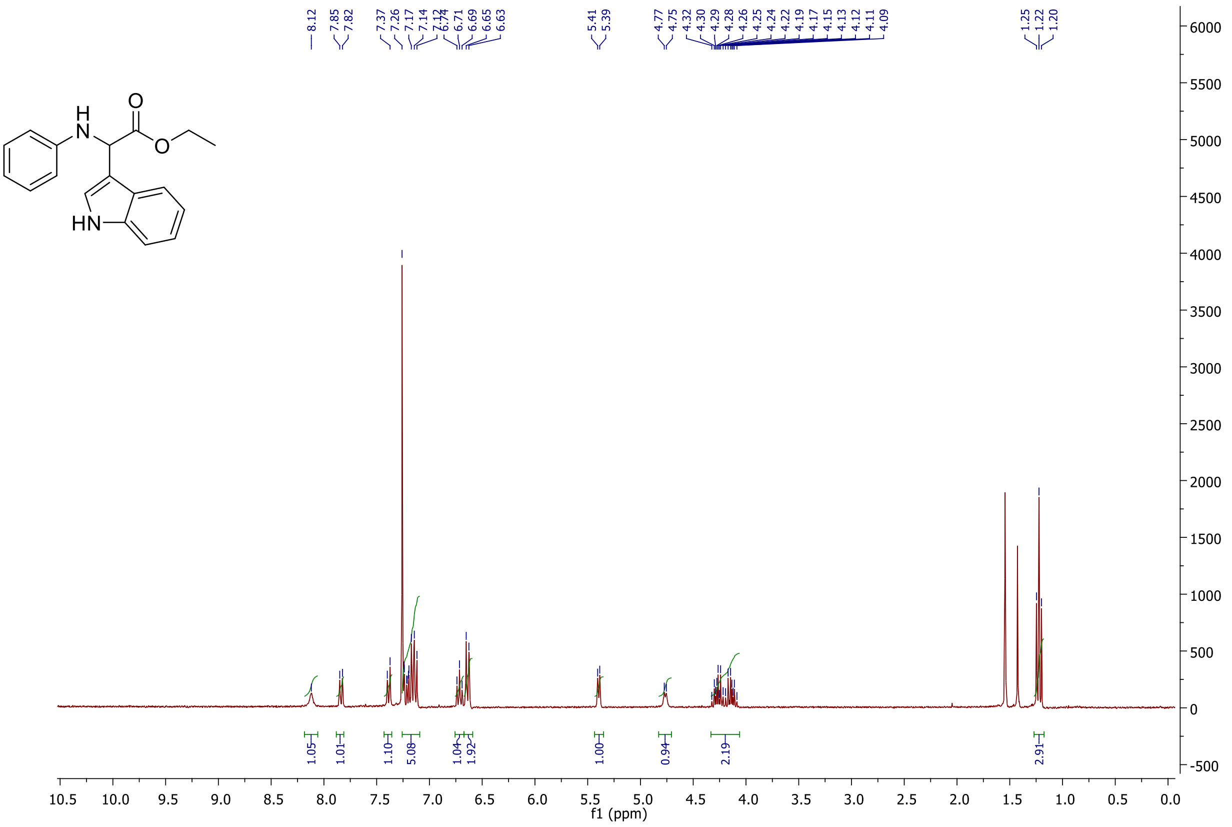

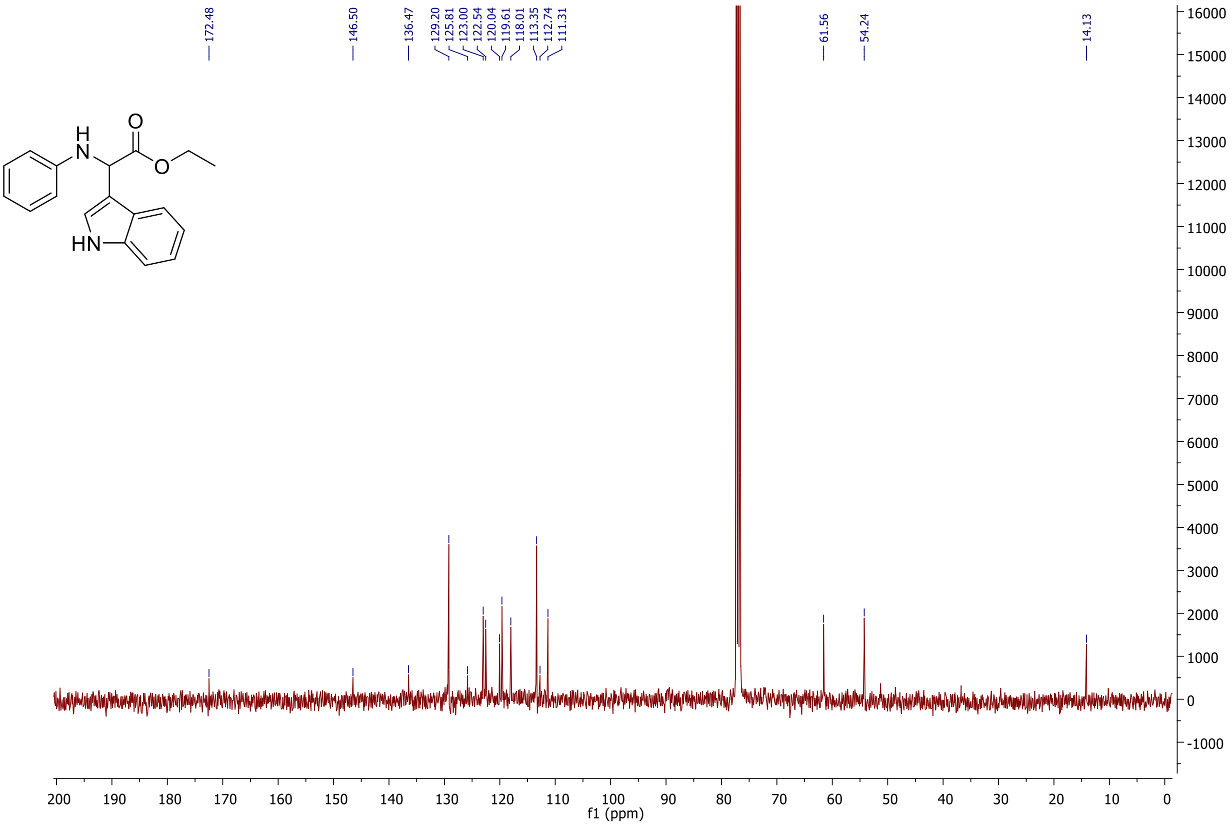

^{1}H-NMR (300 MHz, acetone-D6), ^{13}C{^{1}H}-NMR (101 MHz, acetone-D6) of *Ethyl 2-(1H-indol-3-yl)-2-(p-tolylamino)acetate* (3ba)

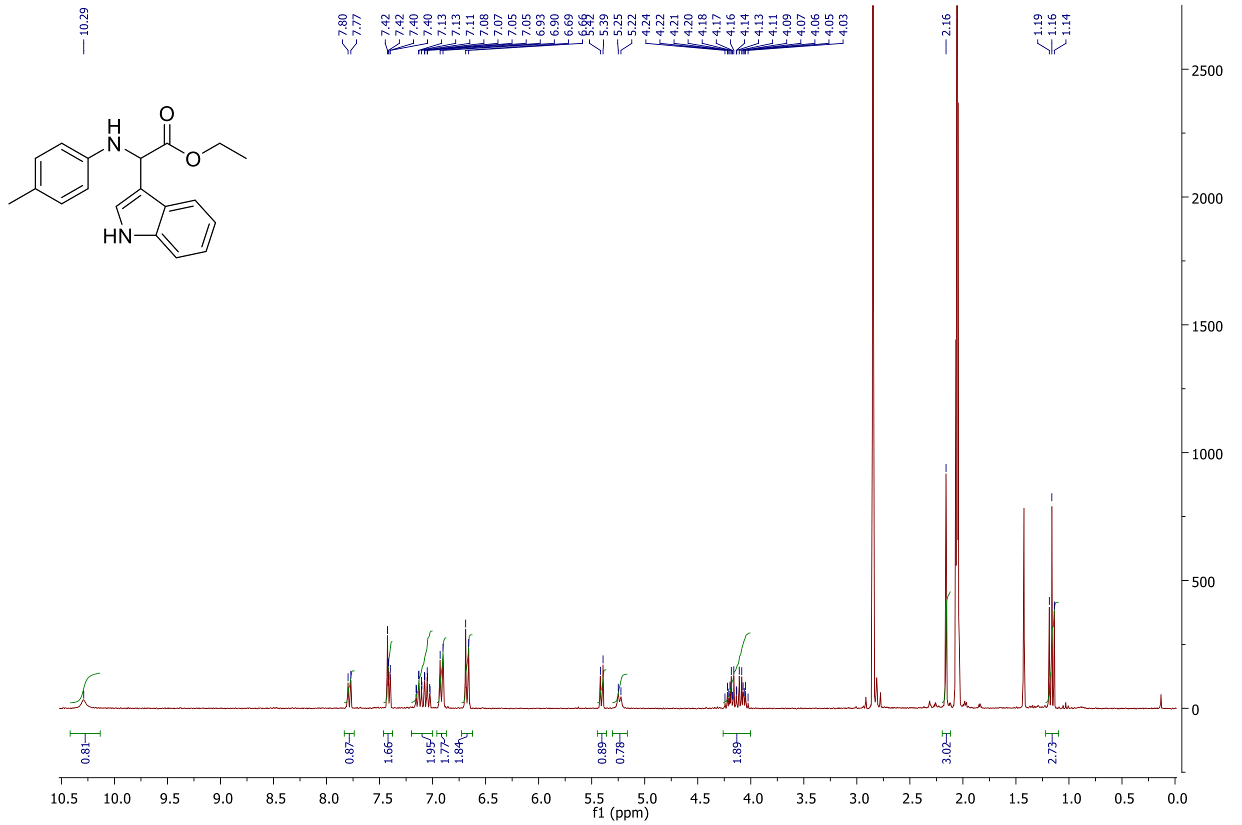

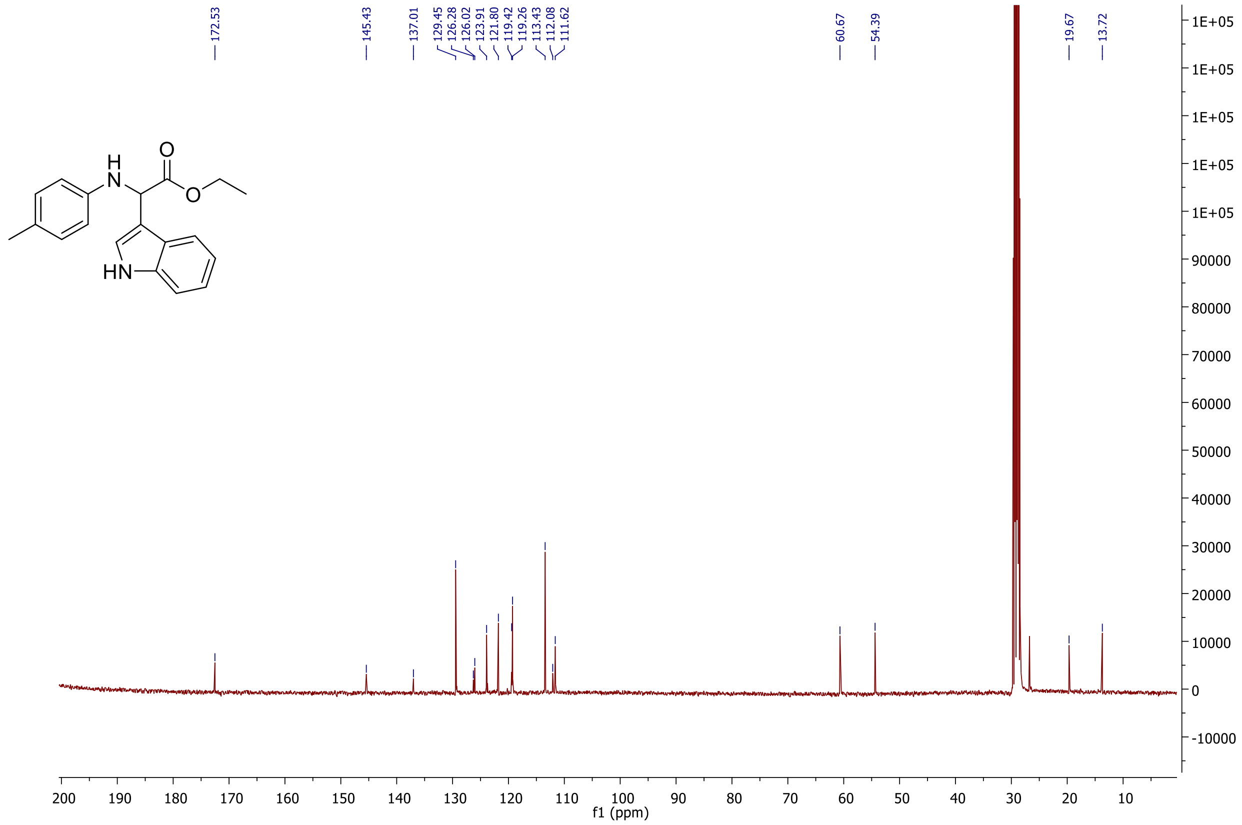

^{1}H-NMR (300 MHz, $CDCl_3$), $^{13}C\{^1H\}$-NMR (101 MHz, $CDCl_3$) of *Ethyl 2-(1H-indol-3-yl)-2-((4-methoxyphenyl)amino)acetate* (3ca)

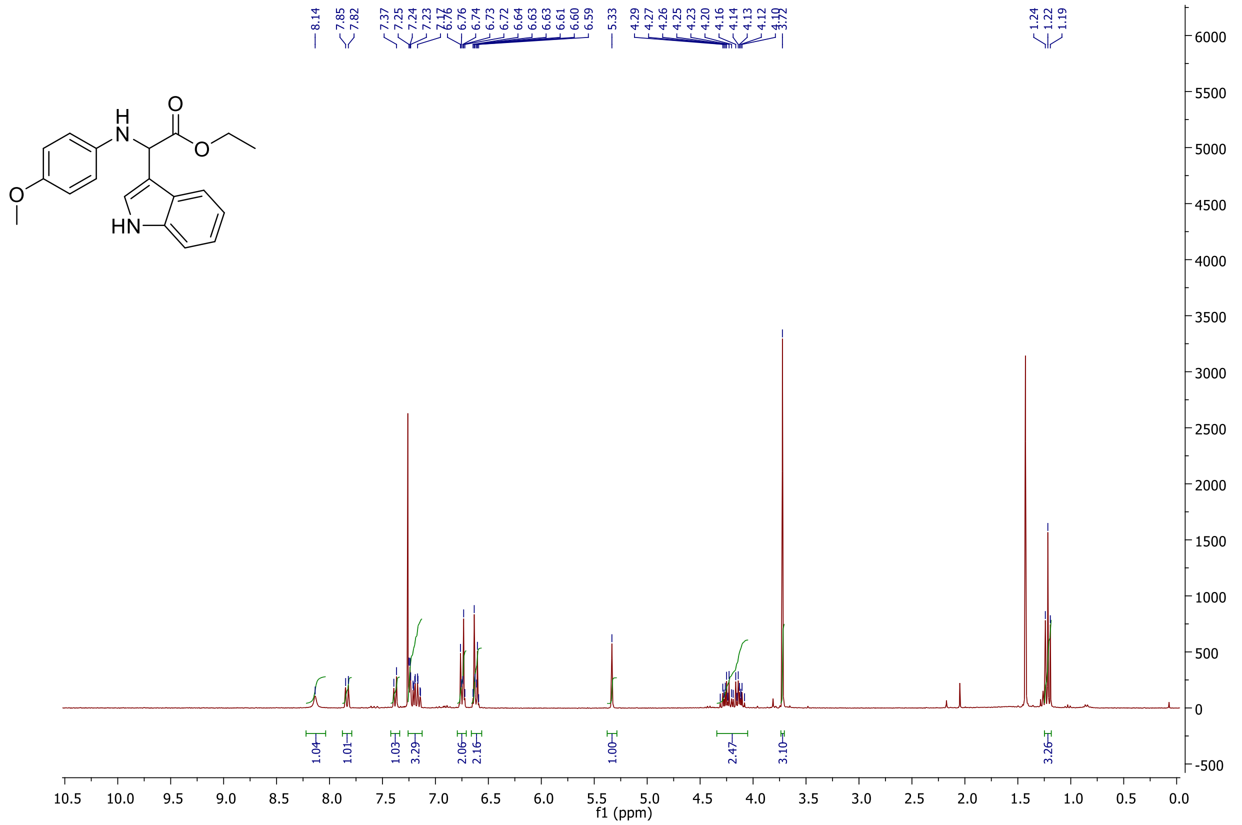

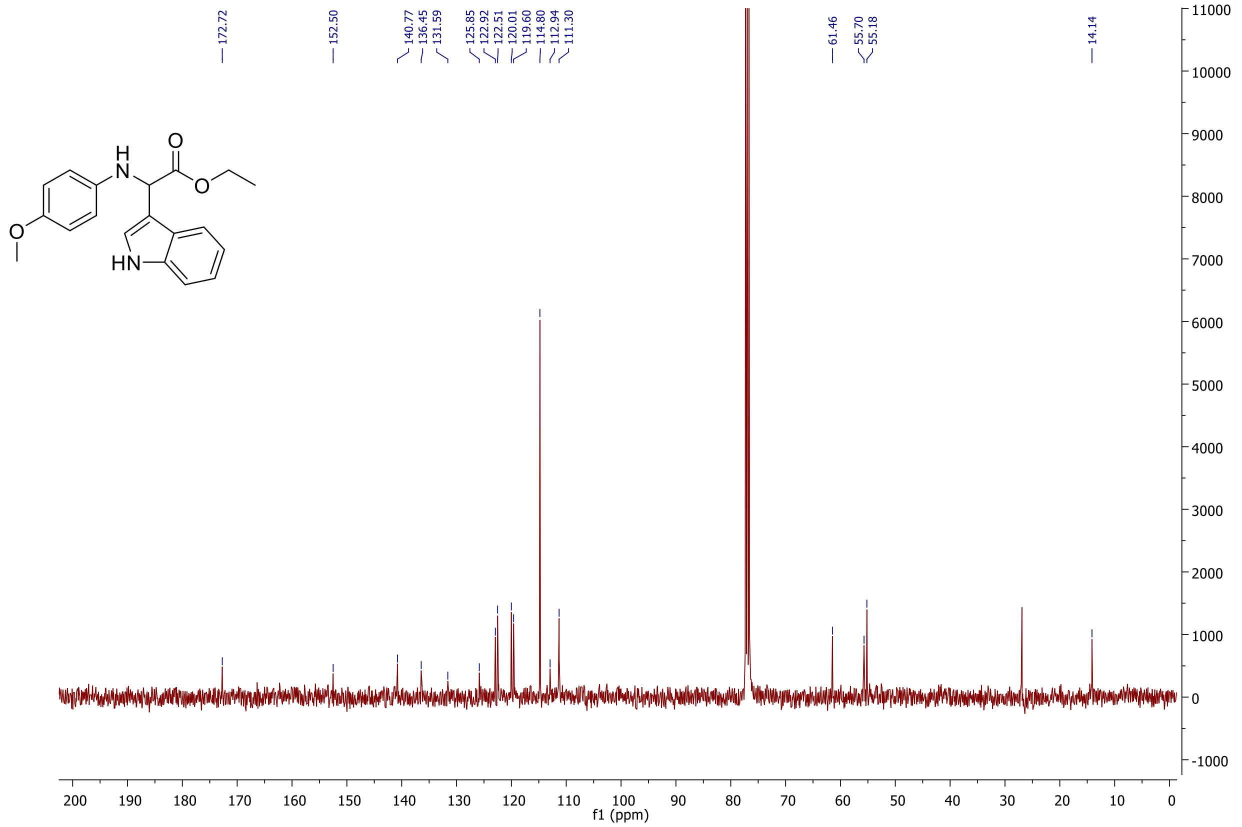

^{1}H-NMR (400 MHz, $CDCl_3$), ^{13}C{^{1}H}-NMR (101 MHz, $CDCl_3$), ^{19}F-NMR (376 MHz, $CDCl_3$) of *Ethyl 2-((4-fluorophenyl)amino)-2-(1H-indol-3-yl)acetate* (3da)

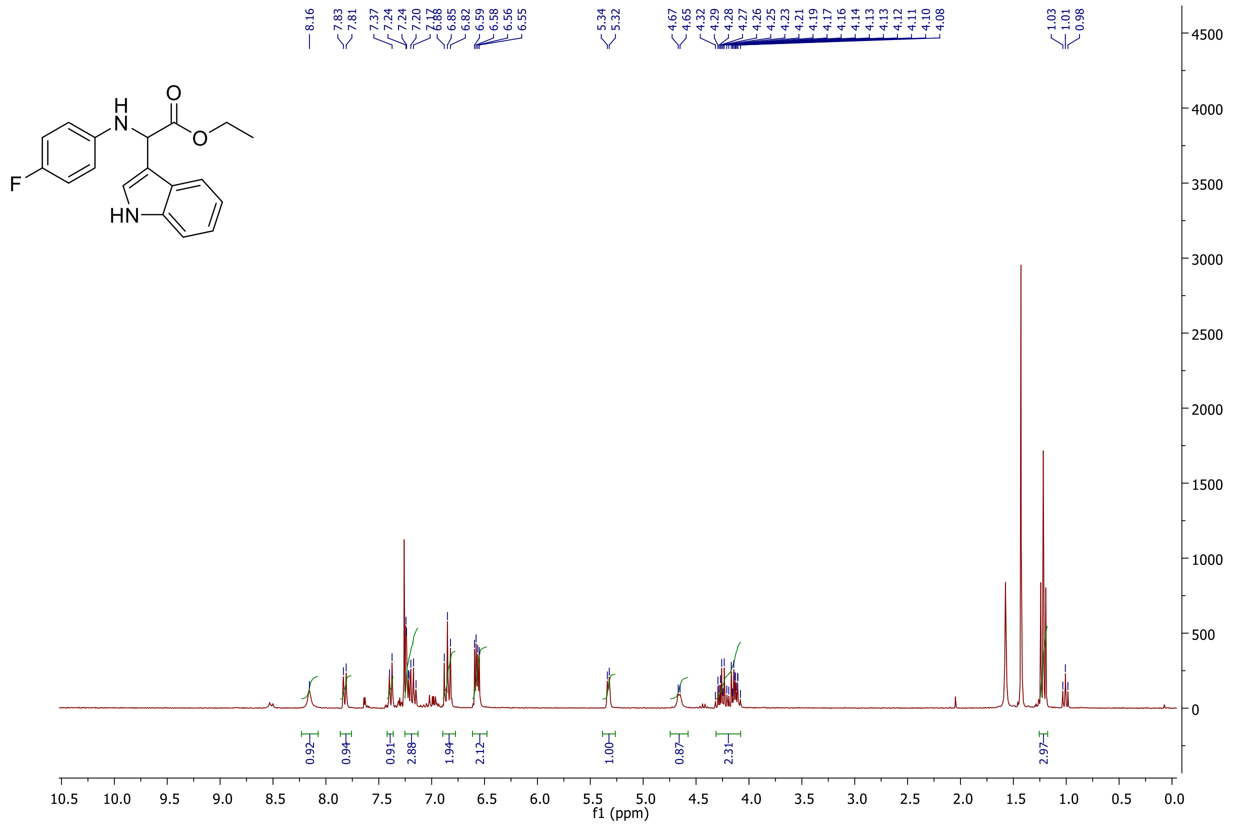

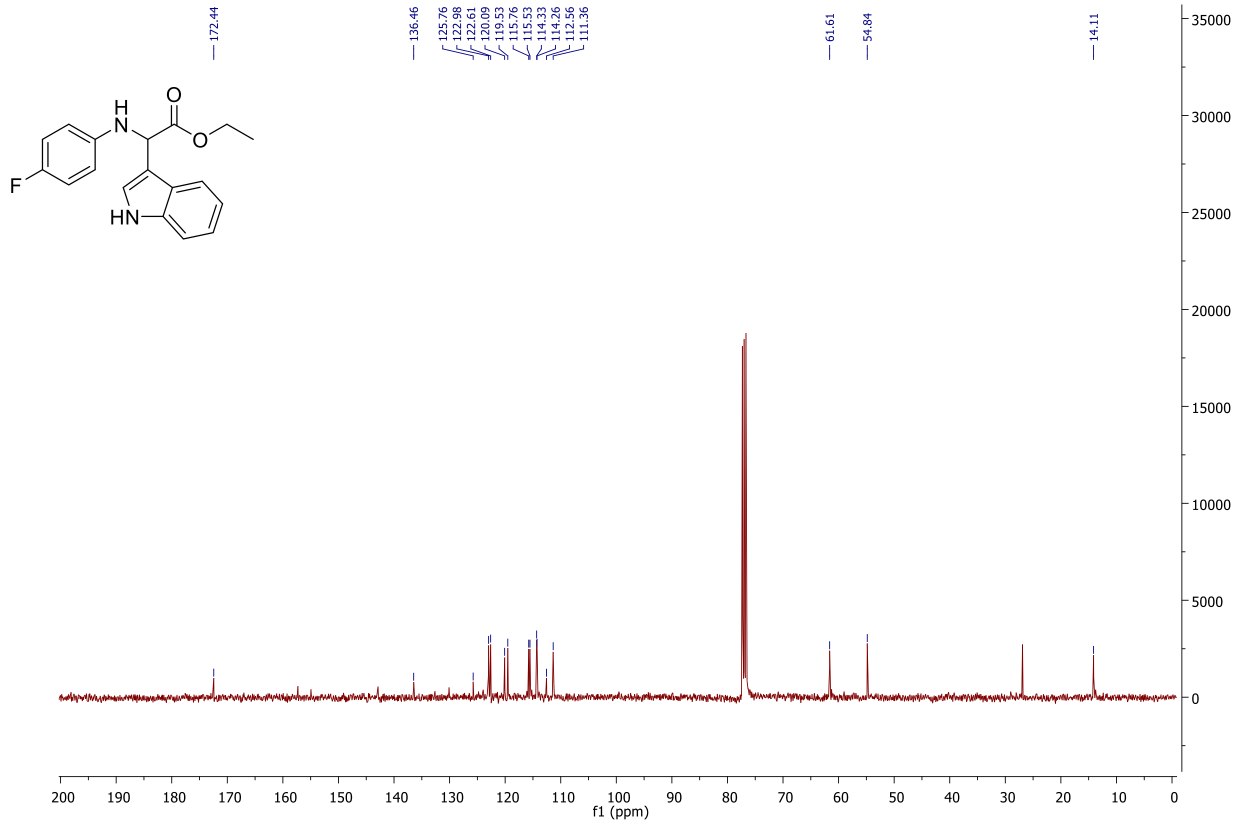

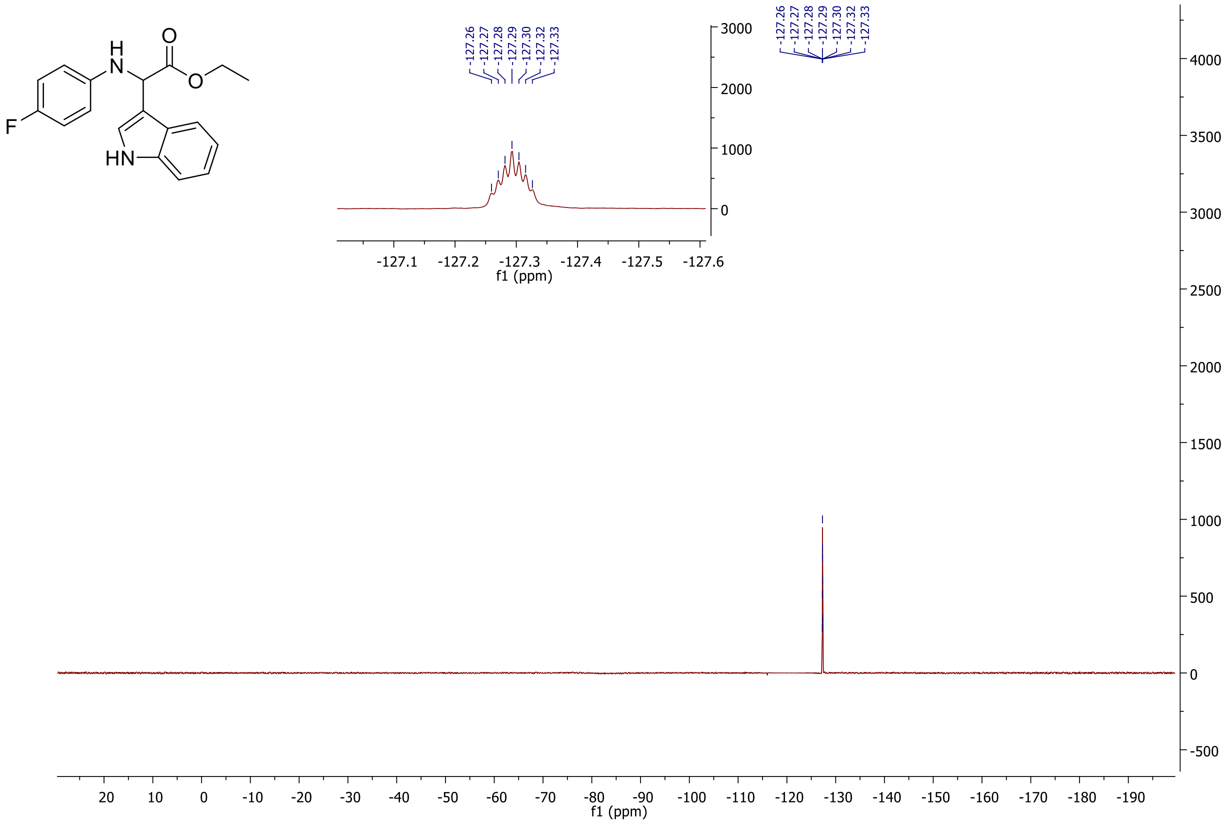
H
N
O
O
F
HN
-127.26
-127.27
-127.28
-127.29
-127.30
-127.32
-127.33
-127.1 -127.2 -127.3 -127.4 -127.5 -127.6
f1 (ppm)
3000
2000
1000
0
4000
3500
3000
2500
2000
1500
1000
500
0
-500
20 10 0 -10 -20 -30 -40 -50 -60 -70 -80 -90 -100 -110 -120 -130 -140 -150 -160 -170 -180 -190
f1 (ppm)

^{1}H-NMR (400 MHz, acetone-D6), ^{13}C{^{1}H}-NMR (101 MHz, acetone-D6) of *Ethyl 2-((4-chlorophenyl)amino)-2-(1H-indol-3-yl)acetate* (3ea)

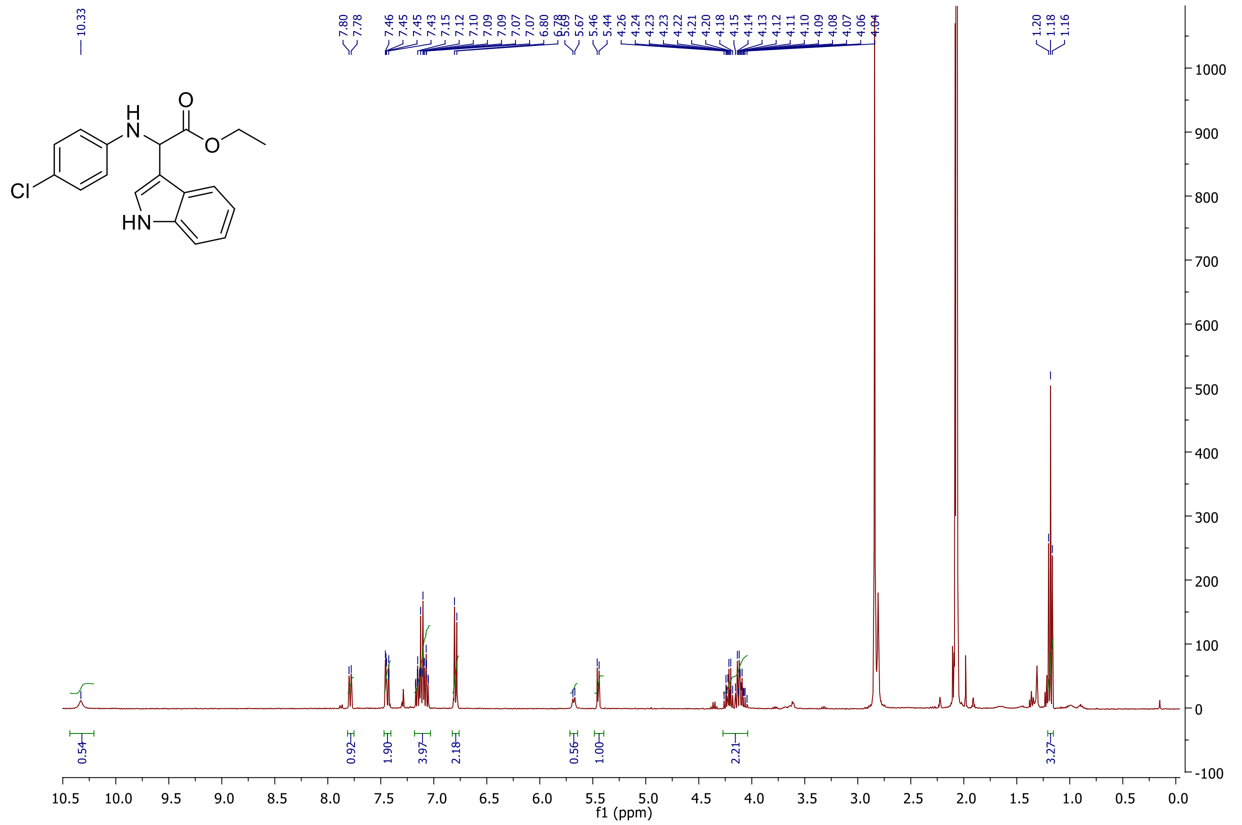

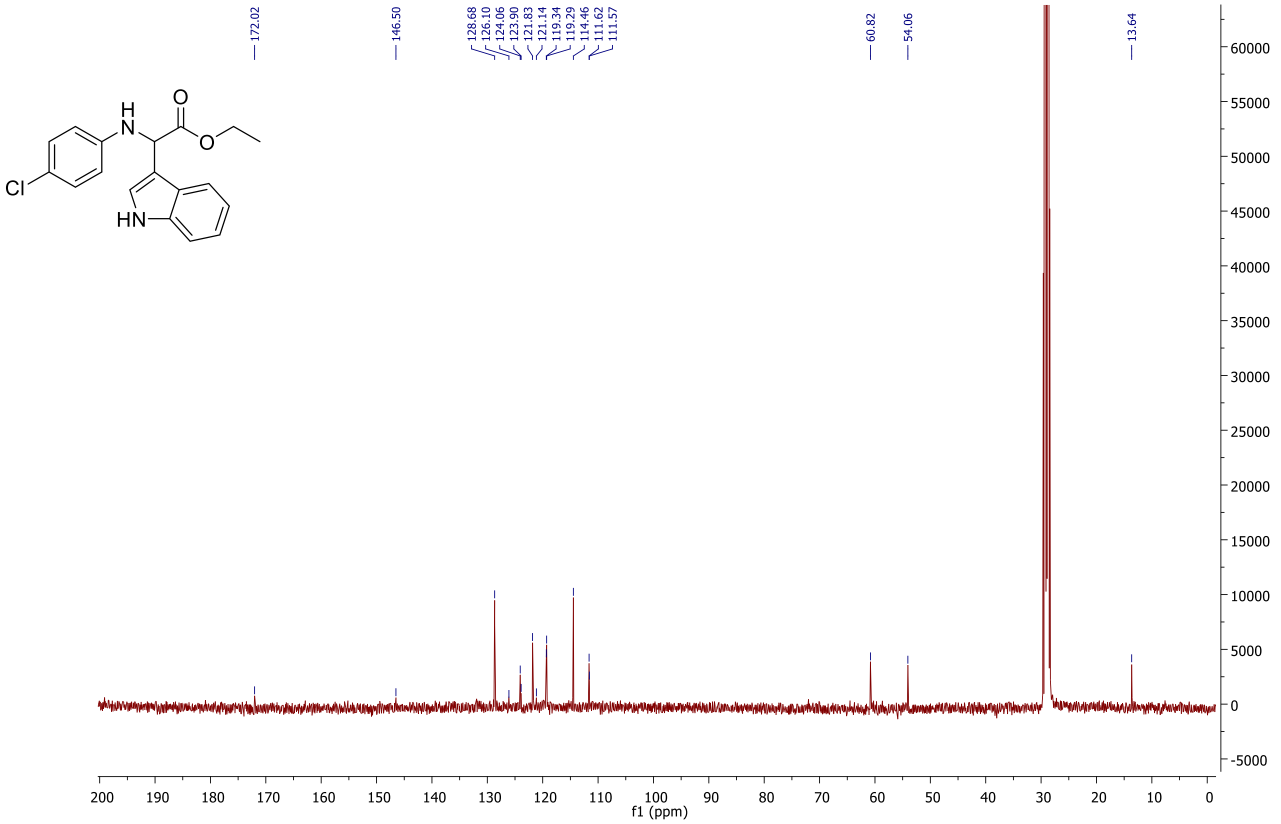

^{1}H-NMR (300 MHz, $CDCl_3$), $^{13}C\{^1H\}$-NMR (101 MHz, $CDCl_3$) of *Ethyl 2-((4-bromophenyl)amino)-2-(1H-indol-3-yl)acetate* (3fa)

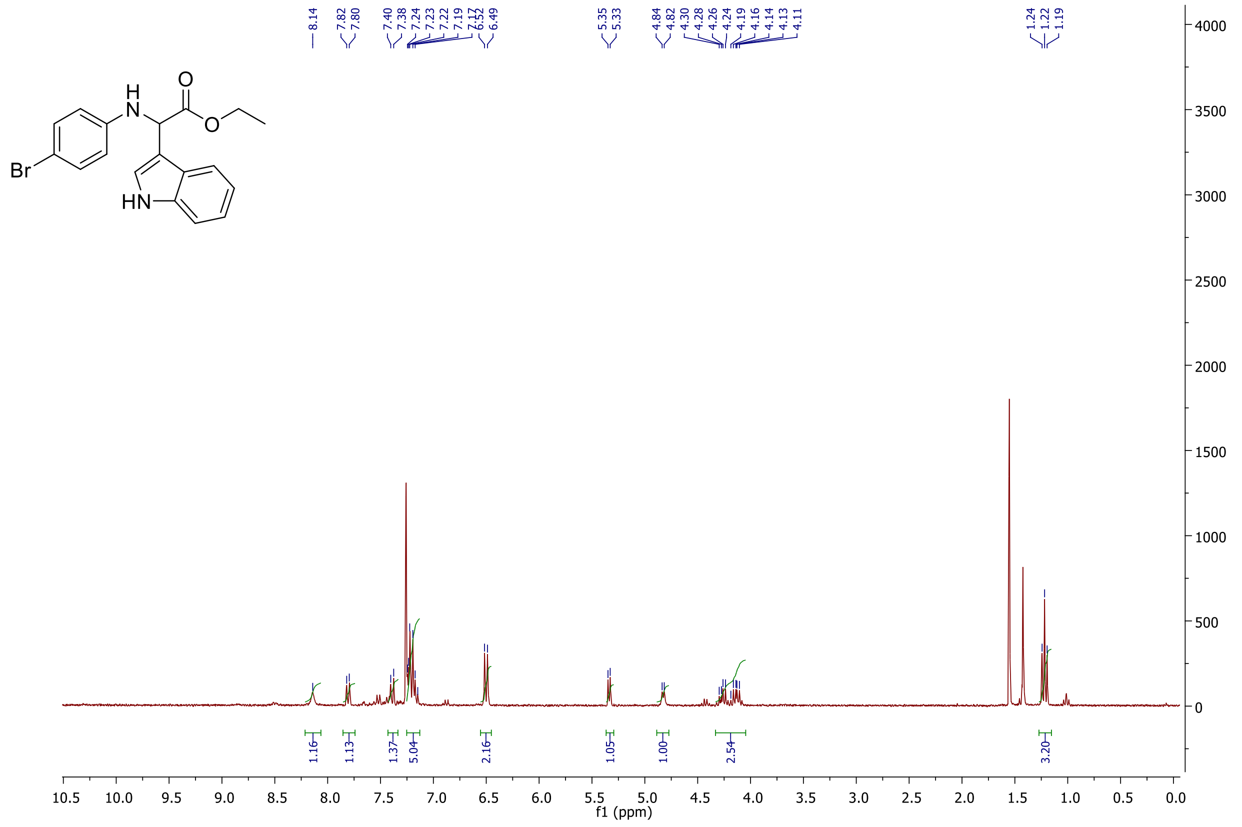

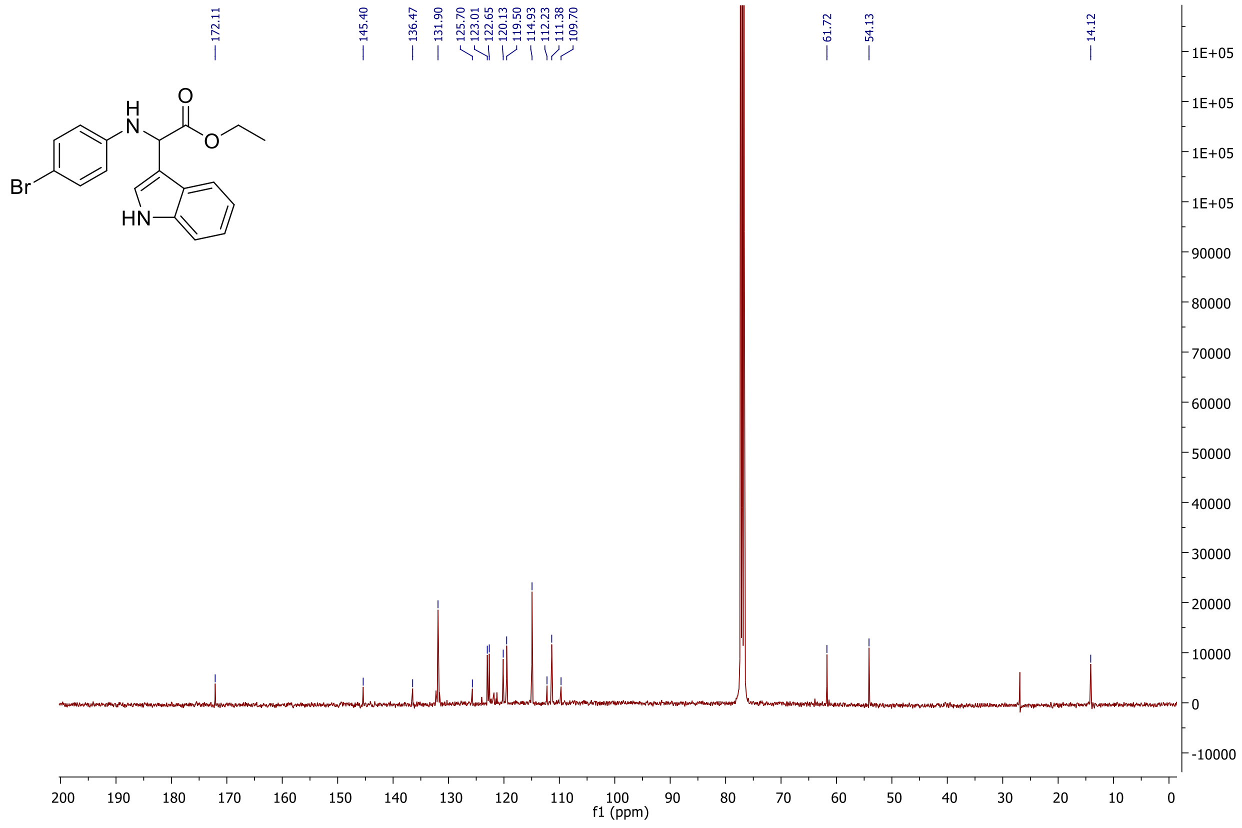

^{1}H-NMR (300 MHz, $CDCl_3$), ^{13}C{^{1}H}-NMR (101 MHz, $CDCl_3$) of *Ethyl 2-((3-chlorophenyl)amino)-2-(1H-indol-3-yl)acetate* (3ia)

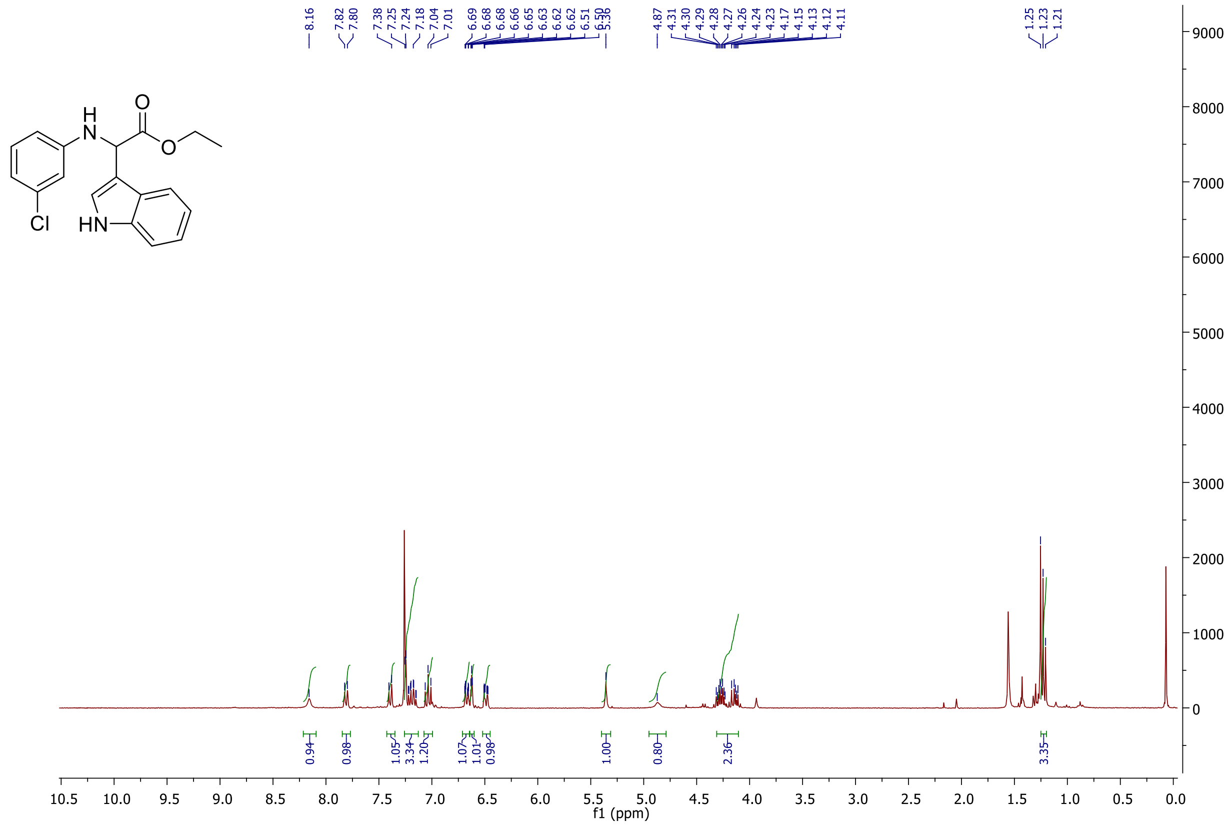

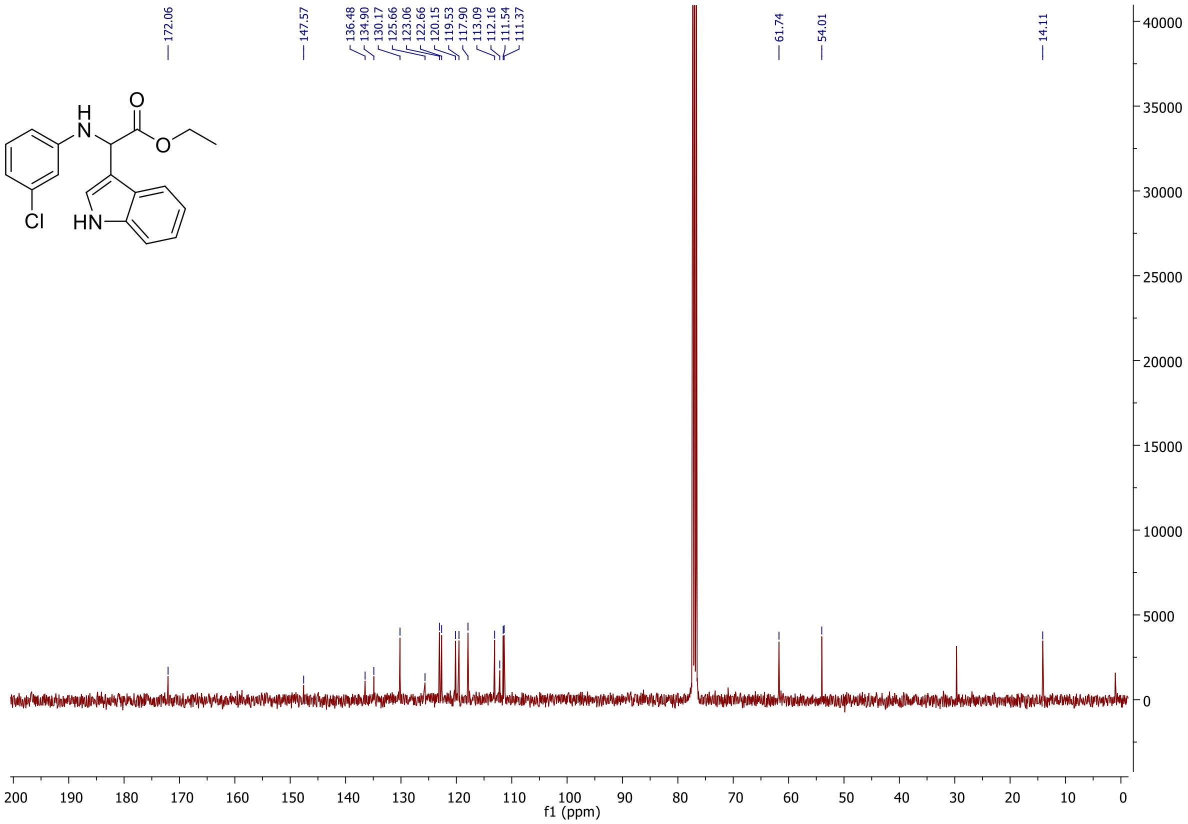

^{1}H-NMR (300 MHz, $CDCl_3$), $^{13}C\{^1H\}$-NMR (101 MHz, $CDCl_3$), ^{19}F-NMR (376 MHz, $CDCl_3$) of *Ethyl 2-((2-fluorophenyl)amino)-2-(1H-indol-3-yl)acetate* (3ja)

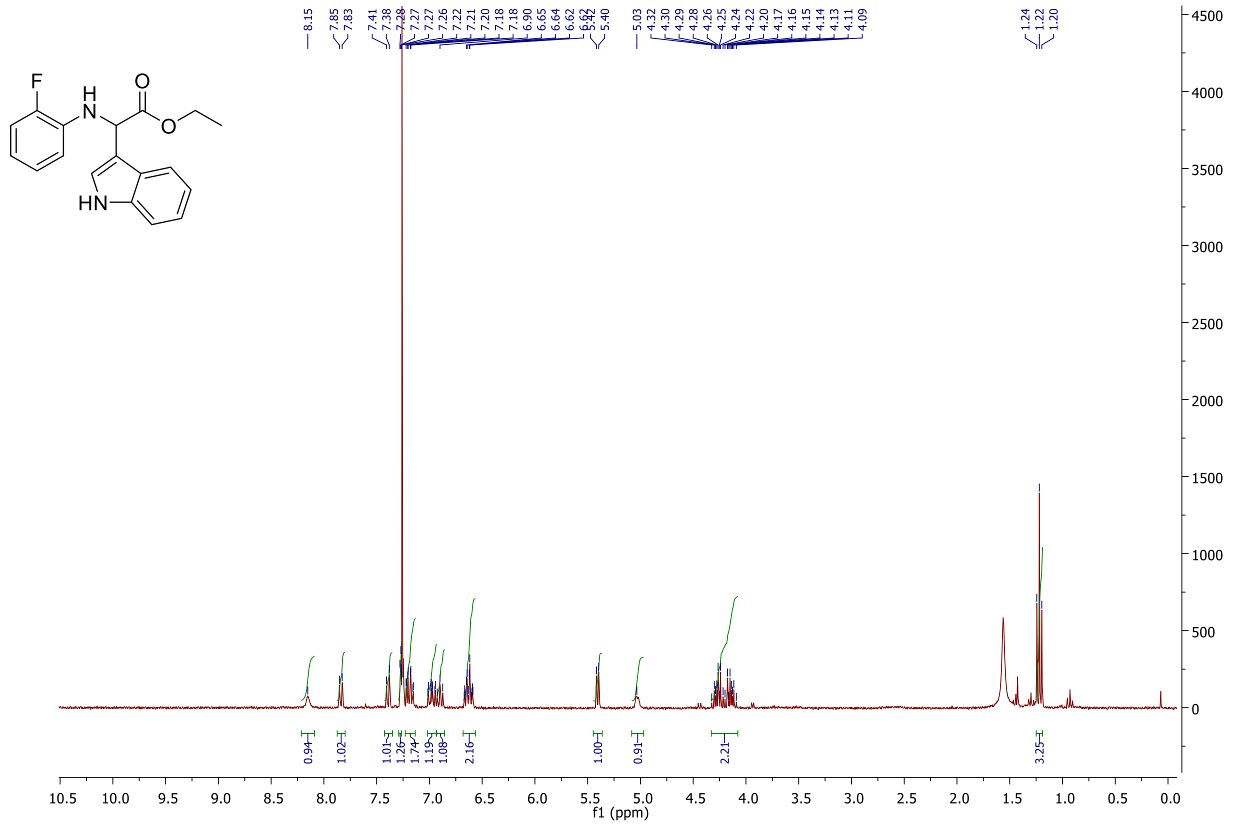

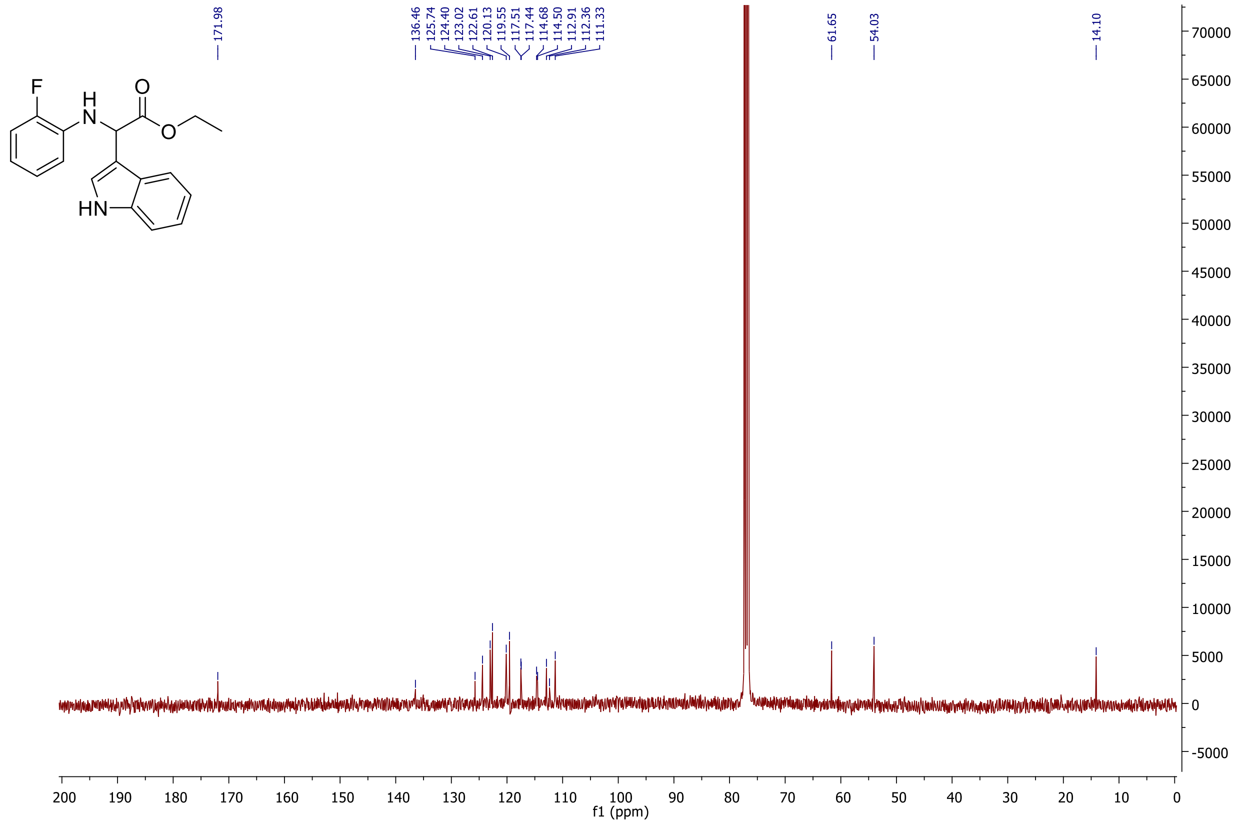

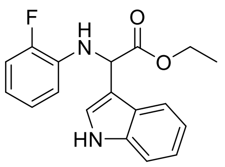

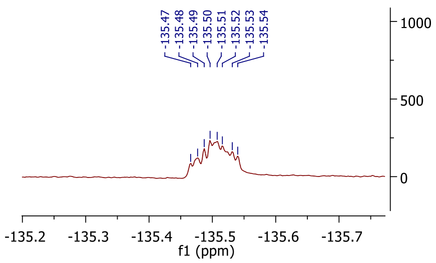
-135.47
-135.48
-135.49
-135.50
-135.51
-135.52
-135.53
-135.54
1000
500
0
-135.2 -135.3 -135.4 -135.5 -135.6 -135.7
f1 (ppm)

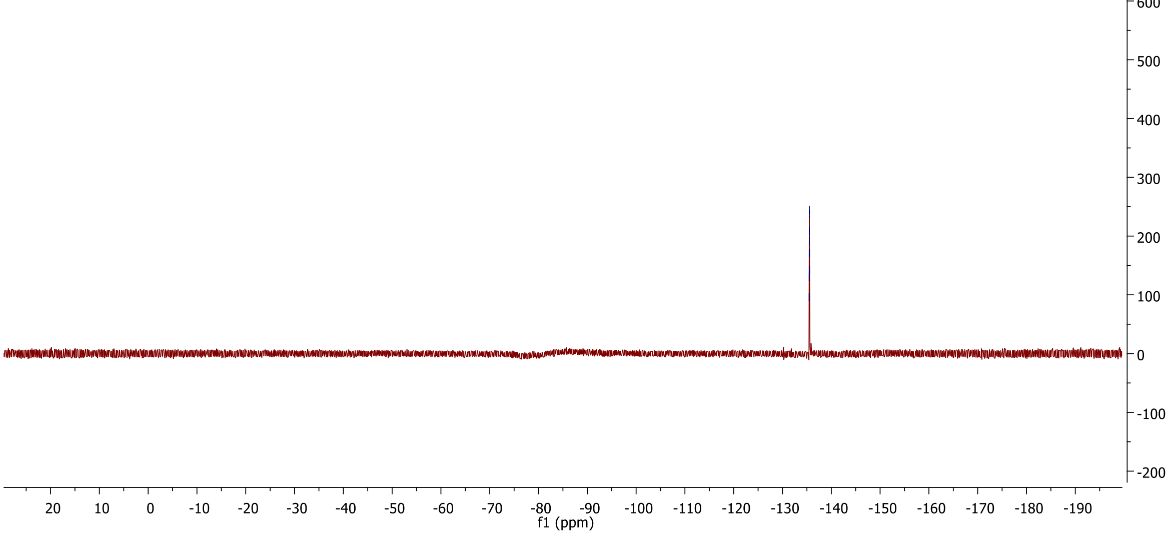
-135.47
-135.48
-135.49
-135.50
-135.51
-135.52
-135.53
-135.54
1000
900
800
700
600
500
400
300
200
100
0
-100
-200
20 10 0 -10 -20 -30 -40 -50 -60 -70 -80 -90 -100 -110 -120 -130 -140 -150 -160 -170 -180 -190
f1 (ppm)

^{1}H-NMR (300 MHz, CDCl$_3$), ^{13}C{^{1}H}-NMR (101 MHz, CDCl$_3$) of *Ethyl 2-(2-methyl-1H-indol-3-yl)-2-(phenylamino)acetate* (3ab)

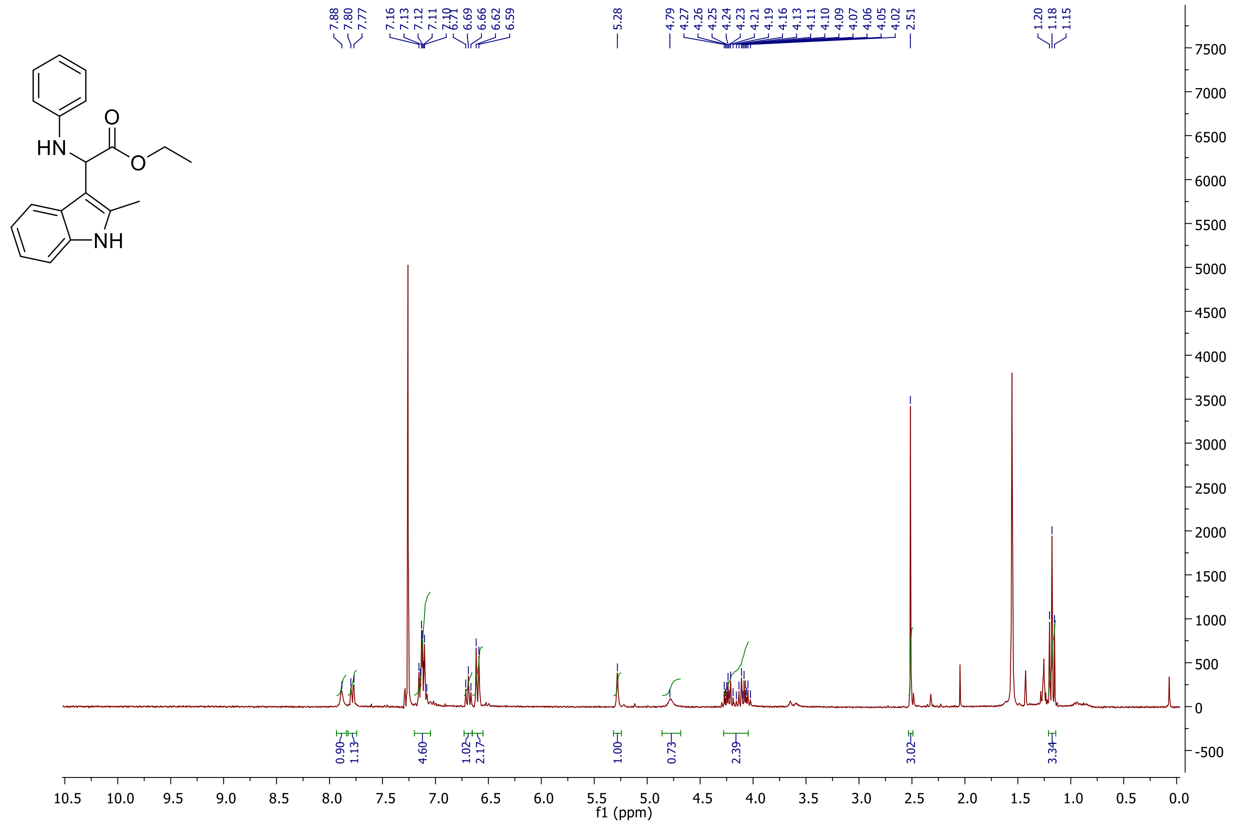

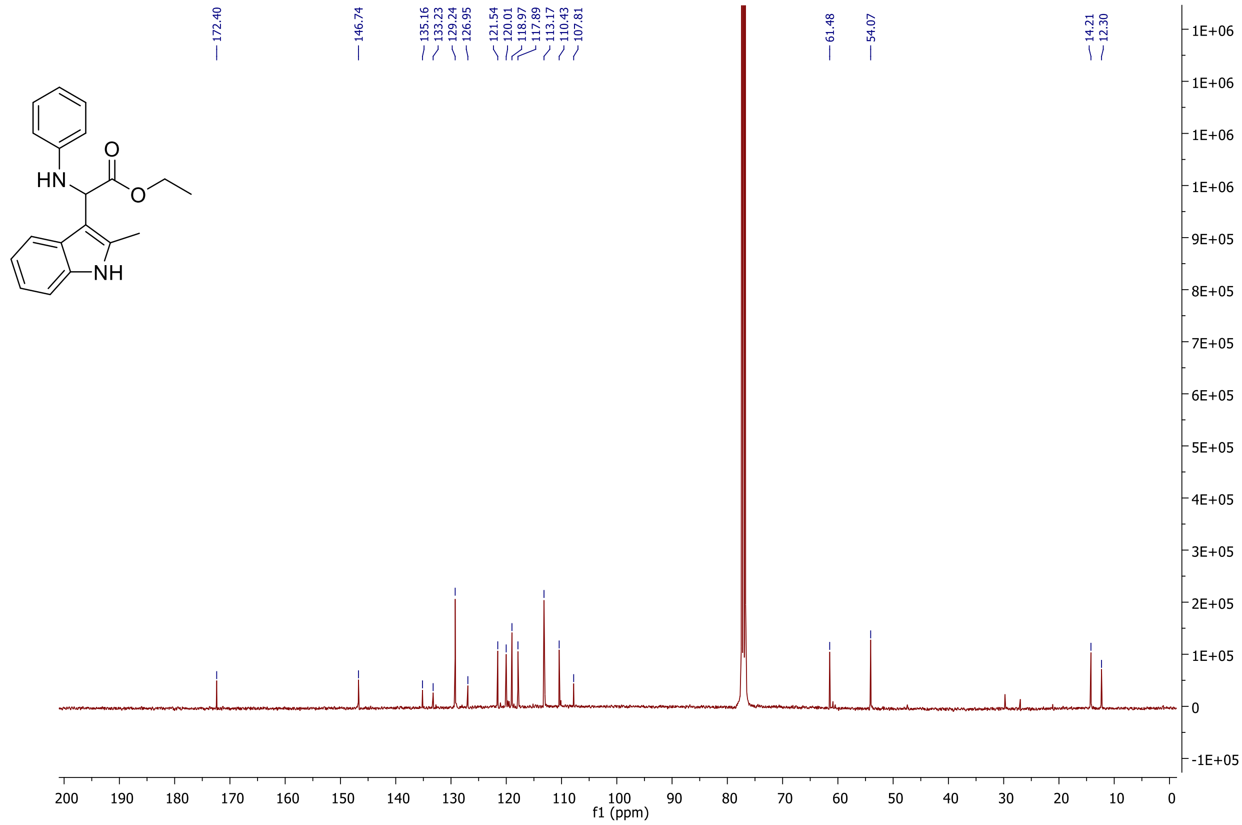

^{1}H-NMR (300 MHz, $CDCl_3$), $^{13}C\{^1H\}$-NMR (101 MHz, $CDCl_3$) of *Ethyl 2-(6-methyl-1H-indol-3-yl)-2-(phenylamino)acetate* (3ac)

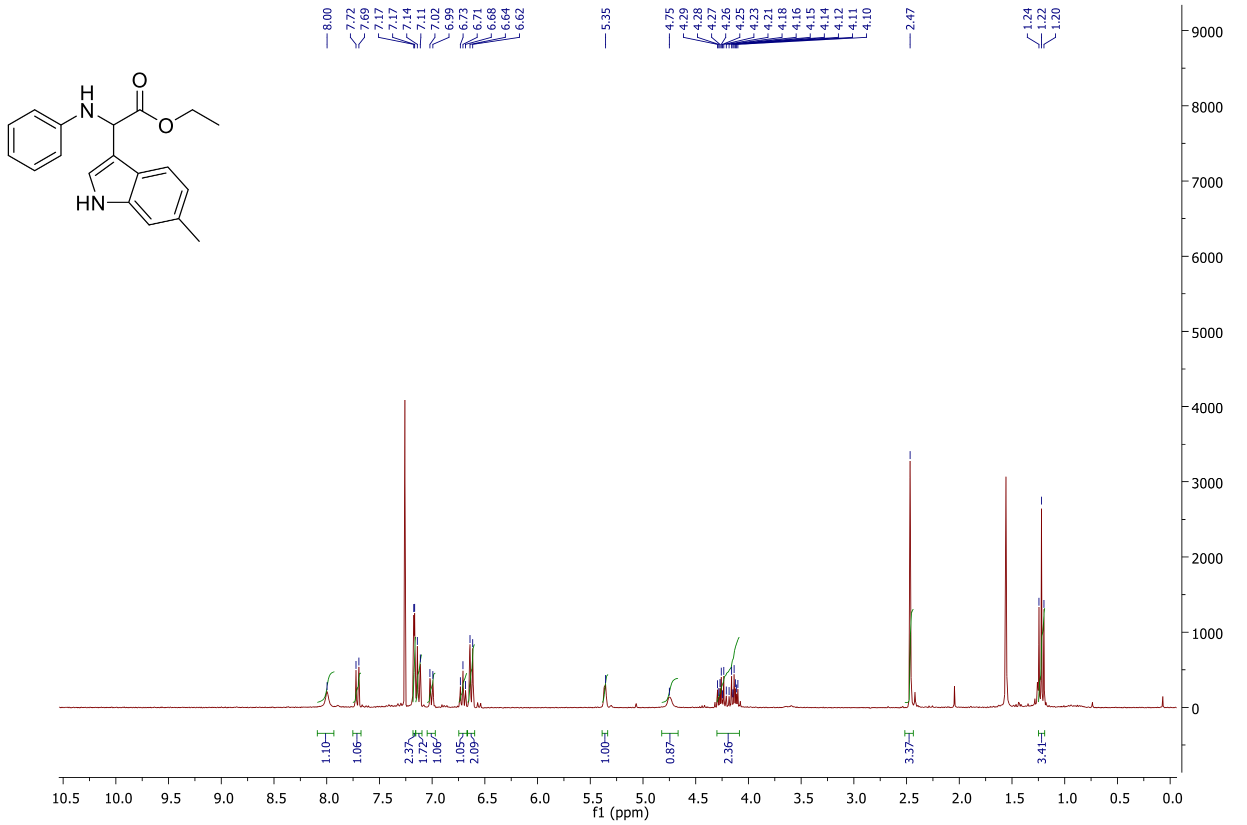

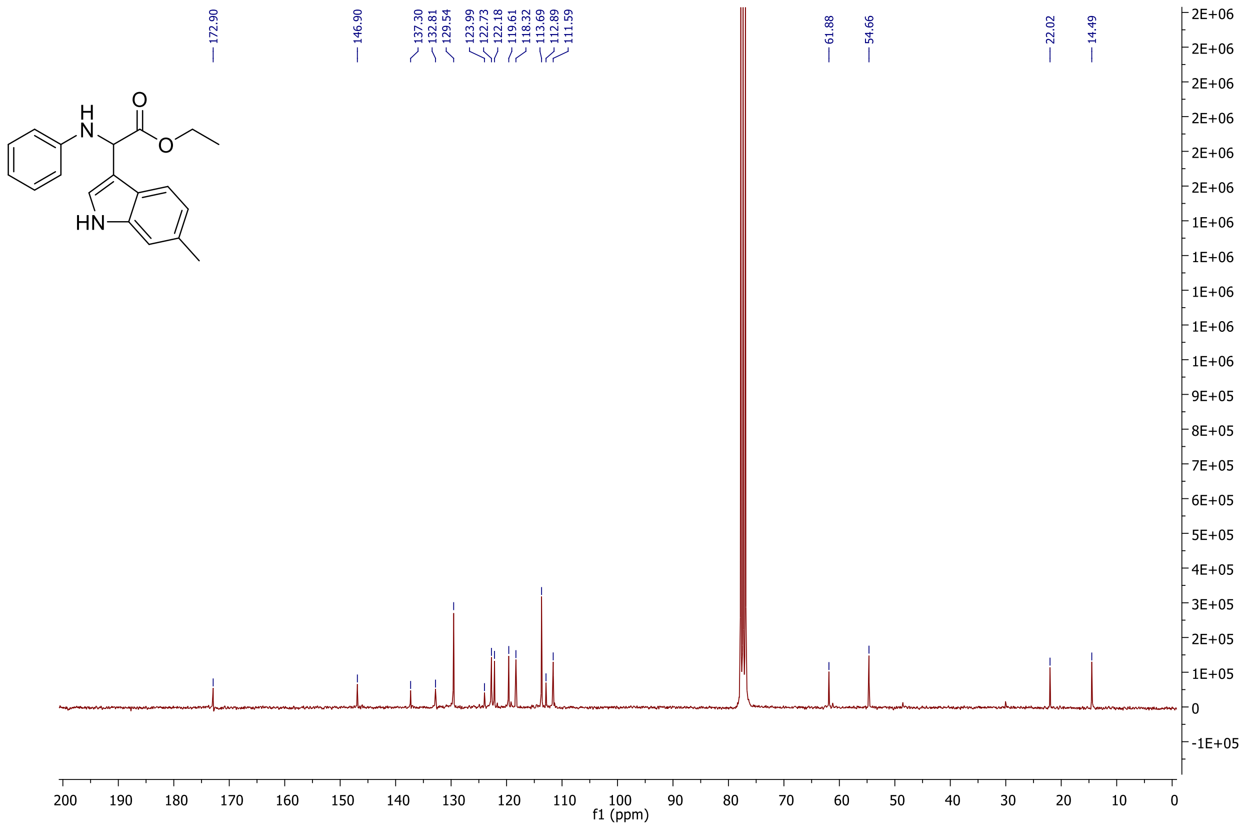

^{1}H-NMR (300 MHz, CDCl$_3$), ^{13}C{^{1}H}-NMR (101 MHz, CDCl$_3$) of *Ethyl 2-(6-methoxy-1H-indol-3-yl)-2-(phenylamino)acetate* (3ad)

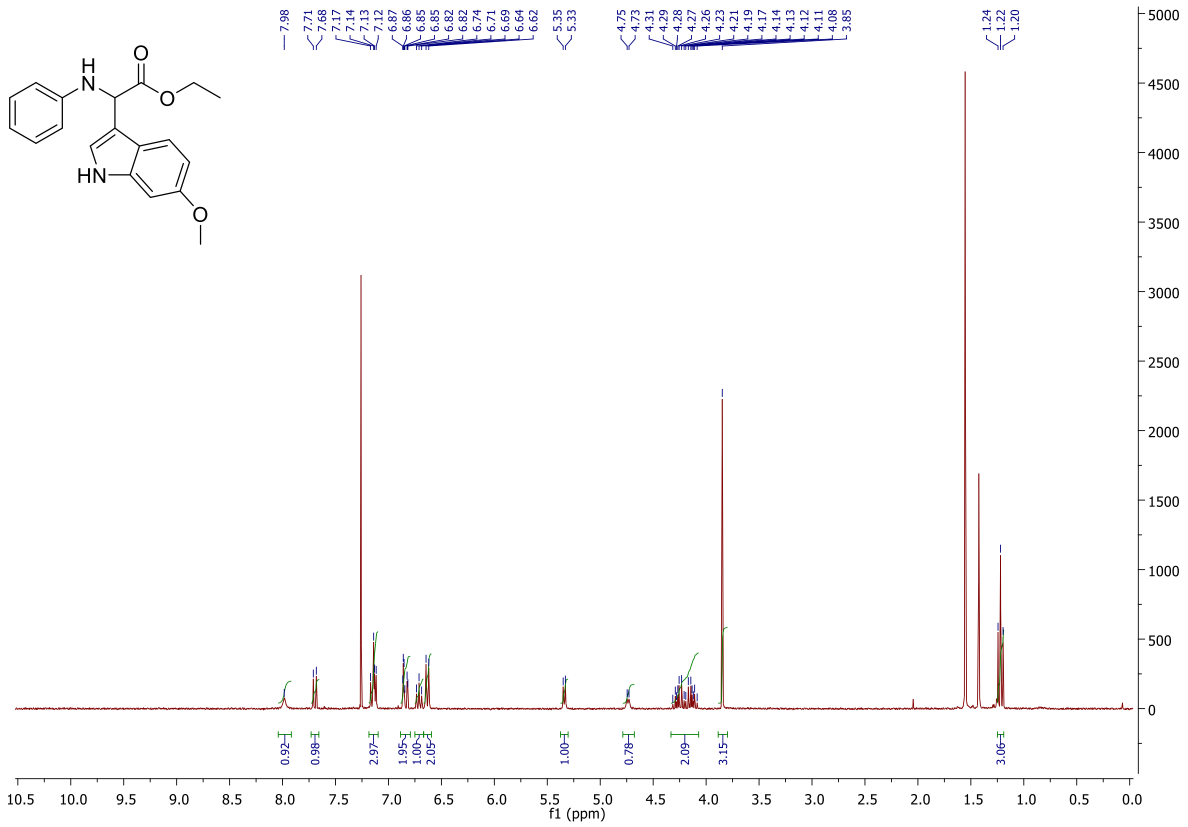

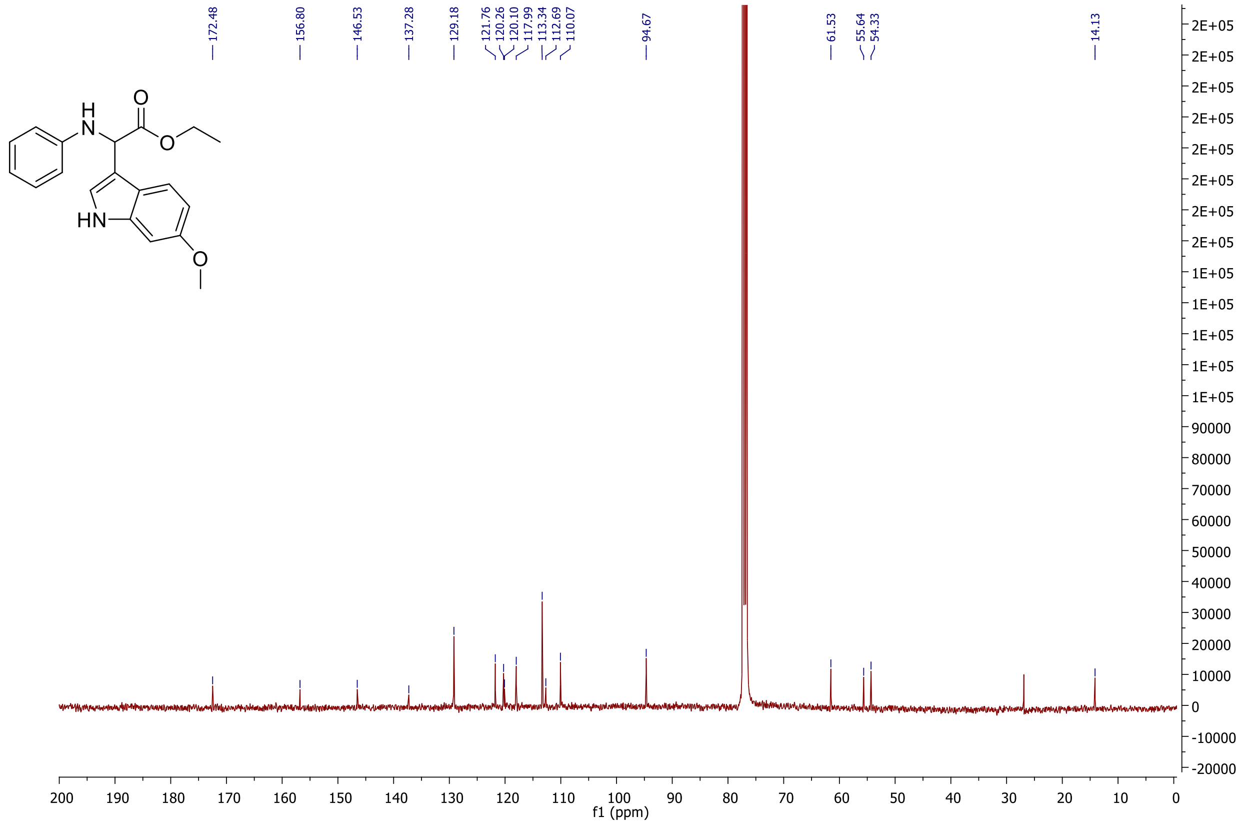

^{1}H-NMR (300 MHz, CDCl$_3$), ^{13}C{^{1}H}-NMR (101 MHz, CDCl$_3$) of *Ethyl 2-(6-fluoro-1H-indol-3-yl)-2-(phenylamino)acetate* (3ae)

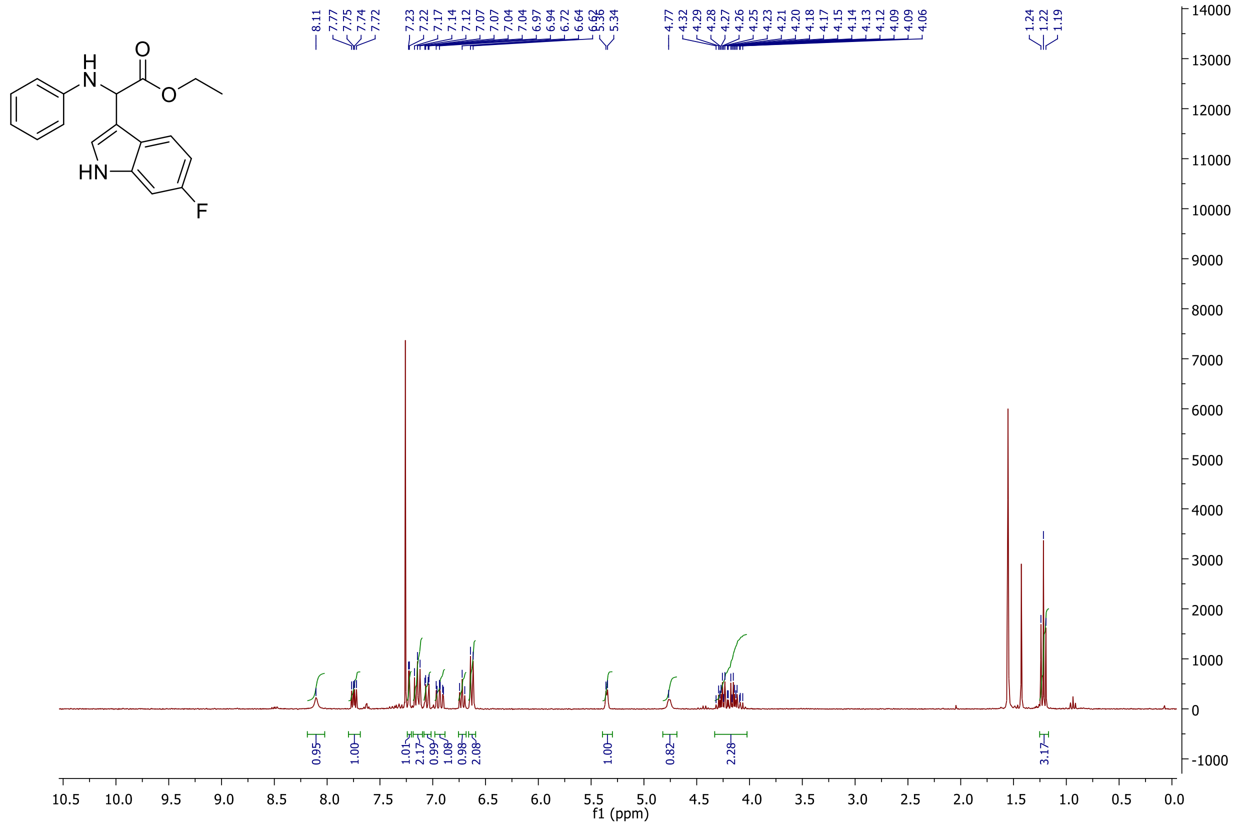

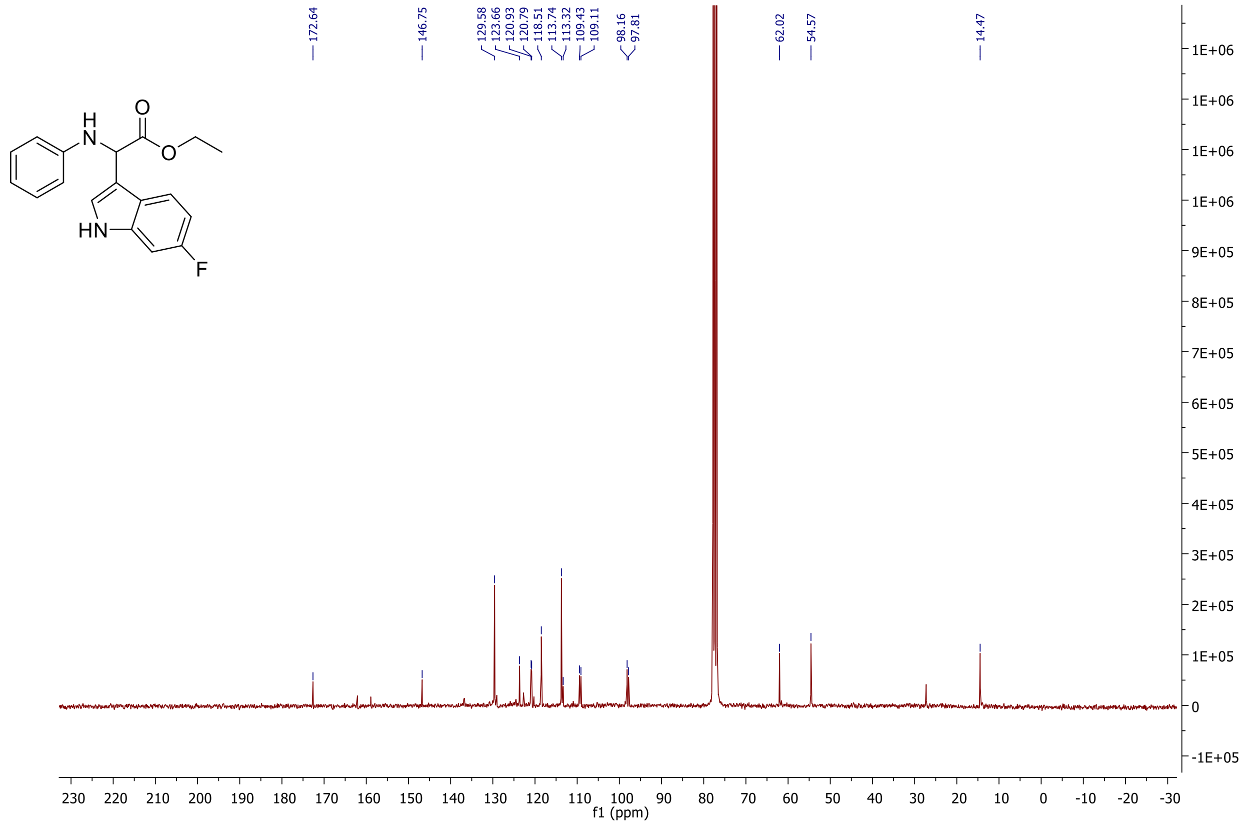

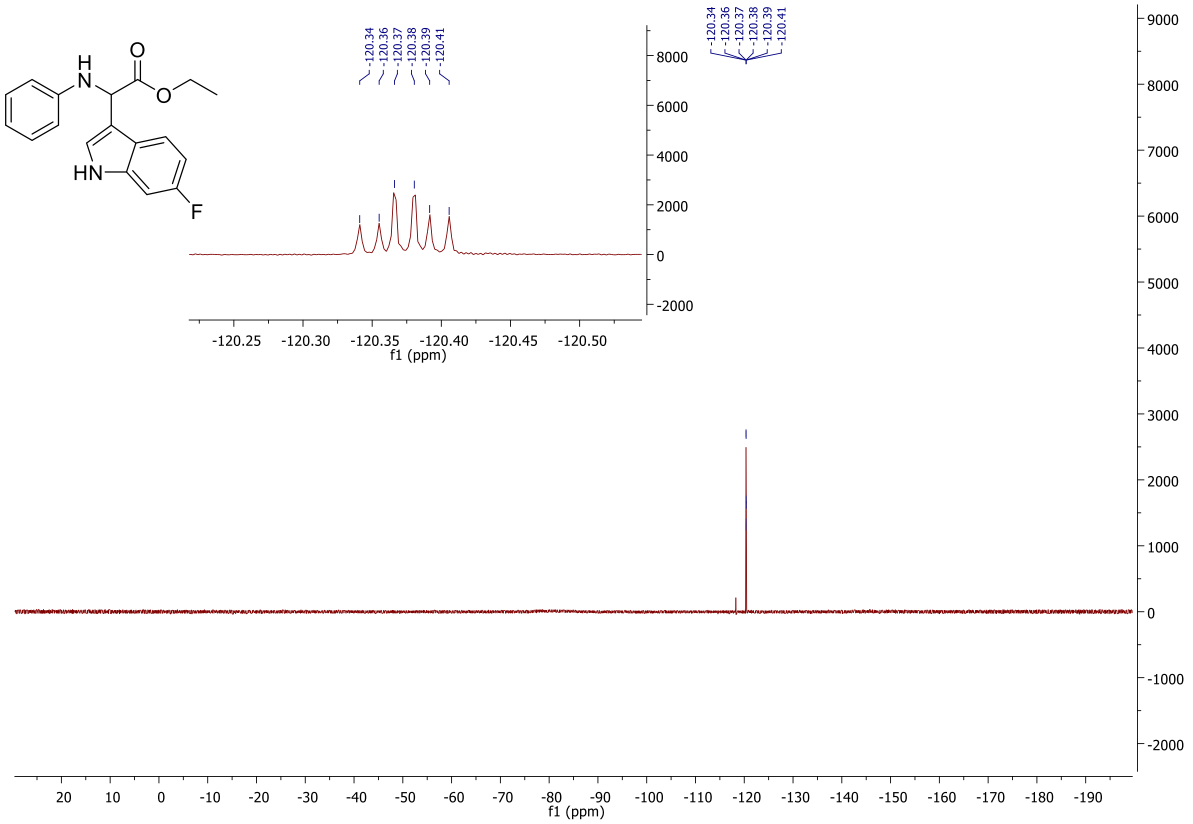

-120.34
-120.36
-120.37
-120.38
-120.39
-120.41
f1 (ppm)
f1 (ppm)

^{1}H-NMR (300 MHz, $CDCl_3$), $^{13}C\{^1H\}$-NMR (101 MHz, $CDCl_3$) of *Ethyl 2-(6-chloro-1H-indol-3-yl)-2-(phenylamino)acetate* (3af)

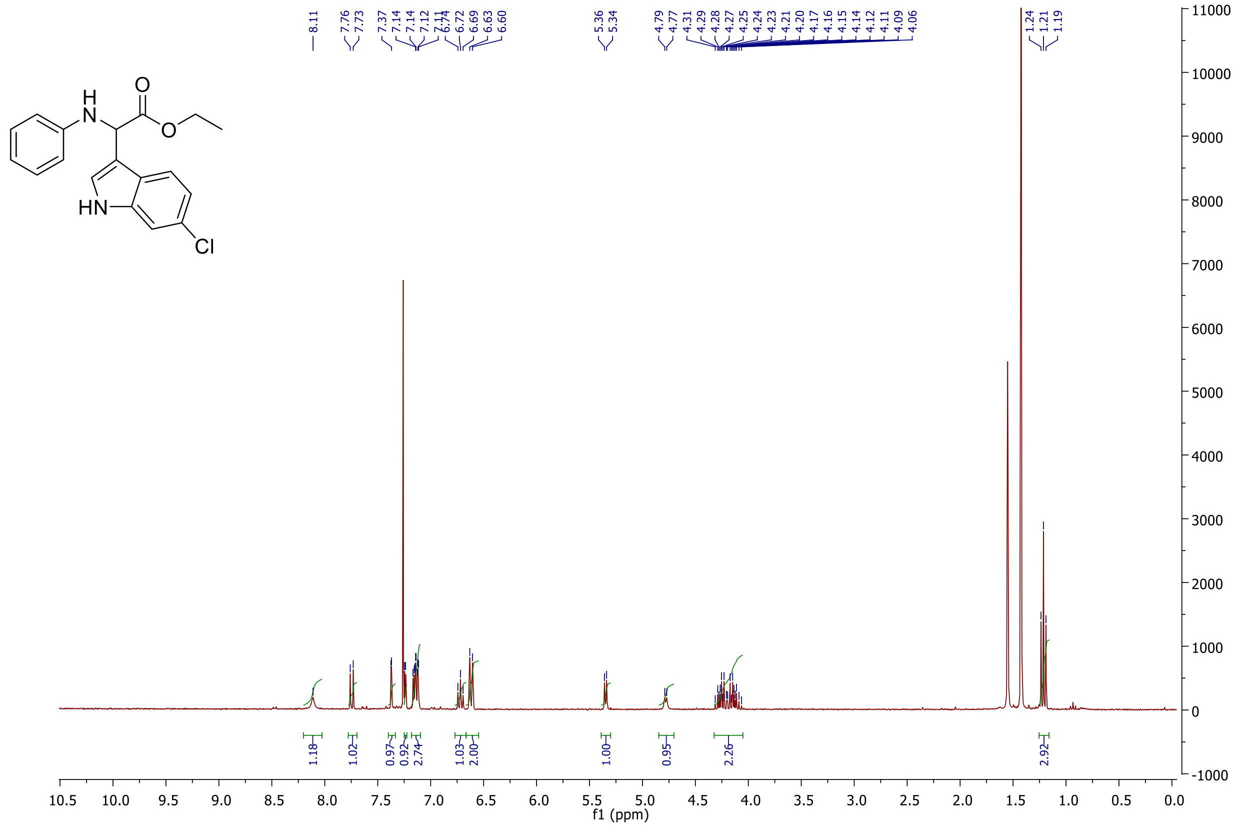

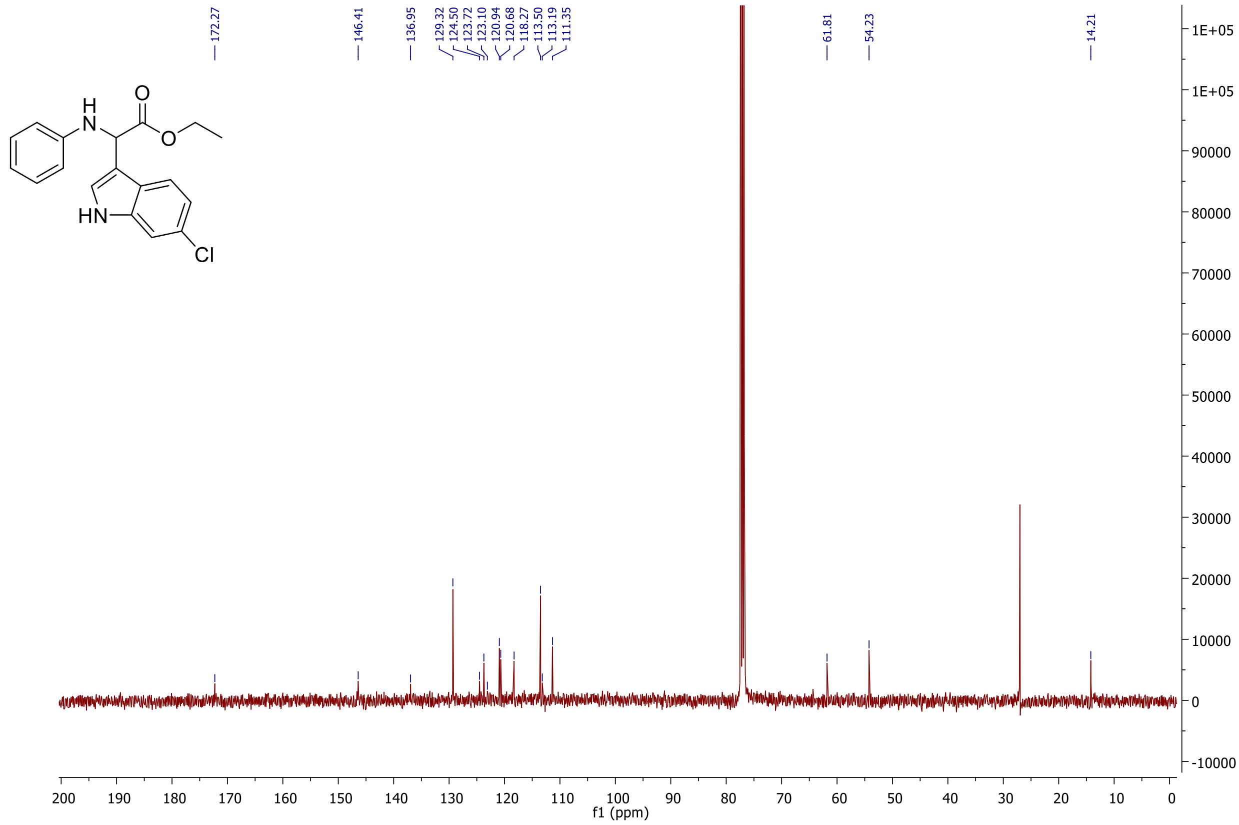

^{1}H-NMR (300 MHz, $CDCl_3$), ^{13}C{^{1}H}-NMR (101 MHz, $CDCl_3$) of *Ethyl 2-(6-bromo-1H-indol-3-yl)-2-(phenylamino)acetate* (3ag)

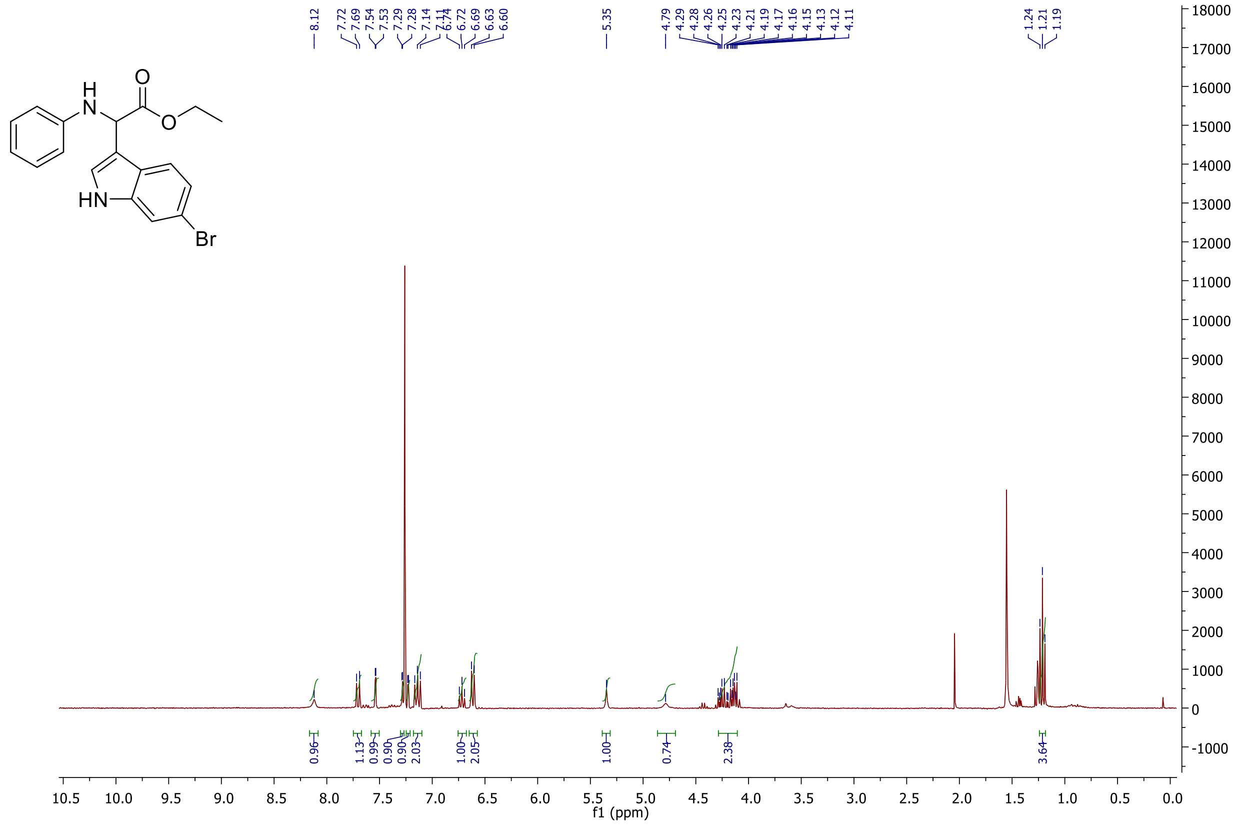

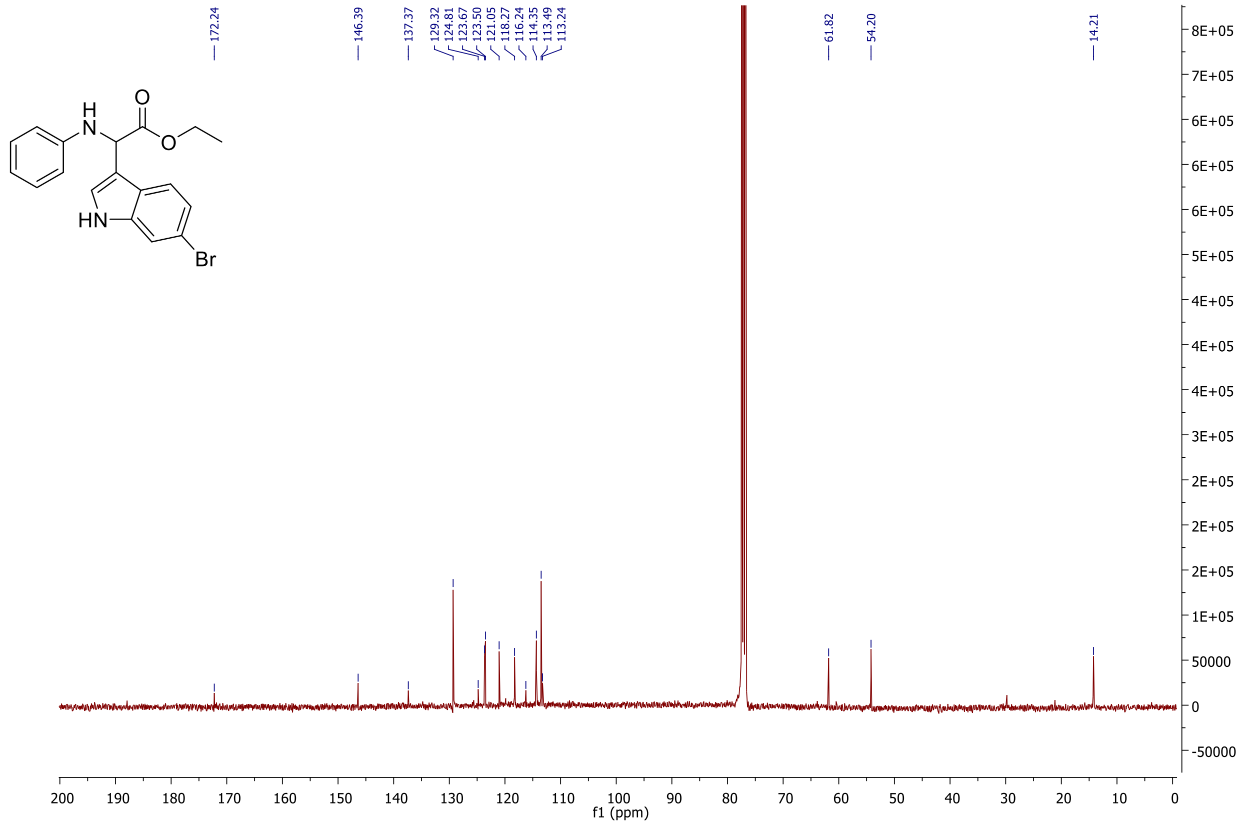

^{1}H-NMR (300 MHz, $CDCl_3$), ^{13}C{^{1}H}-NMR (101 MHz, $CDCl_3$) of *Ethyl 2-(1-methyl-1H-indol-3-yl)-2-(phenylamino)acetate* (3ah)

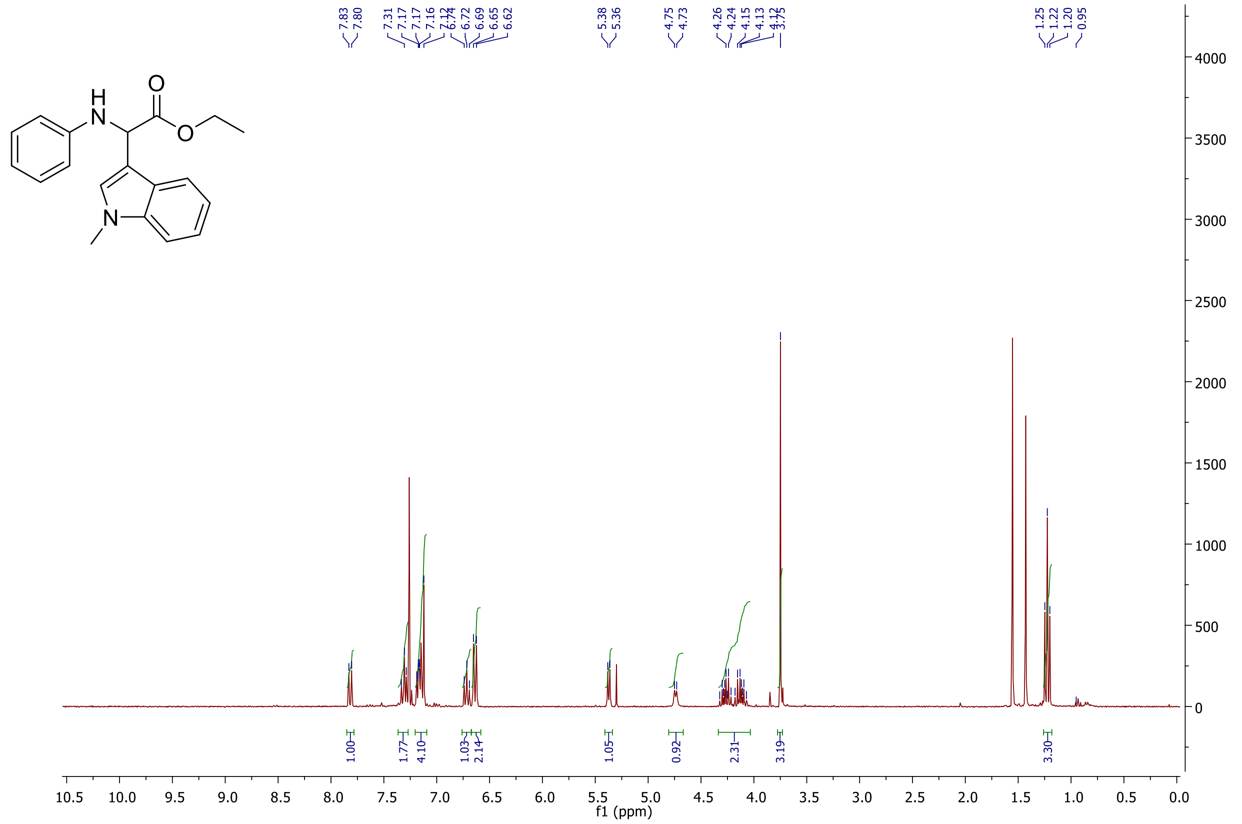

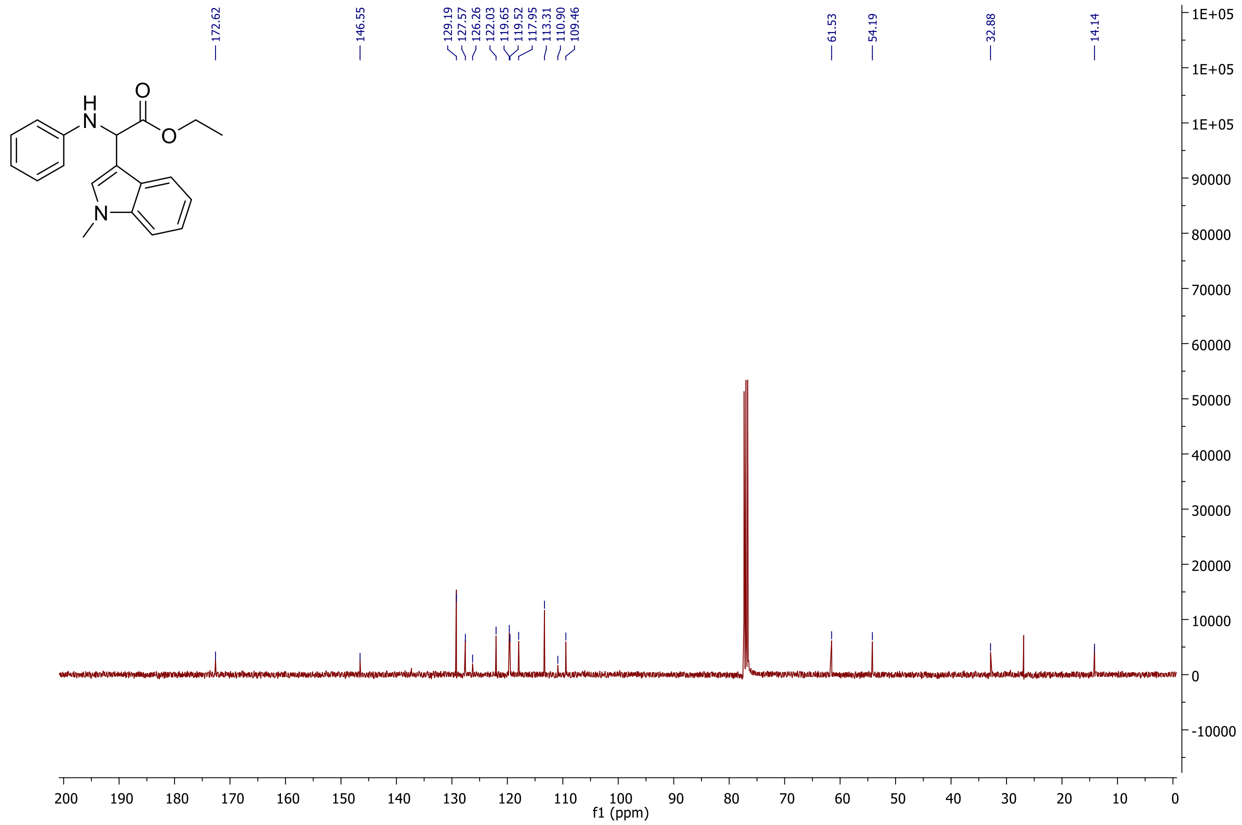

^{1}H-NMR (300 MHz, $CDCl_3$), $^{13}C\{^{1}H\}$-NMR (101 MHz, $CDCl_3$) of *Ethyl 2-(1-benzil-1H-indole-3-yl)-2-(phenylamino)acetate* (3aj)

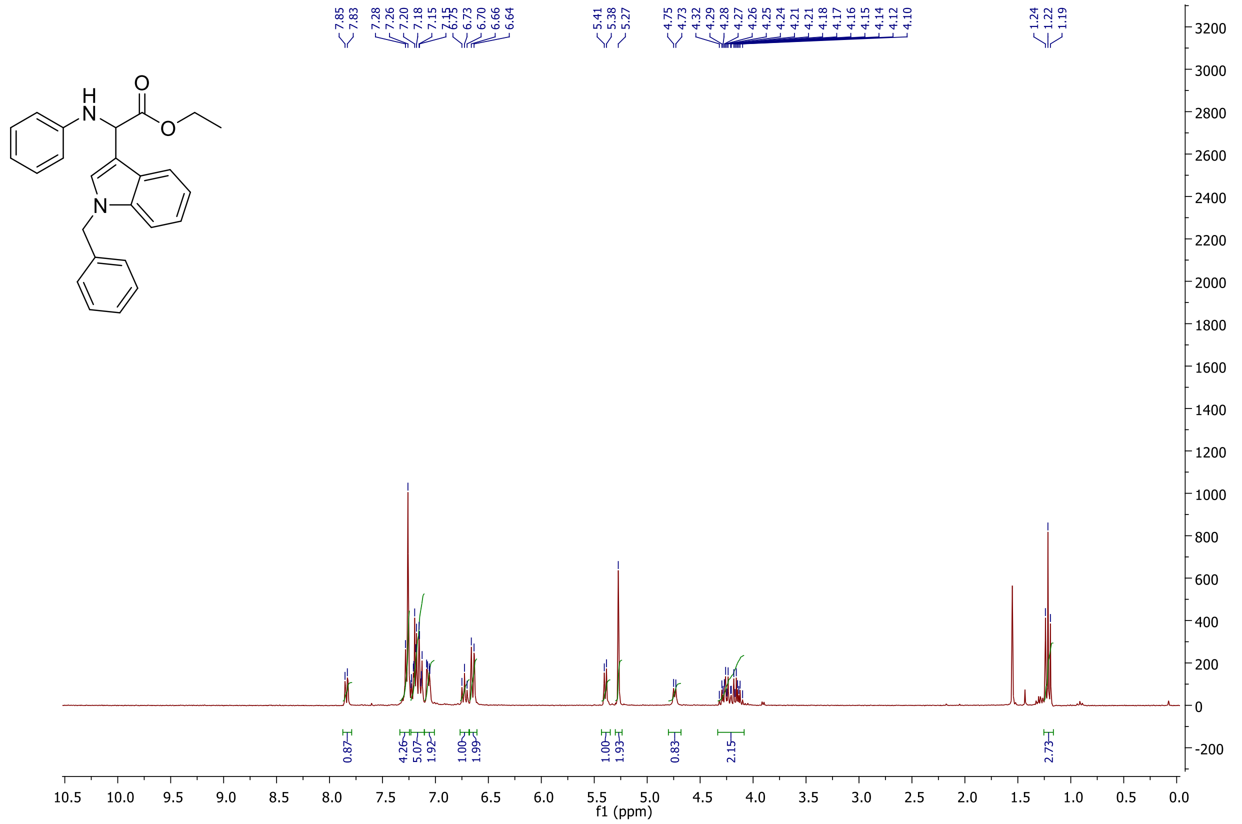

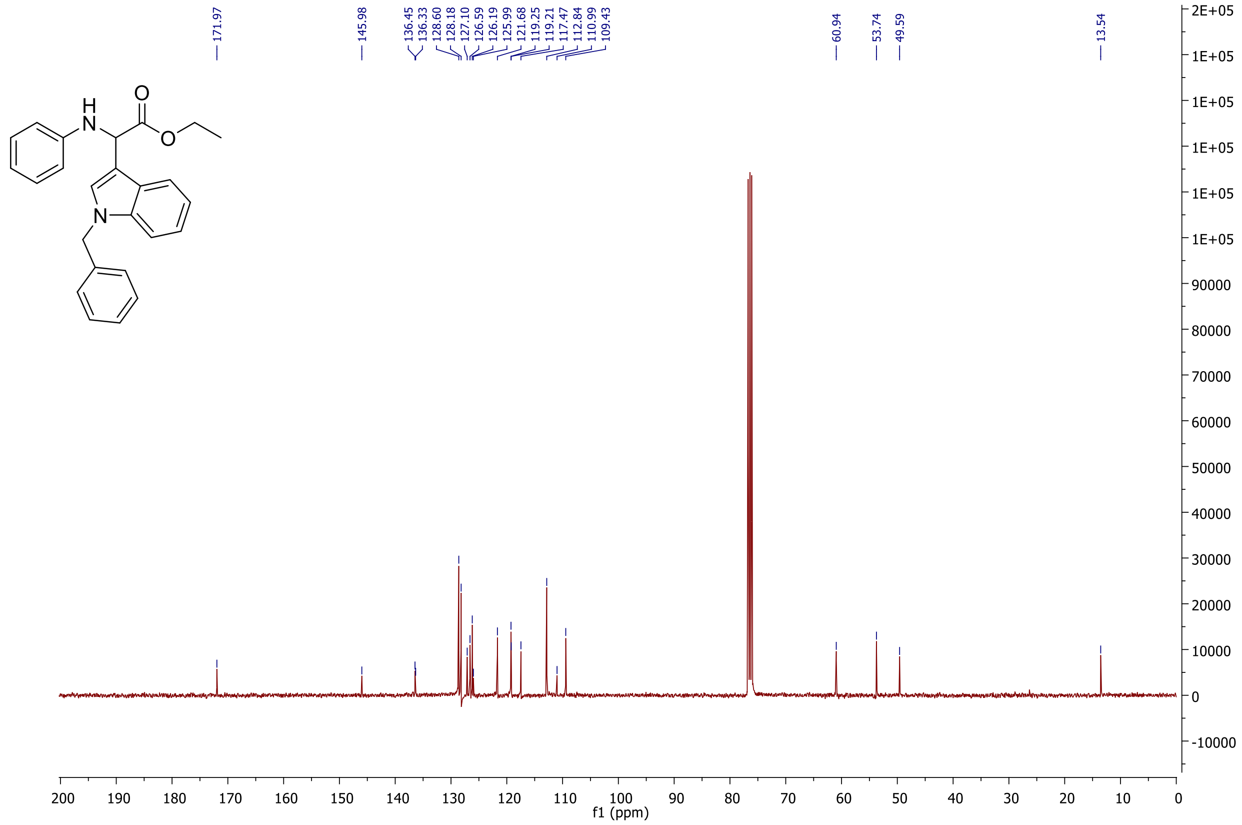

References

[1] Kubelka, P. and Munk, F., Ein Beitrag Zur Optik Der Farbanstriche. *Z. Techn. Phys.*, **1931**, 12, 593 -601.

[2] Pankove, J. I. Optical Processes in Semiconductors; Courier Corporation, 1971
